# Orthogonal Triple-Click Synthesis of Heteromimetic Triglycosylated Fullerenes

**DOI:** 10.1021/acs.orglett.6c02245

**Published:** 2026-07-17

**Authors:** Gema Nieto-Ortiz, Jennifer Patino-Alonso, Natalia Honrubia-Rodríguez, Nazario Martín, M. Ángeles Herranz, Beatriz M. Illescas

**Affiliations:** † Departamento de Química Orgánica, Facultad de Ciencias Químicas, 16734 Universidad Complutense, 28040 Madrid, Spain; ‡ IMDEA-Nanoscience, C/Faraday 9, Campus de Cantoblanco, 28049 Madrid, Spain

## Abstract

Well-defined heteromultivalent glycoarchitectures remain challenging synthetic targets. Herein we report a [60]­fullerene hexakis-adduct platform designed to undergo three orthogonally addressable click reactions, allowing the controlled conjugation of three distinct carbohydrates within a single nanostructure. The approach combines thiol–maleimide coupling with two sequential CuAAC transformations using protected and unprotected alkyne handles and supports both stepwise functionalization and one-pot reaction sequences. NMR and DOSY experiments confirmed the integrity and homogeneity of the resulting triple-functionalized glycofullerenes.

## Graphical abstract

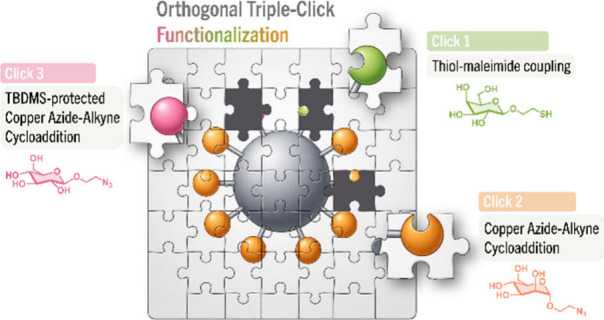

Multivalent systems based on fullerenes have emerged as powerful scaffolds for the presentation of multiple ligands in a globular, three-dimensional architecture. In particular, hexakis-adducts of C_60_ with an octahedral addition pattern offer a unique platform, as Bingel–Hirsch cyclopropanation enables the symmetric installation of six malonate-derived arms around the fullerene core, furnishing up to 12 peripheral functional groups.^1^ These hexakis-adducts can be efficiently synthesized in a one-pot process according to the modification reported by Sun et al.^2^ However, dense functionalization around the C_60_ core renders this transformation highly sensitive to steric hindrance. Consequently, postfunctionalization of preformed hexakis-adducts has become a common strategy, employing transformations such as esterification, amidation, Mitsunobu reactions, and, more recently, click chemistry.^3^

Among click transformations, the copper­(I)-catalyzed azide–alkyne cycloaddition (CuAAC) has been extensively used for fullerene functionalization because of its operational simplicity, broad functional-group tolerance, and high efficiency.^4^ For example, Nierengarten and co-workers reported a *T*_*h*_-symmetric hexakis-adduct bearing 12 azide groups, which were subsequently conjugated with a range of alkyne-terminated building blocks under CuAAC conditions.^5^ This modular strategy enabled the synthesis of multivalent fullerene glycoconjugates, including so-called “sugar balls”, through dense carbohydrate functionalization.^6^

To avoid the limitations associated with copper in biological applications,^7^ alternative click reactions such as strain-promoted azide–alkyne cycloaddition, thiol–ene coupling, anionic alkene–azide cycloaddition (AAAC)^8^ and inverse-electron-demand Diels–Alder cycloadditions have also been implemented on fullerene chemistry.^9^ Accordingly, fullerene scaffolds bearing cyclooctyne, methacrylate, maleimide, azide, or tetrazine groups have been developed to enable orthogonal functionalization strategies.^3b,3c,3e,5,10^

Beyond their synthetic appeal, glycosylated fullerenes have attracted considerable interest because of their potential biomedical applications. The dense and controlled presentation of carbohydrate ligands on a globular nanoscale scaffold enables strong multivalent interactions with carbohydrate-binding proteins, often leading to substantial affinity enhancements compared with monovalent analogues.^11^ As a result, fullerene glycoconjugates have been explored as inhibitors of pathogen adhesion,^6e,12^ antiviral agents,^6c^ lectin-targeting probes,^13^ and modulators of glycosidase activity.^6d,6f^ Moreover, the incorporation of multiple addends on a fullerene scaffold enables the construction of synergistic architectures and multifunctional hybrids by combining different ligands within a single nanostructure. This versatility provides a natural entry point to heteromultivalent design strategies. Beyond simple multivalency, heteromultivalency—the simultaneous presentation of different ligands on one platform—offers a powerful strategy to better emulate the complexity of biological recognition processes and to promote cooperative binding effects.^4,14^ Fullerene-based heteromultivalent systems have already demonstrated enhanced recognition and inhibitory properties toward lectins and glycosidases compared to homovalent analogues.^12,15^ In this context, orthogonal click chemistry represents an attractive synthetic approach, as it enables the selective and sequential installation of different ligands in a controlled manner.^14^ Despite this potential, examples of fullerene platforms specifically designed to undergo multiple orthogonal click reactions remain scarce,^3c,16^ and only one previous study has described a fullerene scaffold capable of three sequential click transformations.^10^ In addition to orthogonal postfunctionalization strategies, selective full­er­ene derivatization has been achieved through regioselective addition methodologies relying on covalent or supramolecular masking of the carbon cage.^17^ These approaches provide powerful control over the addition pattern of the fullerene core and are complementary to the orthogonal functionalization strategies described herein.

In this work we report a C_60_-based platform engineered to undergo three mutually orthogonal click reactions, through the combination of thiol–maleimide coupling and two sequential CuAAC transformations. This strategy affords unprecedented control over the spatial and chemical presentation of three different carbohydrates on a globular scaffold, thereby providing access to well-defined heteromimetic architectures through both sequential and one-pot protocols.

The hexakis-adduct building block **5** bearing three orthogonal reactive centers was synthesized as outlined in Scheme 1. The nonsymmetrical malonate **1**, featuring a TBDMS-protected alkyne and a protected maleimide unit, was first prepared through a multistep procedure.^16d,18^ Protection of the maleimide functionality as its furan Diels–Alder cycloadduct prevented competing reactions, and deprotection was carried out only immediately prior the thiol–maleimide addition reaction (see Scheme S1). This malonate reacted with C_60_, iodine, and DBU under typical Bingel–Hirsch cyclopropanation conditions, affording monoadduct **2**. Subsequent Bingel reaction with excess terminal alkyne-substituted di­(pent-4-yn-1-yl) malonate (**3**), CBr_4_ and DBU furnished the asymmetric hexakis-adduct **4**. A final retro-Diels–Alder reaction by refluxing in toluene released the maleimide functionality, yielding the target hexakis-adduct **5** bearing three different reactive subunits. All new compounds were characterized using standard spectroscopic methods (see the Supporting Information).

**Scheme 1:**
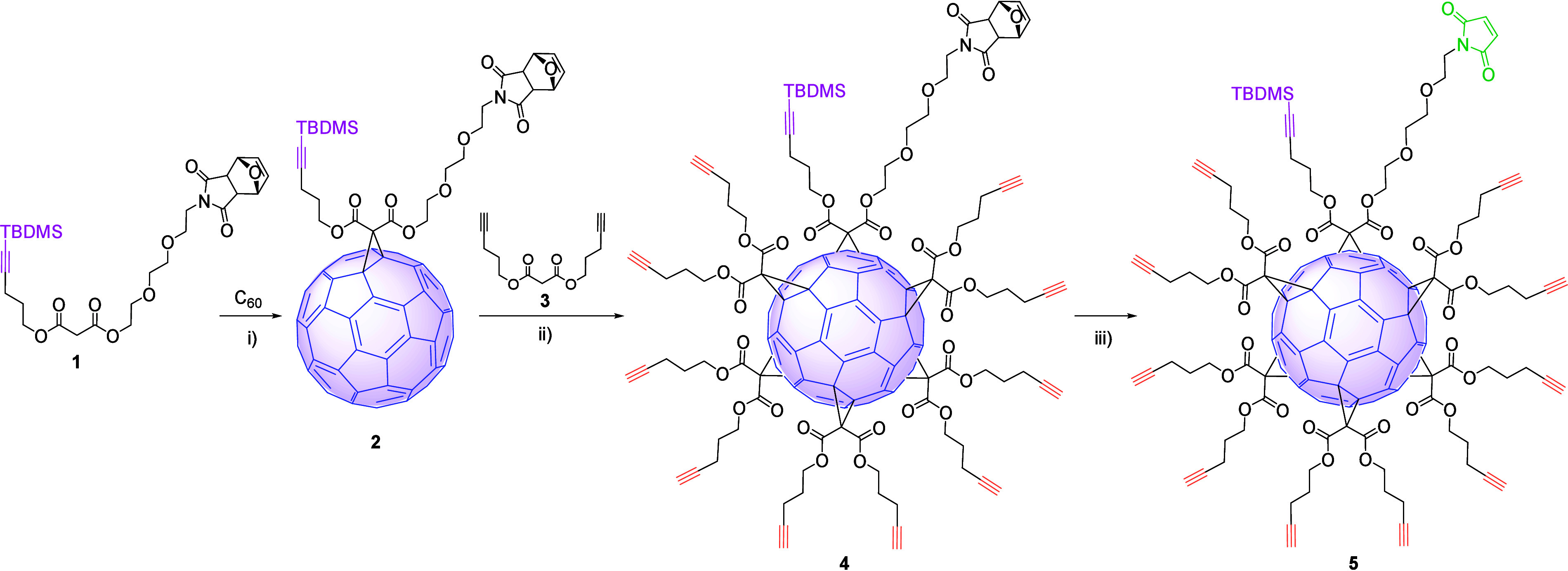
Synthetic Pathway to Obtain Hexakis-adduct **5** ^a^

a Reagents and conditions: (i) I_2_, DBU, dry toluene, 0 °C → r.t., 4 h (53%); (ii) **3**, CBr_4_, DBU, dry toluene, 72 h (72%); (iii) dry toluene, 120 °C, overnight (quant.).

The ^13^C NMR spectrum of compound **5** (Figure 1a) displays two characteristic sp^2^ carbon signals at 141 and 145 ppm, confirming the effective hexasubstitution of the fullerene core, which confers high symmetry (*T*_*h*_). In addition, resonances corresponding to the functionalities introduced by the two types of malonates are evident. The olefinic carbons of the maleimide unit appear at 134 ppm, while the terminal alkyne carbons are observed at ∼82 and ∼78 ppm. At slightly higher chemical shifts, signals corresponding to the TBDMS-protected alkyne are detected at ∼106 and 84 ppm.

**Figure 1:**
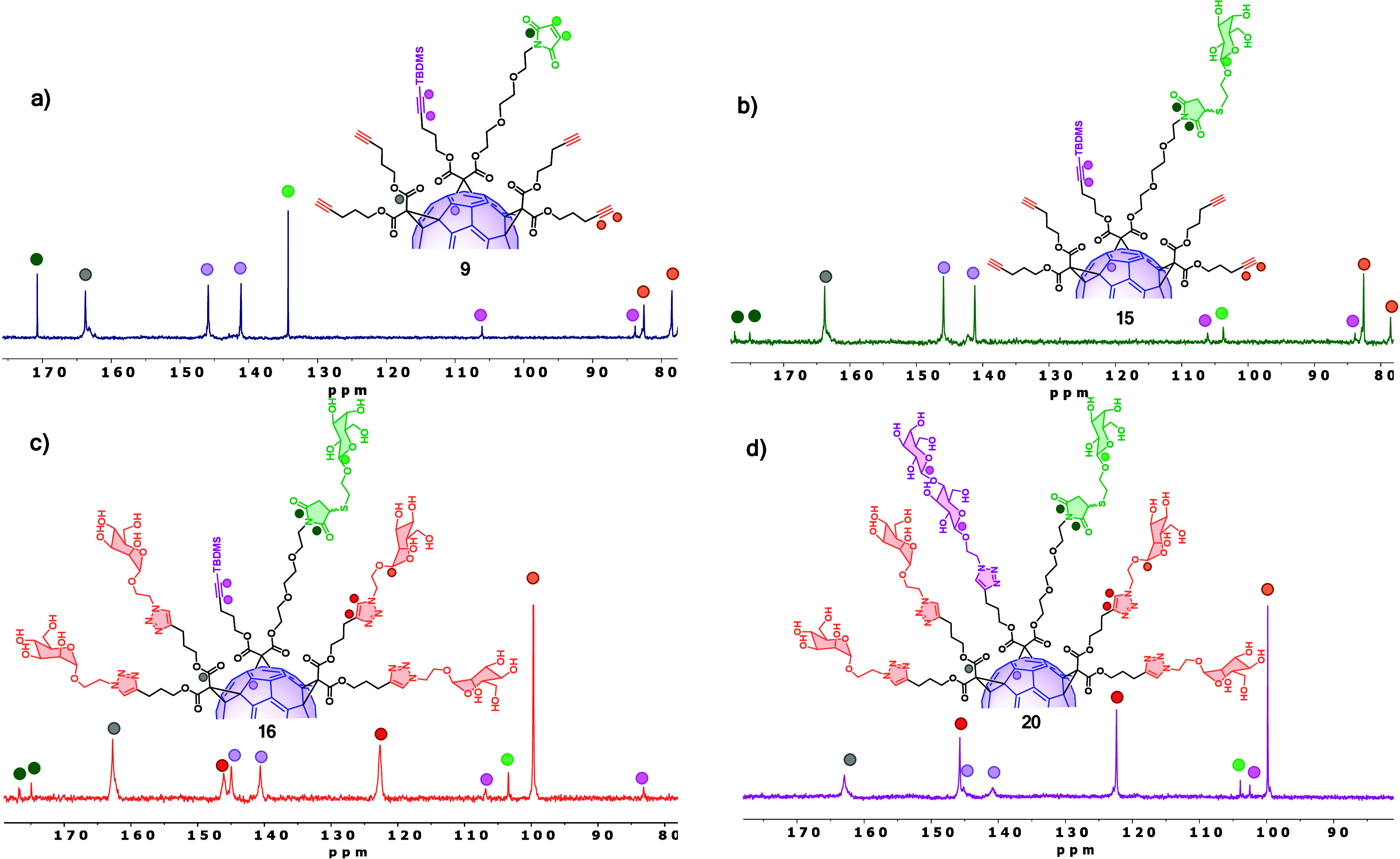
Partial view of the ^13^C NMR spectra (176 MHz) of (a) **9** (CDCl_3_), (b) **15** (CDCl_3_), (c) **16** (DMSO-*d*_6_), and (d) **20** (DMSO-*d*_6_).

Before incorporating selected carbohydrates to construct the desired heteromultivalent derivatives, the versatility and efficiency of the click reactions on building block **5** were evaluated using commercial and easily available reagents (see Scheme S2). In this proof of concept, the optimal sequence of click additions was established. When the azide was introduced first, resulted in undesired reactivity with the maleimide moiety.^16d^ Therefore, the sequence begins with the Michael-type addition, followed by the CuAAC reaction. Thus, reaction with 1-octanethiol was carried out in dry CH_2_Cl_2_ at room temperature for 24 h and was followed by CuAAC with the mannose-azide derivative **11**. By this procedure, ten units of mannose were conjugated to the hexakis-adduct, which made the product water-soluble. Finally, *in situ* TBAF deprotection regenerated the remaining alkyne functionality, enabling a second CuAAC reaction. This second copper-catalyzed click reaction employed benzyl azide and was performed under the same experimental conditions, using CuBr·SMe_2_ and sodium ascorbate as catalyst and reductor respectively, in DMSO at room temperature.^11,19^ The same synthetic strategy was employed to introduce three different carbohydrates to obtain heteromultivalent systems, examining both stepwise and one-pot approaches. First, in the sequential route (Scheme 2), hexakis-adduct **9** underwent thiol–maleimide coupling with thiol-functionalized galactose derivative **14** in the presence of Et_3_N as a base, affording compound **15** in 77% yield. The ^13^C NMR spectrum of this compound (Figure 1b) showed the disappearance of the olefinic carbons from the maleimide unit (δ∼134) and the appearance of the characteristic anomeric carbon signal of galactose at ∼104 ppm, confirming the successful first click transformation. Furthermore, this last signal appears as a double set because the thiol–maleimide reaction generates a new stereogenic center, leading to a mixture of two diastereoisomers. Additionally, the olefinic resonances of the maleimide group in ^1^H NMR, initially visible at ∼6.6 ppm, disappear upon reaction, confirming full conversion.

**Scheme 2:**
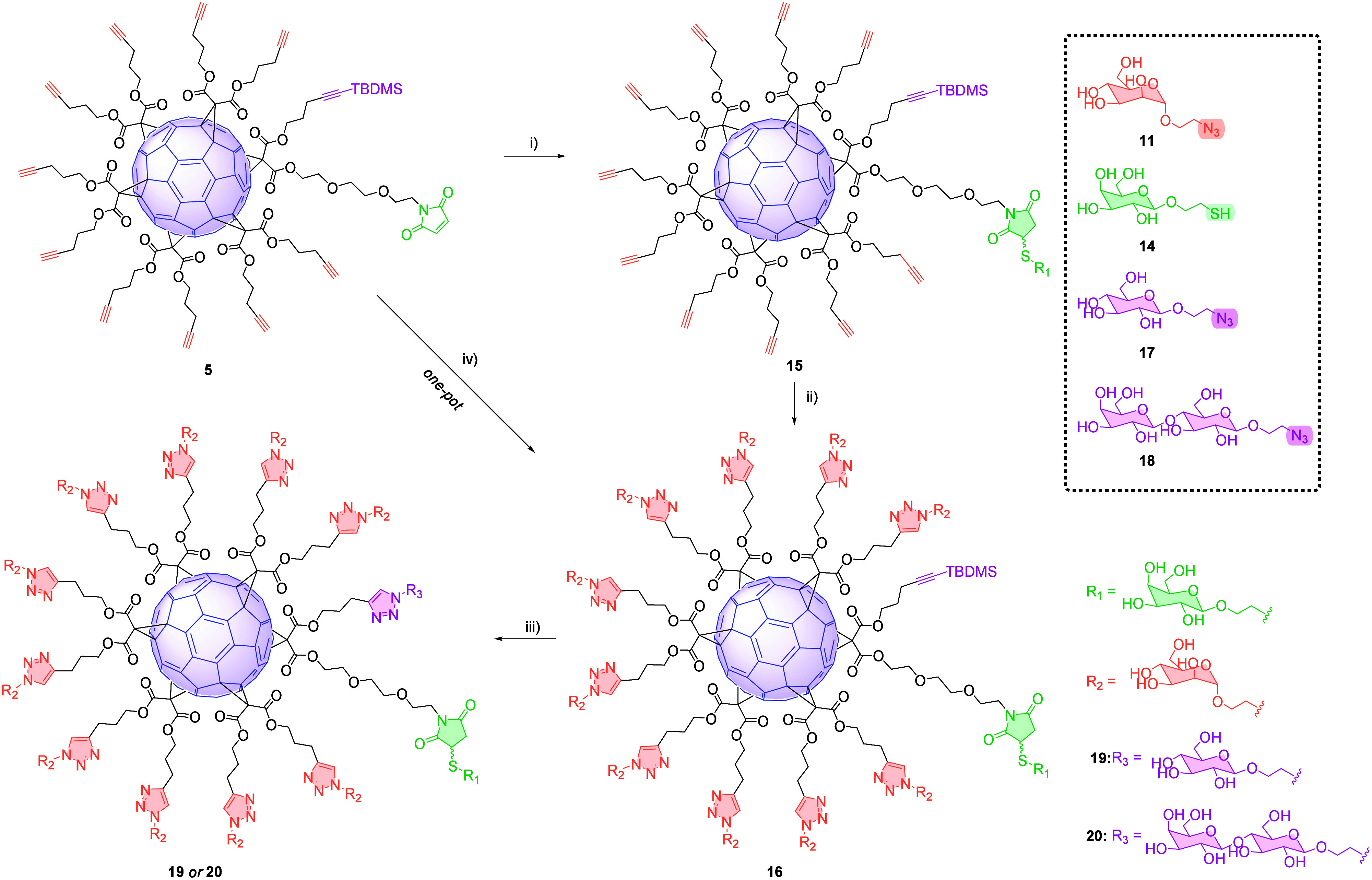
Synthetic Pathway to Obtain Compounds **19** and **20** ^a^

a Reagents and conditions: (i) **14**, Et_3_N, dry DCM/MeOH, r.t., overnight (77%); (ii) **11**, CuBr·SMe_2_, Cu^0^, AscNa, DMSO, r.t., 72 h (80%); (iii) (1) TBAF, DMSO, 30 °C, 24 h, (2) **17** or **18**, CuBr·SMe_2_, Cu^0^, AscNa, r.t., 24 h (**19**, 34%; **20**, 51%); (iv) (1) **14**, Et_3_N, DMSO, overnight, (2) **11**, CuBr·SMe_2_, Cu^0^, AscNa, r.t., 72 h (86%).

Then mannose-azide derivative **11** was coupled to the peripheral alkynes of hexakis-adduct **15** under CuAAC conditions, affording glycofullerene **16** in excellent yield (80%). The new ^13^C NMR signals (Figure 1c) at 146 and 120 ppm, corresponding to the newly formed triazole ring, together with the anomeric carbon signal of the mannose unit (∼100 ppm), confirm the successful formation of compound **16**. The ^1^H NMR spectrum also displays a broad resonance at 7.8 ppm, attributed to the triazole ring proton.

The next step involved deprotection of the TBDMS group using TBAF, which proceeded to complete conversion but afforded a low isolated yield, likely due to the formation of aggregate-type byproducts. As a result, the ^13^C NMR spectrum showed reduced resolution, although the disappearance of the *tert*-butyl and methyl signals of the TBDMS protecting group at their respective chemical shifts (∼26 and −5 ppm) was clearly observed.

Therefore, we decided to carry out the desilylation and the subsequent CuAAC reaction in a one-pot sequence, without isolation of the intermediate deprotected alkyne. Thus, once the alkyne was generated *in situ*, the reaction mixture was directly subjected to a second CuAAC reaction with the terminal azides of the glucose (**17**) and lactose (**18**) derivatives, affording compounds **19** and **20** in moderate yields (34% and 51%, respectively). Formation of this new triazole ring can be observed in ^1^H NMR spectra, where two distinct signals can be detected for the two different types of triazole units present in the structure. ^13^C NMR data also support the formation of the triple-clicked products. For example, for compound **20** (Figure 1d) three well-resolved signals appear for the four carbohydrate anomeric carbons. The disappearance of the characteristic resonances of the previously protected alkyne (∼107 and 83 ppm) further confirms both the desilylation and the subsequent click reaction. Additionally, the ^1^H–^13^C HSQC spectra (Figure 2) provided more detailed structural information on the anomeric carbons than the ^13^C NMR spectra alone, displaying different cross-peaks between 99 and 105 ppm that correspond to each carbohydrate introduced into the heteromultivalent platform—three resonances for compound **19** and four for compound **20**. Furthermore, the purity and integrity of the final compounds were further supported by diffusion-ordered spectroscopy (DOSY) NMR experiments (ca. 2 mg mL^–1^ in D_2_O, Figures S42 and S48), which showed a single diffusion coefficient for all resonances, consistent with the presence of a single major species in solution.

**Figure 2:**
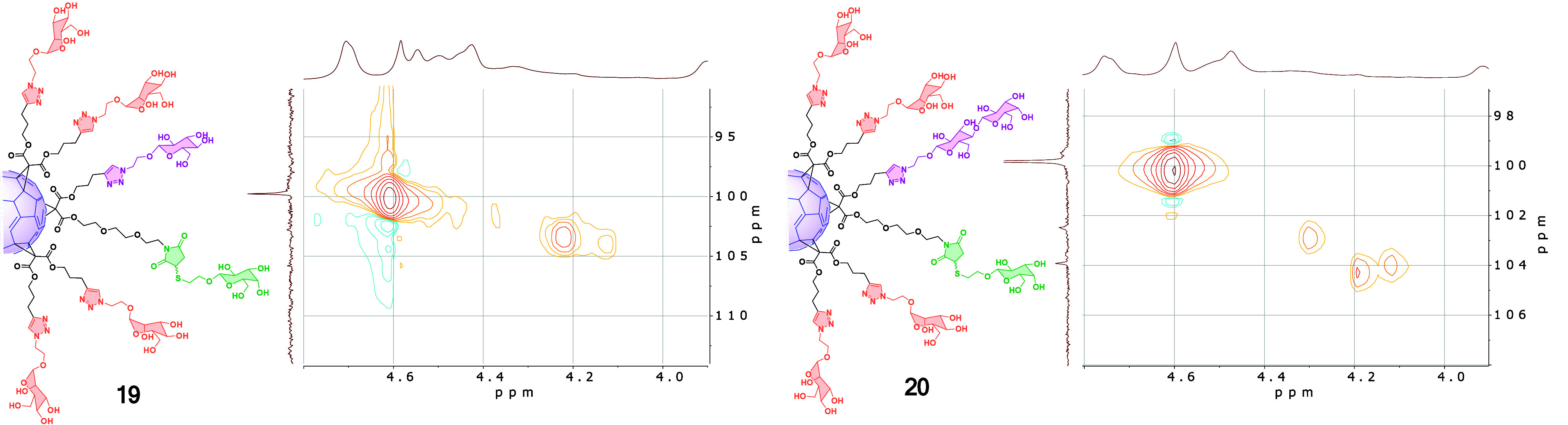
Zoom of ^1^H–^13^C HSQC NMR spectra (700 MHz, DMSO-*d*_6_) of compounds **19** and **20**.

The UV–vis spectra of the fullerene derivatives are consistent with the proposed degree of functionalization (see the Supporting Information). While compound **2** displays the characteristic absorption features of a Bingel monoadduct, including the weak band around 430 nm, the hexakis-adducts exhibit the typical spectral profile of highly functionalized C_60_ derivatives, with the disappearance of this band and absorption mainly confined to the UV region.

Alternatively, a one-pot strategy was evaluated (Scheme 2). Because of the excess azide required for the second click step, only the first two transformations could be performed without intermediate purification. Thus, the thiol–ene addition was carried out first and, after stirring overnight, mannose-azide derivative **11** was added together with the catalyst and sodium ascorbate. After an additional 72 h, compound **16** was isolated in 86% overall yield. According to the procedure described above, a second one-pot protocol was also developed for TBDMS deprotection and the final CuAAC step. This approach afforded compounds **19** and **20** in a higher overall yield (29% and 44%) than the stepwise sequence (21% and 31%). In both cases, DMSO was used as the solvent, and purification of intermediate **15** was avoided. Compounds **16**, **19**, and **20** were treated with QuadraSil MP to remove residual copper and the crudes were purified by size-exclusion chromatography.

Overall, these one-pot sequences demonstrate the practicality and robustness of the orthogonal triple-click strategy, enabling the efficient construction of structurally complex heteromimetic triglycosilated fullerenes in a streamlined and operationally simple manner.

In summary, we have developed a [60]­fullerene-based molecular platform that undergoes three fully orthogonal click reactions, enabling the controlled assembly of heteromultivalent glycofullerenes that display three distinct carbohydrate ligands on a single nanoscopic scaffold. Combining thiol–maleimide chemistry with two sequential CuAAC reactions—enabled by temporary alkyne protection—provides a modular route to densely and diversely functionalized fullerene hexakis-adducts, with both stepwise and one-pot reaction sequences proving equally effective. NMR and DOSY analyses provided complementary evidence supporting the structural integrity of the resulting glycofullerenes and the successful implementation of each functionalization step. To the best of our knowledge, this constitutes the first molecular scaffold explicitly designed for three orthogonal click reactions applied to the construction of well-defined heteromimetic triglycofullerenes. The methodology is readily extendable to other biomolecules and functional motifs, offering a general blueprint for exploring heteromultivalency in lectin recognition and glycobiology.

## Supplementary Material



## Data Availability

The data underlying this study are available in the published article and its Supporting Information.

## Notes

General methods, synthetic procedures, and detailed characterization (PDF)
