# Supplementary material for: Orthogonal Triple-Click Synthesis of Heteromimetic Triglycosylated Fullerenes
Source: Org Lett. 2026 Jul 17;28(30):9609–14. doi: 10.1021/acs.orglett.6c02245 (PMC13435311; doi:10.1021/acs.orglett.6c02245)
Supplement: Supplementary file 1 [file ol6c02245_si_001.pdf]

Supporting Information

Orthogonal Triple-Click Synthesis of Heteromimetic Tri-Glycosilated Fullerenes

Gema Nieto-Ortiz,[a] Jennifer Patino-Alonso,[a] Natalia Honrubia-Rodríguez,[a] Nazario Martín,*[a,b] M. Ángeles Herranz,*[a] Beatriz M. Illescas*[a]

[a] Departamento de Química Orgánica, Facultad de Ciencias Químicas, Universidad Complutense, E-28040 Madrid, Spain.

[b] IMDEA-Nanoscience, C/ Faraday 9, Campus de Cantoblanco, 28049 Madrid, Spain.

1. General Methods.. *S2*
2. Synthetic Procedures *S2*
3. References...*S37*

1. General Methods

Reagents and solvents were obtained from commercial suppliers and used without further purification. Compounds **3**[1], **8**[2], **11**[3], **14**[4], **17**[5] and **18**[6] were prepared according to previously reported procedures. All work-up and purification procedures were carried out with HPLC-grade solvents in air. Flash chromatography was performed using Scharlab silica gel (230-400 mesh, 0.040-0.063 mm) and for gel filtration Sephadex G-25 or G-50 were purchased from Cytiva. Analytical thin layer chromatography (TLC) was performed using E. Merck silica gel 60 F254 precoated plates (0.25 mm). The developed chromatogram was analyzed by UV lamp (λ = 254 nm) or charring with potassium permanganate as development reagent. ^{1}H and ^{13}C NMR spectra were performed on a Bruker AVIII 300MHz (^{1}H: 300 MHz, ^{13}C: 75 MHz) and Bruker AVIII 700MHz (^{1}H: 700 MHz, ^{13}C: 176 MHz) at 298 K using partially deuterated solvents as internal standards. Coupling constants (*J*) are expressed in Hertz (Hz) and chemical shifts (δ) in parts per million (ppm) relative to the solvent. Data are reported as follows: s = singlet, t = triplet, q = quartet, quint. = quintuplet, m = multiplet. DOSY NMR spectra were recorded on a Bruker AVIII 700MHz using D_2O as solvent at 306.1 K. The ledbpgp2s pulse sequence (longitudinal eddy-current delay with bipolar gradient pulses and 2 spoil gradients) was employed. Key acquisition parameters: relaxation delay (D1) = 2.0 s; diffusion delay (Δ) = 0.500 s; gradient pulse length (δ) = 900 µs; eddy current delay = 0.2 ms; smoothed square gradient shape (SMSQ10.32); gradient strength varied linearly from 2 to 100% of the maximum probe gradient over 16 increments; number of scans = 4.

FTIR spectra (cm^{-1}) were carried out in a Spectrum 3™ FT-IR spectrometer using a spectral range of 4000-400 cm^{-1}, with a resolution of 1 cm^{-1}. Mass analyses were carried out in a MALDI TOF/TOF Bruker Ultraflex using trans-2-[3-(4-tert-butylphenyl)-2-methyl-2-propenylidene]-malononitrile (DCTB) as matrix. Electrospray ionization (ESI) was performed in a Bruker IMPACT II spectrometer. The ionization conditions were the same for low-resolution as well as high-resolution experiments. The HR spectra were acquired at a resolution of 100,000.

UV-Vis absorption spectra were performed in a Shimadzu UV-3600 UV-VIS-NIR Spectrophotometer, using 1 cm pathlength quartz cells at 298 K.

2. Synthetic Procedures

Preparation of malonate 6

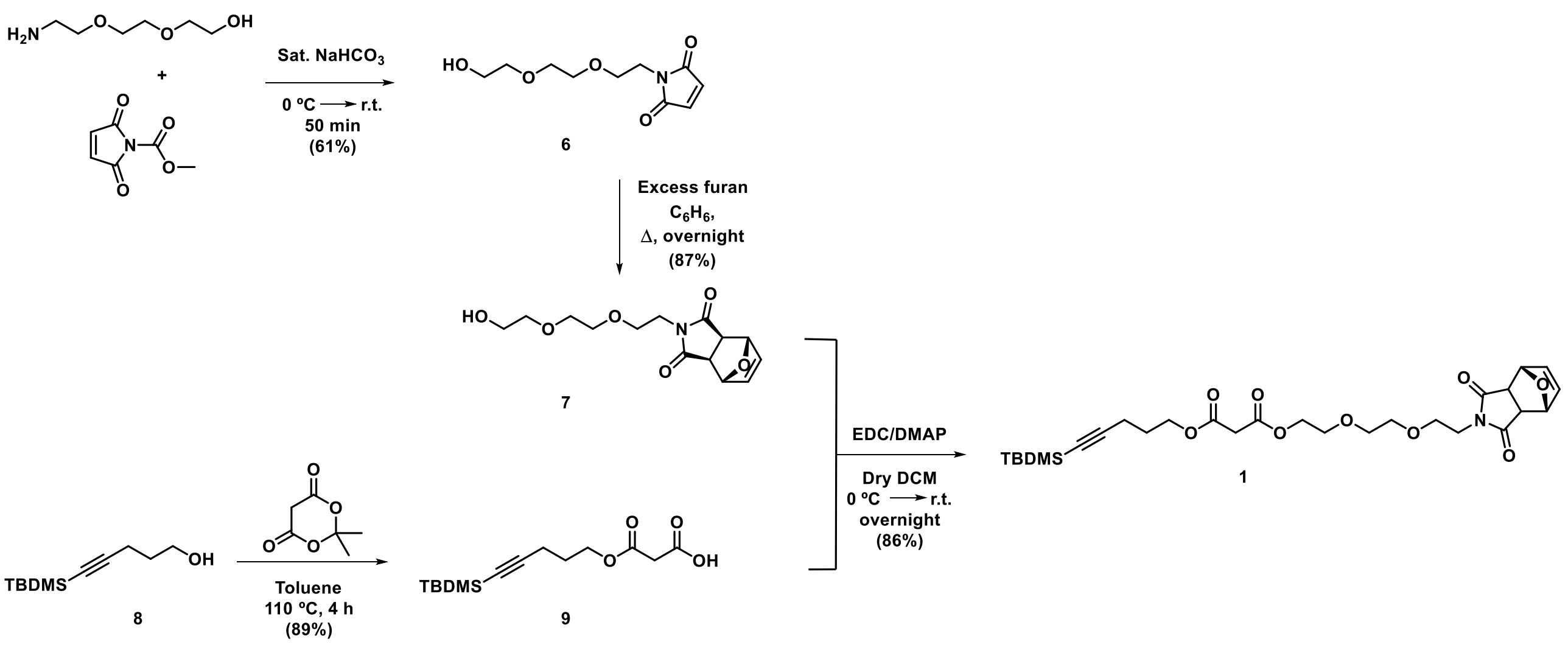

***Scheme S1**.* Synthetic pathway to obtain malonate **1**.

Compound 6

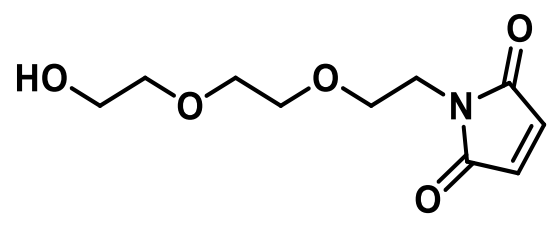

Methyl 2,5-dioxo-2,5-dihydro-1H-pyrrole-1-carboxylate (500 mg, 3.22 mmol, 1 eq) was added dropwise to a solution of 2-(2-(2-aminoethoxy)ethoxy)ethan-1-ol (480 mg, 3.22 mmol, 1 eq) in saturated $NaHCO_3$ (17 mL) at 0 ºC. The mixture was kept under stirring at this temperature for 30 minutes and then for 20 minutes at room temperature. The solution was extracted with $CHCl_3$ (3 x 20 mL) and washed with saturated NaCl (1 x 30 mL). The organic phase was dried over anh. $MgSO_4$, filtered and concentrated under reduced pressure. The crude was purified by column chromatography (silica gel, AcOEt), affording compound **6** (452 mg, 61%) as a colorless oil.

^{1}H NMR (300 MHz, $CDCl_3$) δ 6.69 (s, 2H, C<u>H</u>=C<u>H</u>), 3.76 – 3.55 (m, 12H, CH_2O, $N_{maleimide}$-C<u>H</u>$_2$), 2.48 (s, 1H, OH). **^{13}C NMR** (75 MHz, $CDCl_3$) δ 170.9 (CO), 134.3 (<u>C</u>H=<u>C</u>H), 72.5, 70.4, 70.1, 67.9 (<u>C</u>H$_2$-O), 61.9 (<u>C</u>H$_2$-OH), 37.2 ($N_{maleimide}$-<u>C</u>H$_2$).

FTIR-ATR (υ, cm^{-1}): 3452 (O-H), 3094 (=CH), 2919 (C-H), 1698 (C=O), 1408 (C-N), 1108 (C-O), 695 (=CH).

HRMS (ESI-TOF): *m/z* $[M+Na]^+$ calcd for $C_{10}H_{15}NO_5Na$: 252.0848; Found: 252.0838.

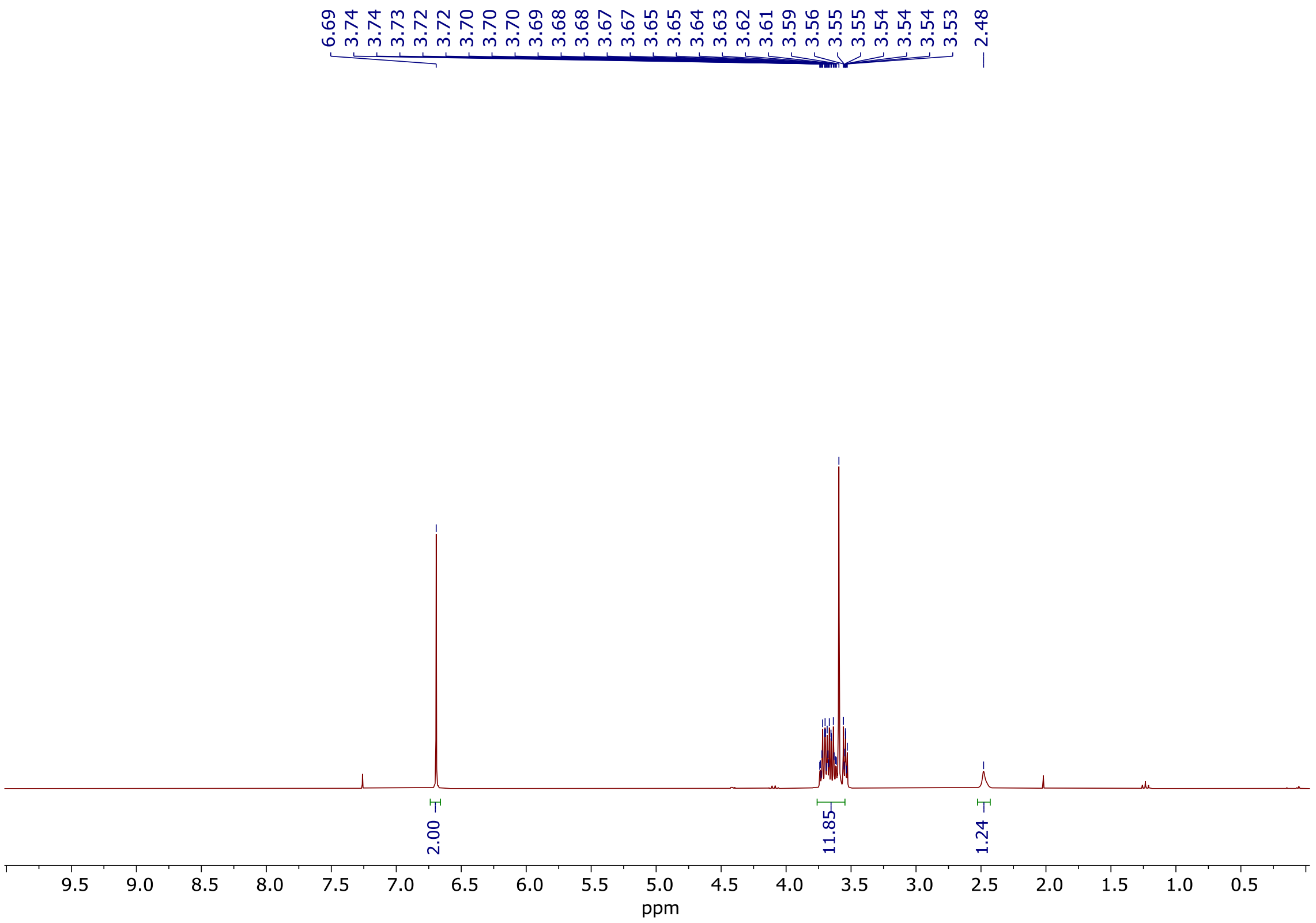

***Figure S1**. ^{1}H-NMR spectrum of compound **6** ($CDCl_3$, 300 MHz).*

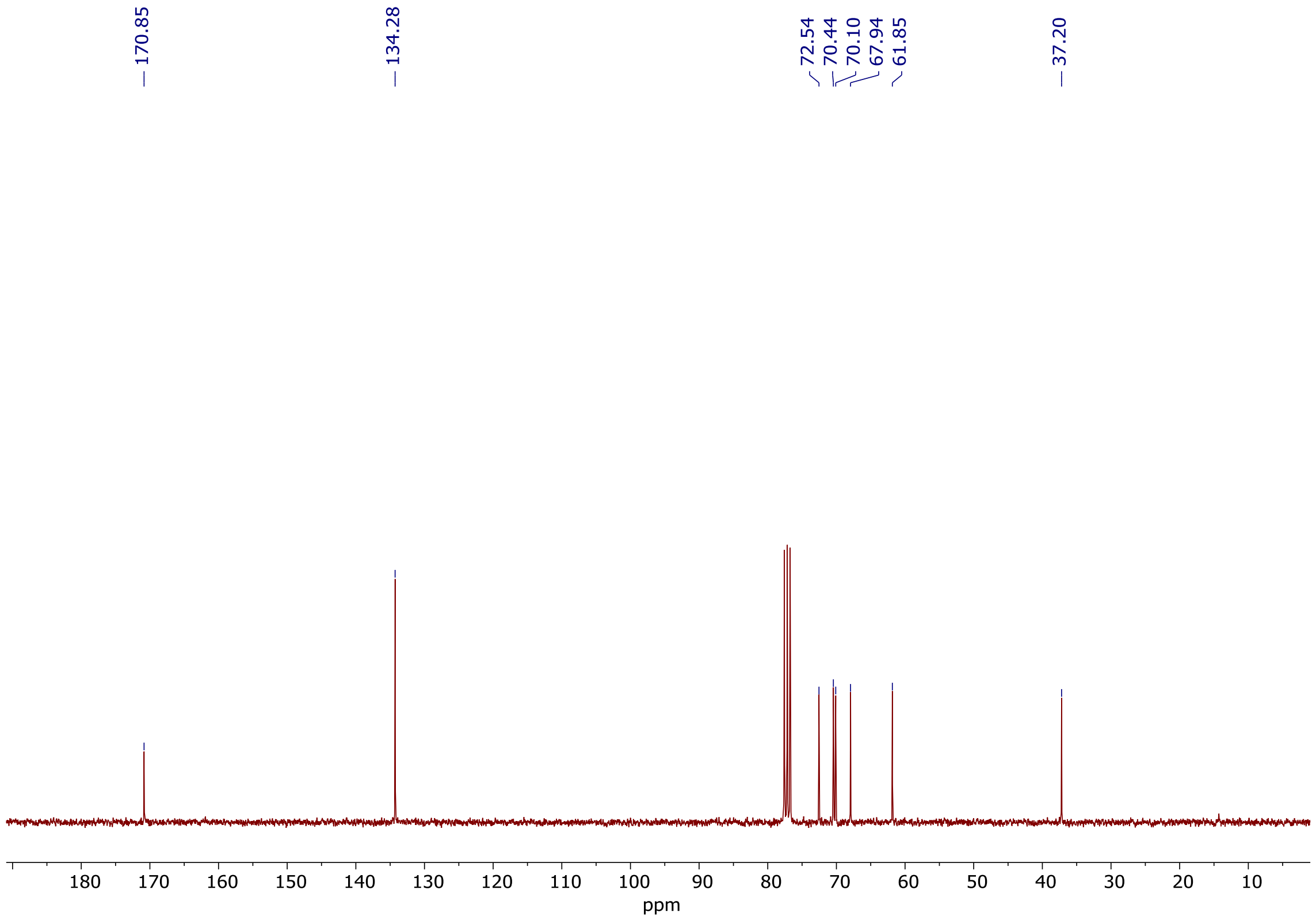

Figure S2*. ^{13}C-NMR spectrum of compound* ***6*** *($CDCl_3$, 75 MHz).*

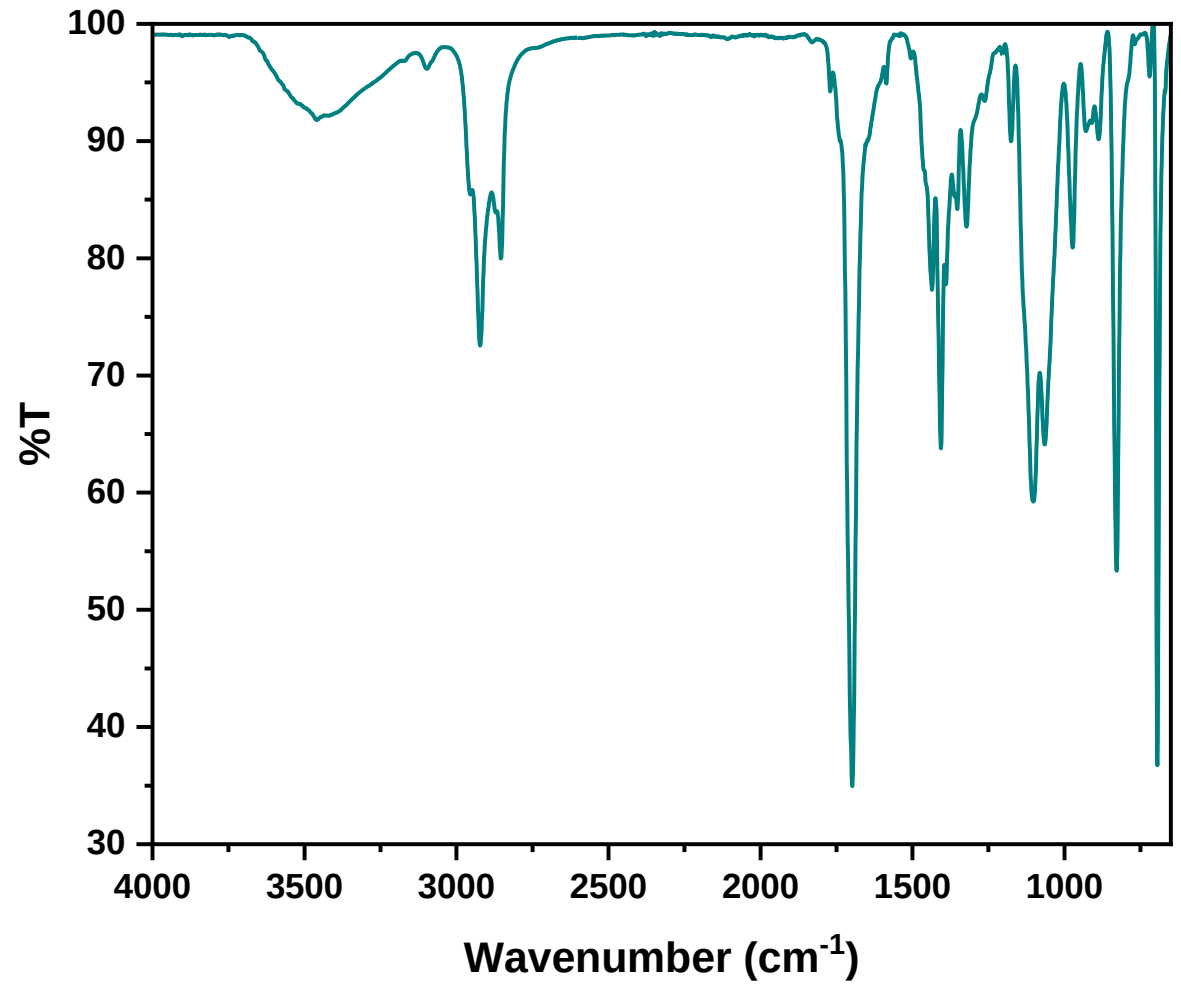

Figure S3. *FT-IR spectrum of compound* ***6****.*

Compound 7

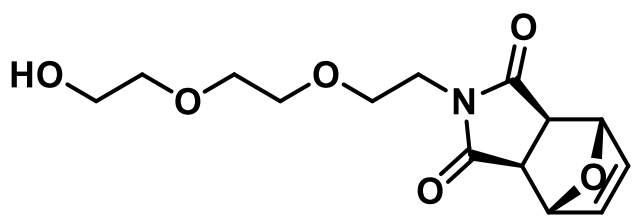

An excess of furan (3.3 mL, 45.56 mmol, 40 eq) was added to a solution of compound **6** (260 mg, 1.14 mmol, 1 eq) in benzene (30 mL) and the mixture was heated to reflux for 24 h using a sand bath. After that time, the solvent was removed under reduced pressure, and the obtained crude was purified by column chromatography (silica gel, AcOEt → Acetone) to afford compound **7** (295 mg, 87%) as a colorless oil.

^{1}H NMR (300 MHz, $CDCl_3$) δ 6.53 – 6.45 (m, 2H, C<u>H</u>=C<u>H</u>), 5.27 – 5.22 (m, 2H, 2xC$\underline{H}_{bridgehead}$), 3.70 – 3.51 (m, 12H, $N_{maleimide}$-C$\underline{H}_2$, C$\underline{H}_2$O), 2.87 – 2.81 (m, 2H, 2xNCOC<u>H</u>). **^{13}C NMR** (75 MHz, $CDCl_3$) δ 176.3 (CO), 136.6 (<u>C</u>H=<u>C</u>H), 80.9 (2x$\underline{C}H_{bridgehead}$), 72.5, 70.4, 70.1, 67.2 (<u>C</u>H_2-O) 61.8 (<u>C</u>H_2-OH), 47.5 (2xNCO<u>C</u>H), 38.3 ($N_{maleimide}$-<u>C</u>H_2).

FTIR-ATR (υ, cm^{-1}): 3461 (O-H), 2870 (C-H), 1690 (C=O), 1401 (C-N), 1124 (C-O).

HRMS (ESI-TOF): *m/z* $[M+Na]^+$ calcd for $C_{14}H_{19}NO_6Na$: 320.1110; Found: 320.1104.

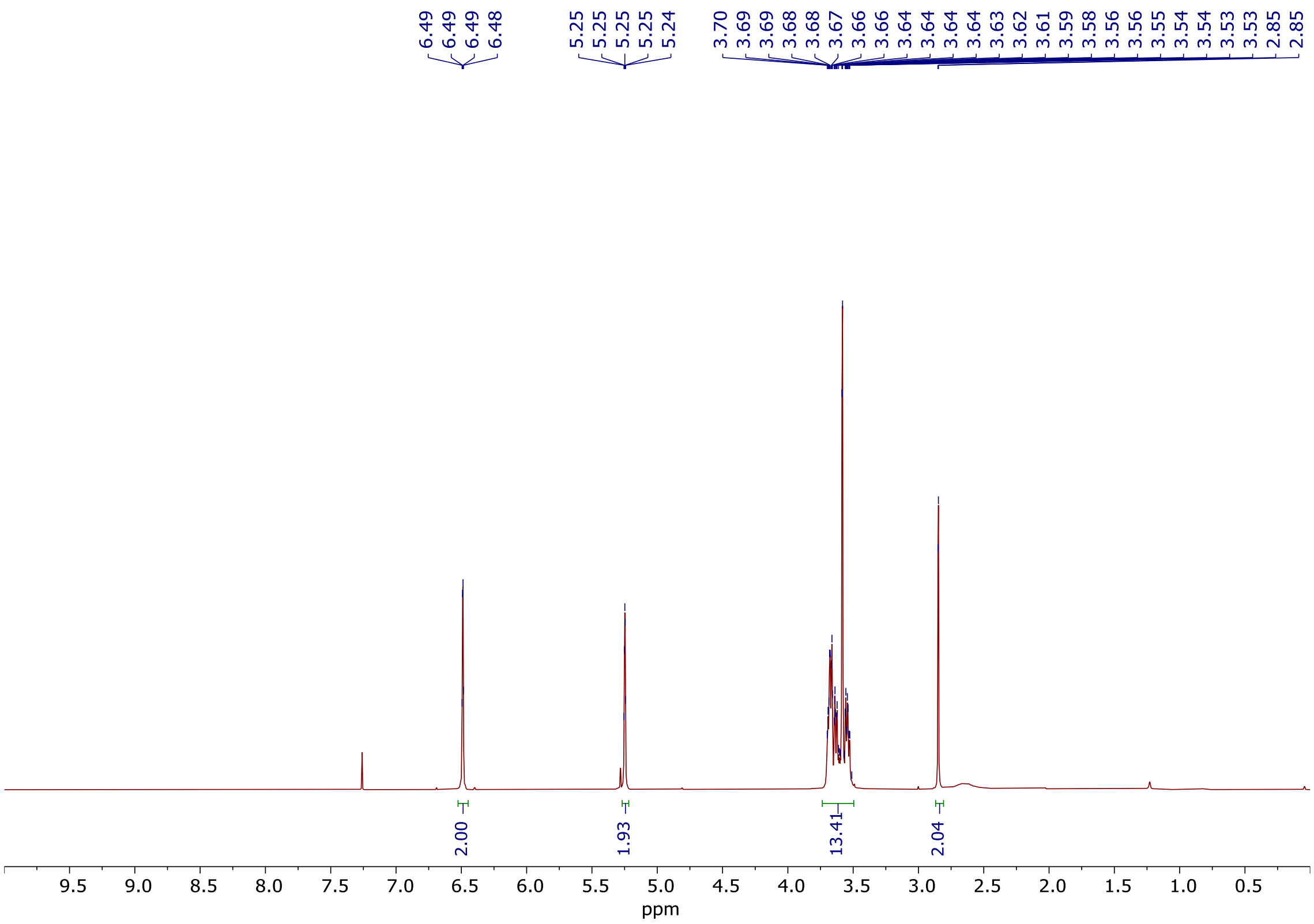

Figure S4. *^{1}H-NMR spectrum of compound **7** ($CDCl_3$, 300 MHz).*

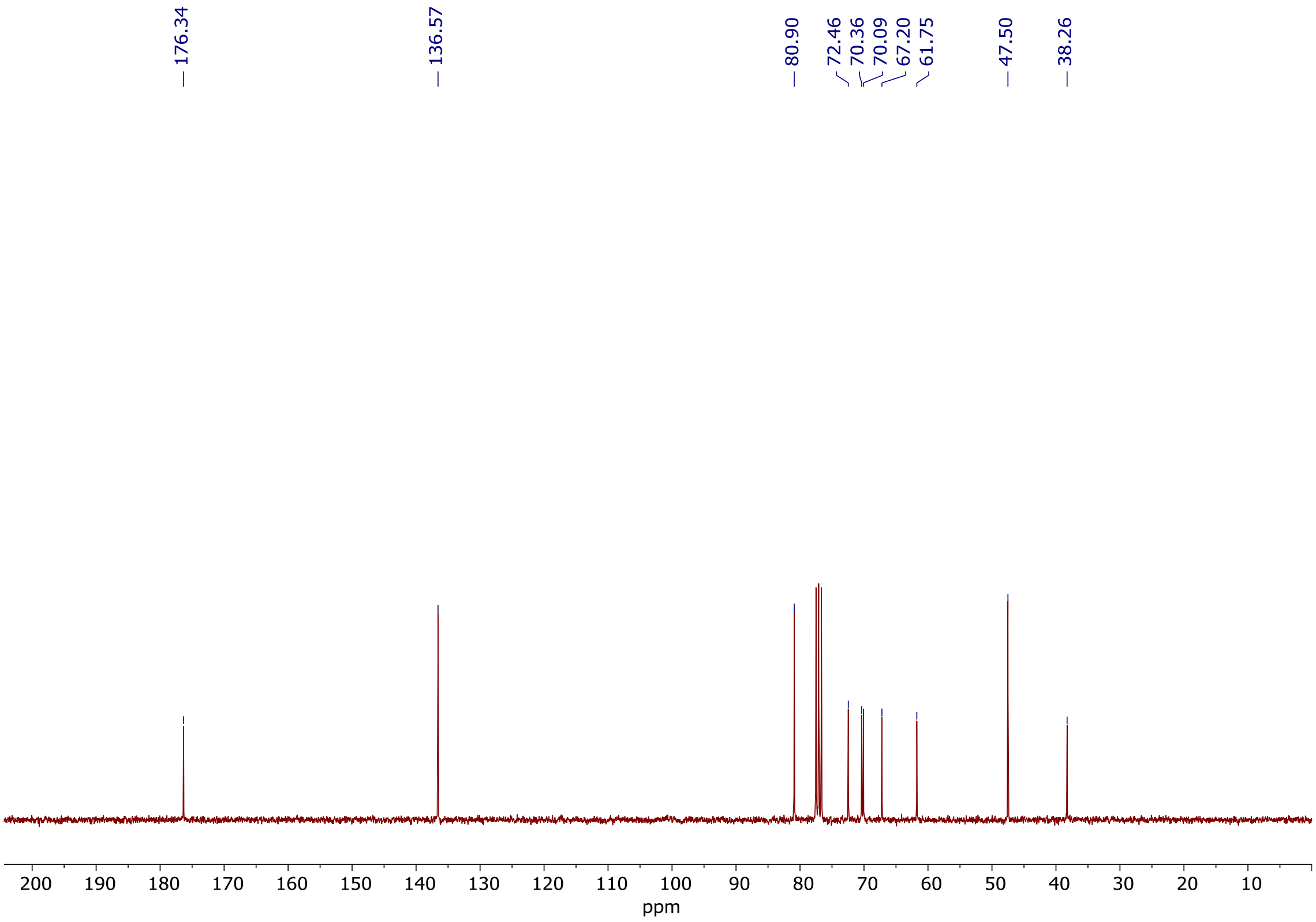

Figure S5. *^{13}C-NMR spectrum of compound **7** ($CDCl_3$, 75 MHz).*

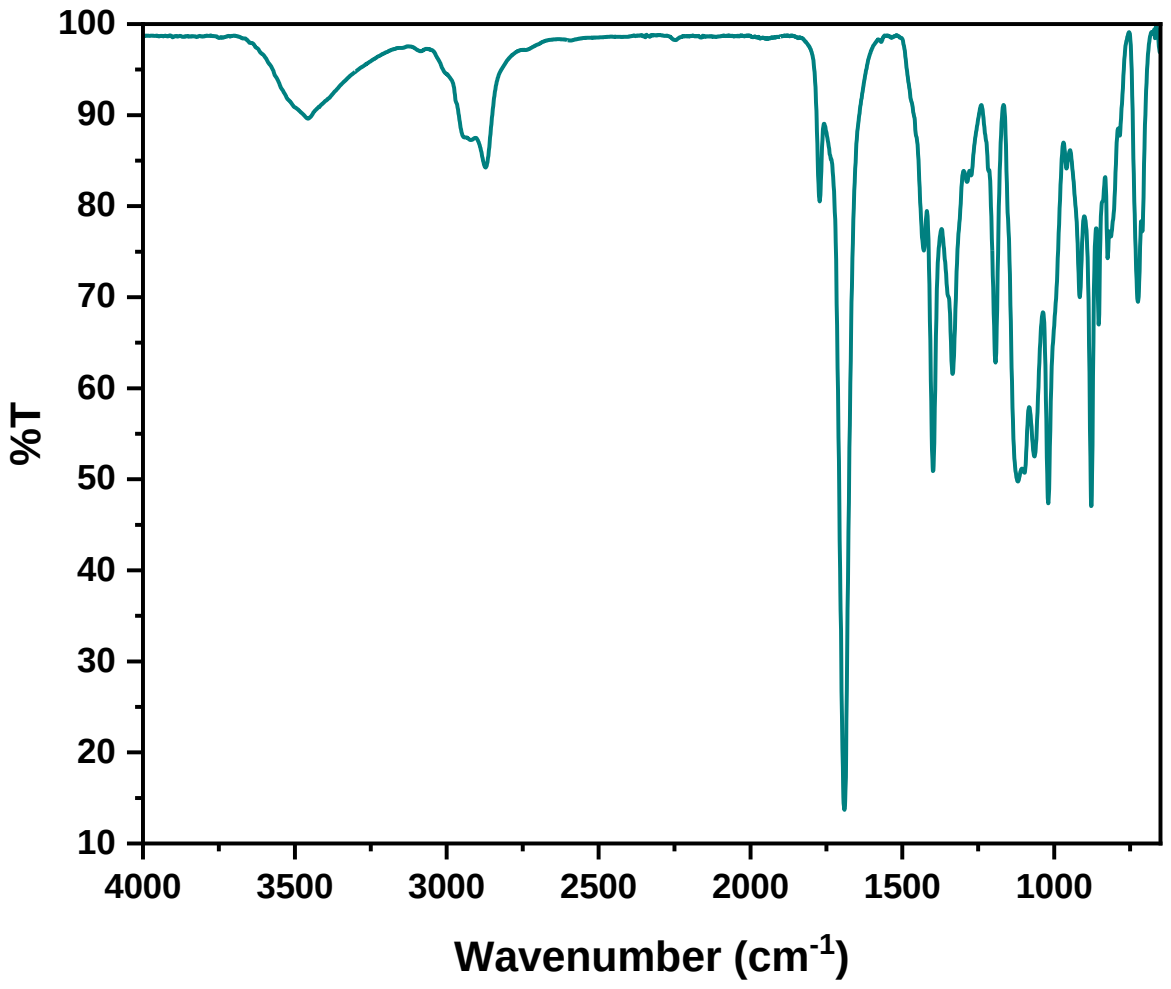

Figure S6. *FT-IR spectrum of compound **7**.*

Compound 9

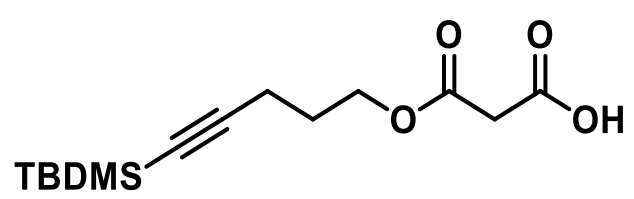

2,2-dimethyl-1,3-dioxane-4,6-dione/Meldrum's acid (420 mg, 2.94 mmol, 1 eq) was added to a solution of compound **8** (580 mg, 2.94 mmol, 1 eq) in toluene (15 mL) and was heated to reflux for 4 h using a sand bath. After that time, the solvent was removed under reduced pressure and the obtained crude was purified by column chromatography (silica gel, Hex:AcOEt 9:1 → Hex:AcOEt 1:1 → AcOEt) to afford compound **9** (734 mg, 89%) as a yellowish oil.

^{1}H NMR (300 MHz, $CDCl_3$) δ 4.21 (t, *J* = 6.4 Hz, 2H, C<u>H</u>$_2$O), 3.37 (s, 2H, COC<u>H</u>$_2$CO), 2.26 (t, *J* = 7.0 Hz, 2H, C≡CC<u>H</u>$_2$CH$_2$), 1.81 (quint, *J* = 6.7 Hz, 2H, CH$_2$C<u>H</u>$_2$CH$_2$), 0.84 (s, 9H, tBuSi), 0.00 (s, 6H, 2xC<u>H</u>$_3$Si). **^{13}C NMR** (75 MHz, $CDCl_3$) δ 171.1 (<u>C</u>O), 167.1 (<u>C</u>O), 105.9 (TBDMS-C≡<u>C</u>), 83.9 (TBDMS-<u>C</u>≡C), 64.7 (<u>C</u>H$_2$O), 40.7 (CO<u>C</u>H$_2$CO), 27.7 (CH$_2$<u>C</u>H$_2$CH$_2$), 26.2 (tBuSi), 16.6 ($C_{q,tBu}$), 16.6 (C<u>C</u>H$_2$CH$_2$), -4.37 (2x<u>C</u>H$_3$Si).

FTIR-ATR (υ, cm^{-1}): 3541 (O-H), 2931 (C-H), 2175 (C≡C), 1721 (C=O), 1157 (C-O).

HRMS (ESI-TOF): *m/z* [M+Na]$^+$ calcd for $C_{14}H_{24}O_4SiNa$: 307.1342; Found: 307.1332.

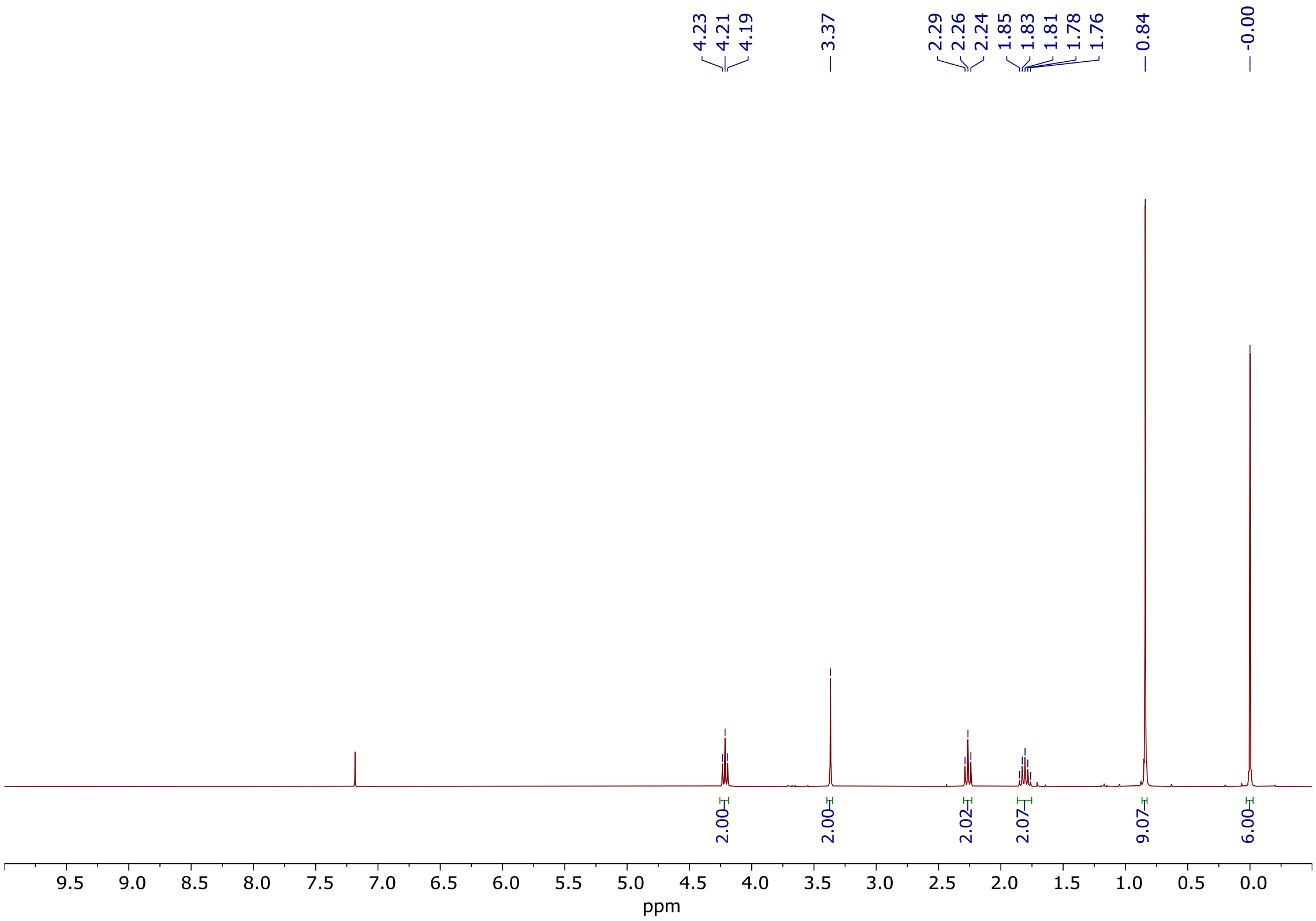

Figure S7. *^{1}H-NMR spectrum of compound **9** ($CDCl_3$, 300 MHz).*

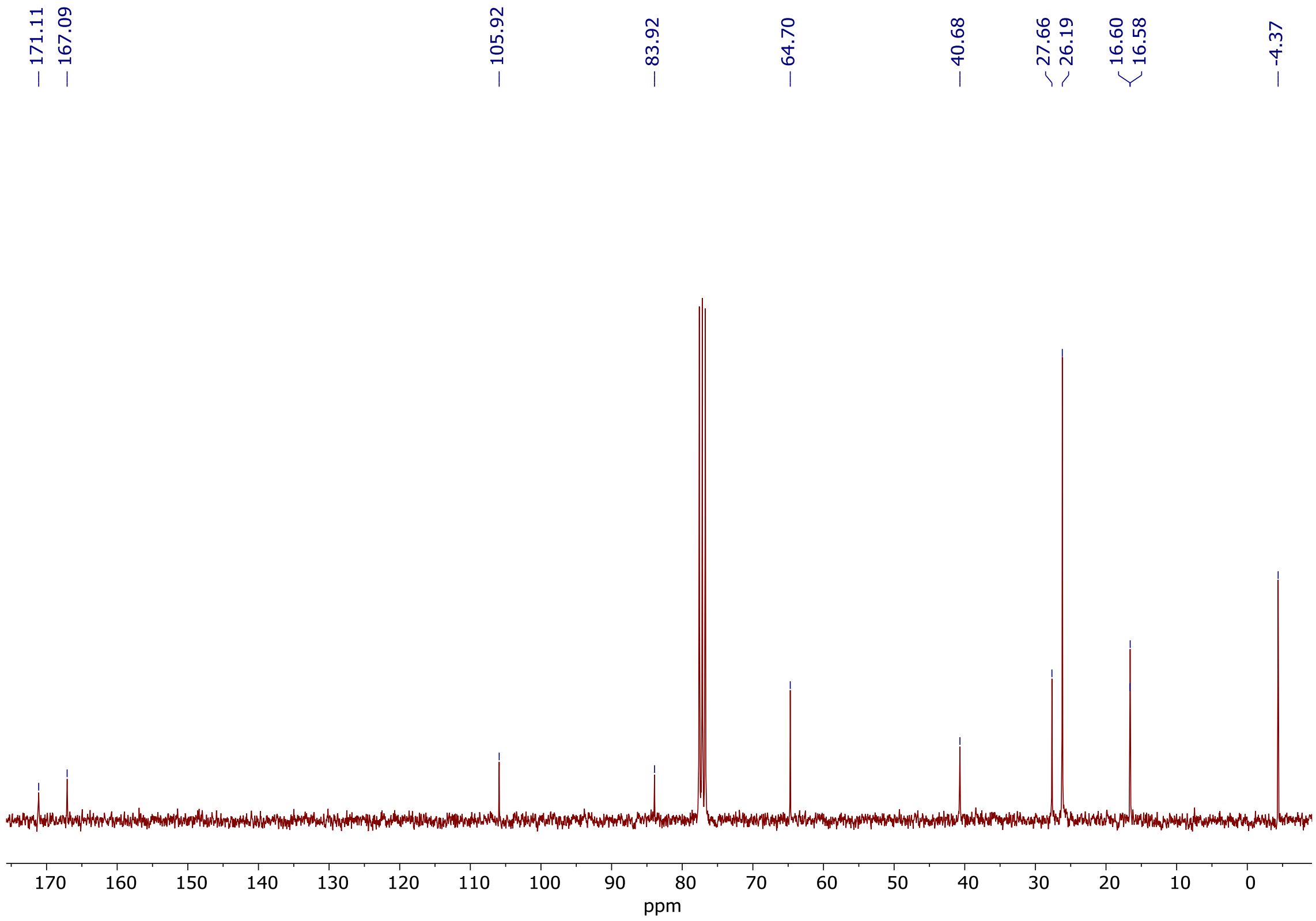

***Figure S8**. ^{13}C-NMR spectrum of compound **9** ($CDCl_3$, 75 MHz).*

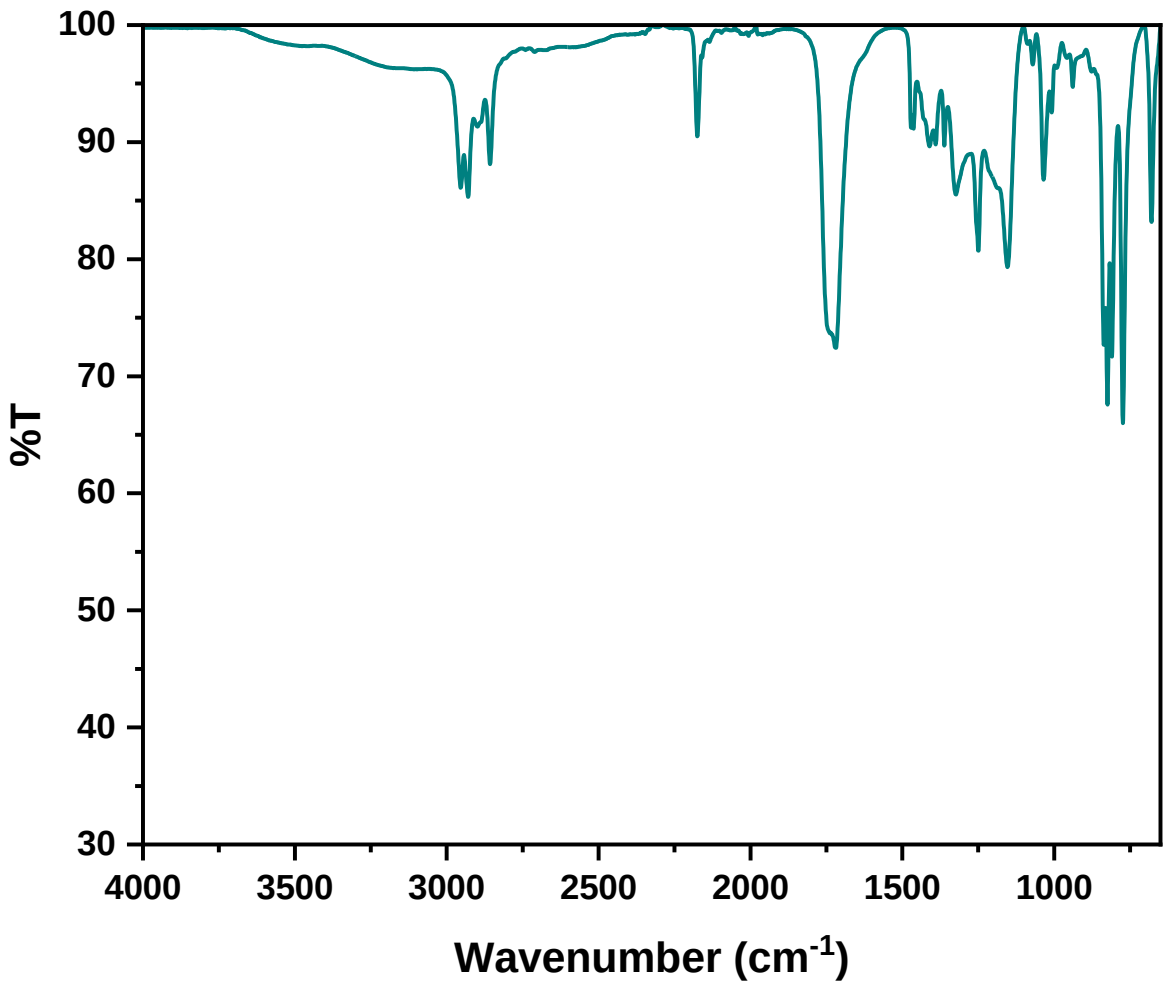

***Figure S9.** FT-IR spectrum of compound **9**.*

Compound 1

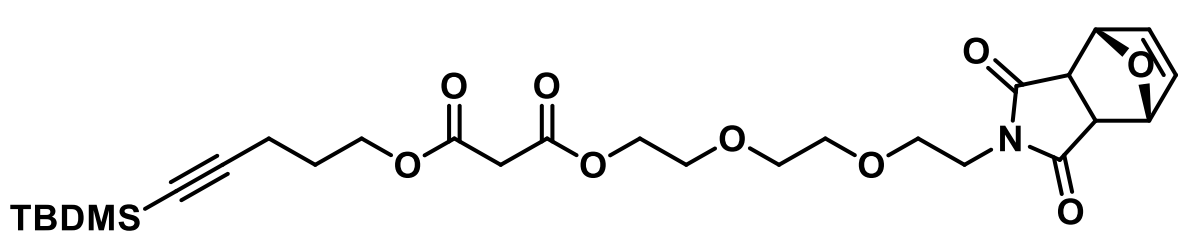

EDC·HCl (448 mg, 2.34 mmol, 2 eq) and DMAP (286 mg, 2.34 mmol, 2 eq) were added to a solution of compounds **7** (349 mg, 1.17 mmol, 1 eq) and **9** (334 mg, 1.17 mmol, 1 eq) in dry DCM (50 mL) at 0 °C under argon atmosphere. The mixture was kept under stirring at this temperature for 20 minutes and after that, for 48 h at room temperature. The reaction mixture was washed with deionized H_2O (2 x 50 mL) and NaCl (1 x 50 mL), and the organic phase was dried over anh. $MgSO_4$, filtered and

concentrated under reduced pressure. The crude was purified by column chromatography (silica gel, AcOEt), affording compound **1** (566 mg, 86%) as a yellowish oil.

^{1}H NMR (300 MHz, $CDCl_3$) δ 6.51 – 6.47 (m, 2H, C<u>H</u>=C<u>H</u>), 5.26 – 5.21 (m, 2H, 2xC$\underline{H}_{bridgehead}$), 4.30 – 4.20 (m, 4H, 2xC$\underline{H}_2$OCO), 3.69 – 3.56 (m, 10H, 4xC$\underline{H}_2$O, C$\underline{H}_2N_{maleimide}$), 3.40 (s, 2H, COC$\underline{H}_2$CO), 2.85 – 2.82 (m, 2H, 2xNCO<u>C</u>H), 2.31 (t, *J* = 7.1 Hz, 2H, C≡CC$\underline{H}_2CH_2$), 1.92 – 1.79 (quint., *J* = 7 Hz, 2H, $CH_2C\underline{H}_2CH_2$), 0.90 (s, 9H, tBuSi), 0.05 (s, 6H, 2xC$\underline{H}_3$Si). **^{13}C NMR** (75 MHz, $CDCl_3$) δ 176.2 (2x$CO_{maleimide}$), 166.7, 166.5 (2xCO), 136.7 (<u>C</u>H=<u>C</u>H), 106.1 (TBDMS-C≡<u>C</u>), 83.7 (TBDMS-<u>C</u>≡C), 81.0 (2xC$\underline{H}_{bridgehead}$), 70.7, 70.2, 68.9, 67.3 ($\underline{C}H_2$O), 64.7, 64.2 (2x$\underline{C}H_2$OCO), 47.6 (2xNCO<u>C</u>H), 41.5 (CO$\underline{C}H_2$CO), 38.3 ($N_{maleimide}$-$\underline{C}H_2$), 27.8 ($CH_2\underline{C}H_2CH_2$), 26.2 (tBuSi), 16.6 (C$\underline{C}H_2CH_2$), -4.4 (2x$\underline{C}H_3$Si).

FTIR-ATR (υ, cm^{-1}): 2941 (C-H), 2174 (C≡C), 1699 (C=O), 1336 (C-N), 1131 (C-O).

HRMS (ESI-TOF): *m/z* $[M+Na]^+$ calcd for $C_{28}H_{41}NO_9SiNa$: 586.2448; Found: 586.2445.

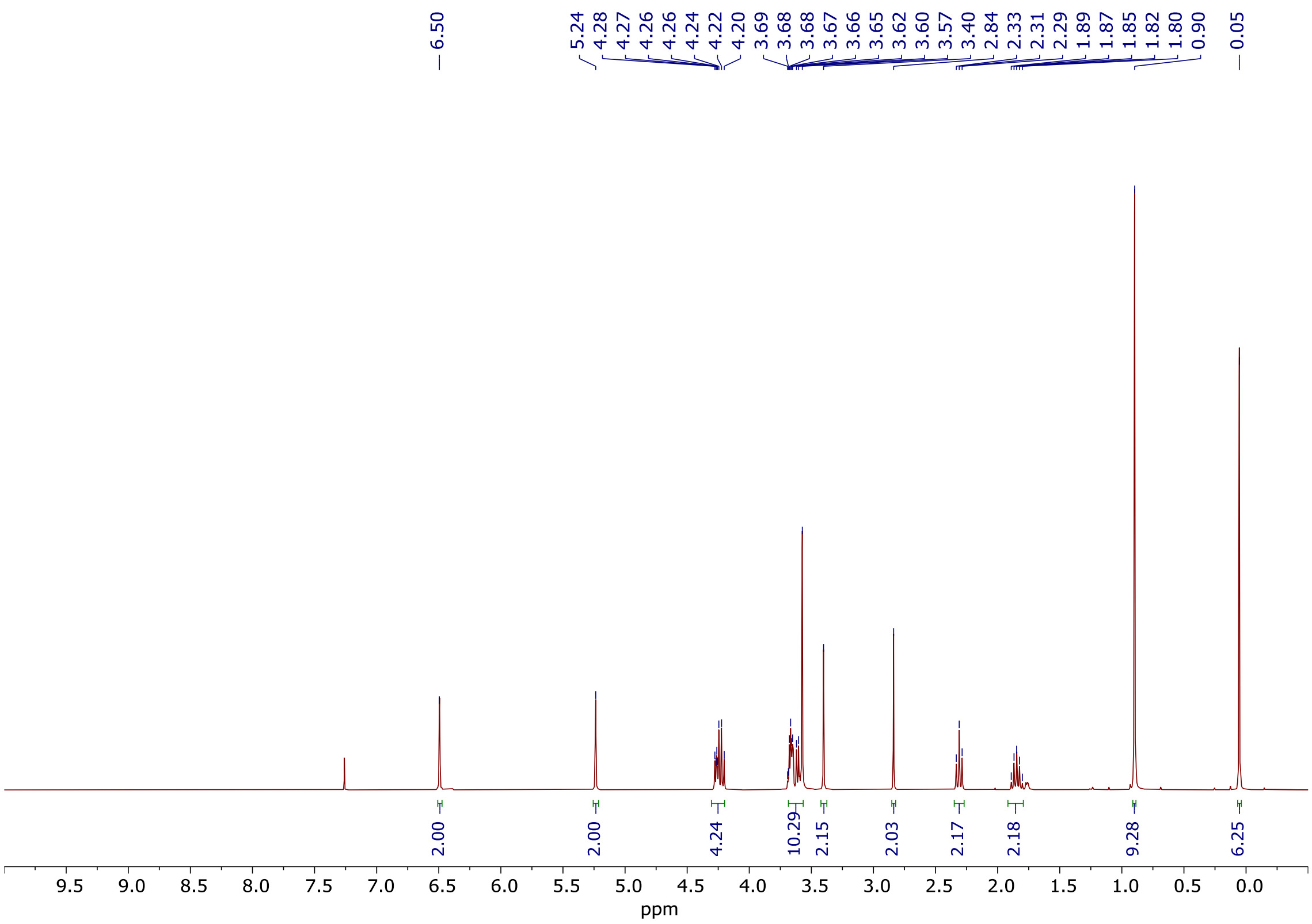

Figure S10. *^{1}H-NMR spectrum of compound **1** ($CDCl_3$, 300 MHz).*

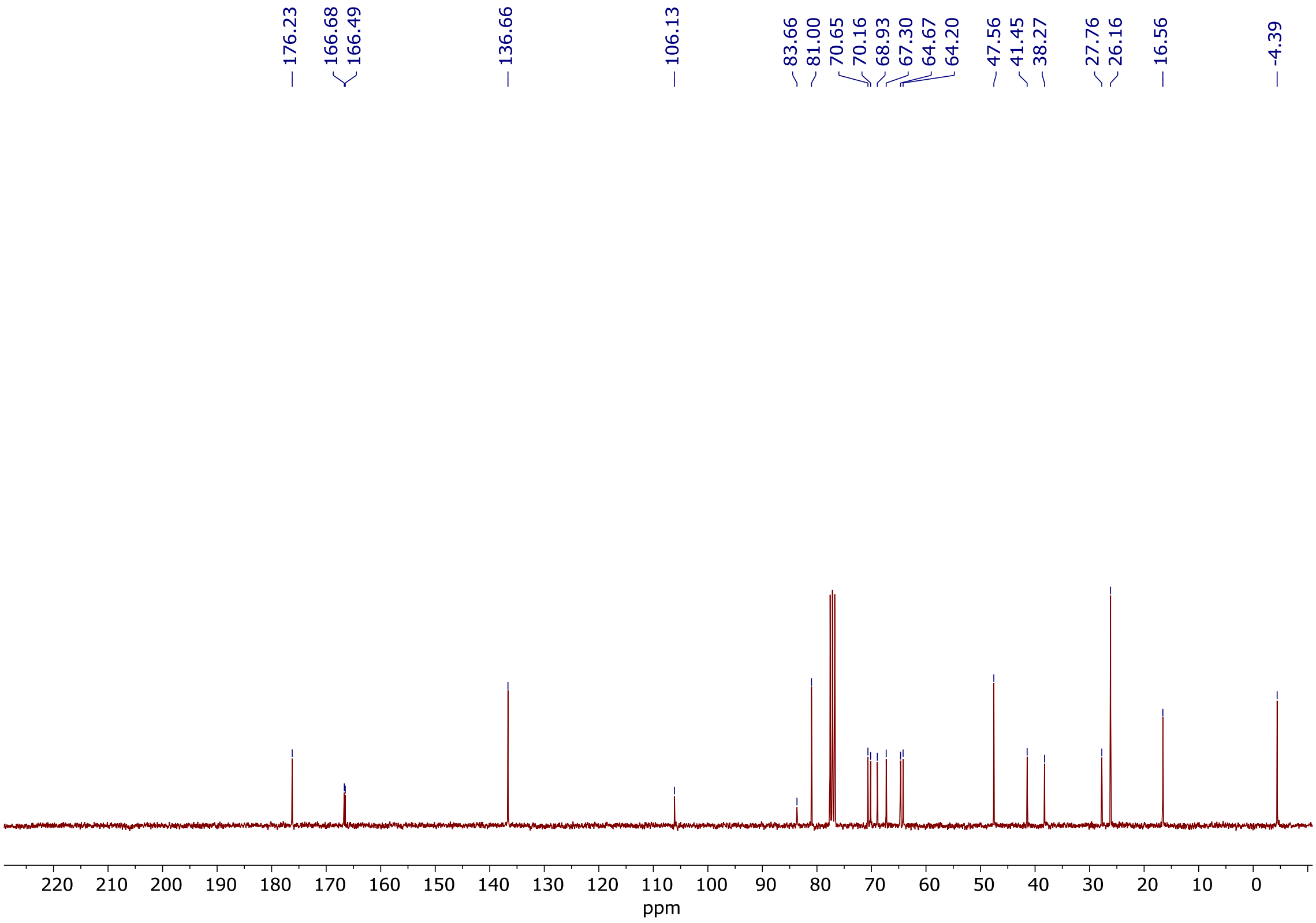

Figure S11*.* ^{13}*C-NMR spectrum of compound* ***1*** *(CDCl$_3$, 75 MHz).*

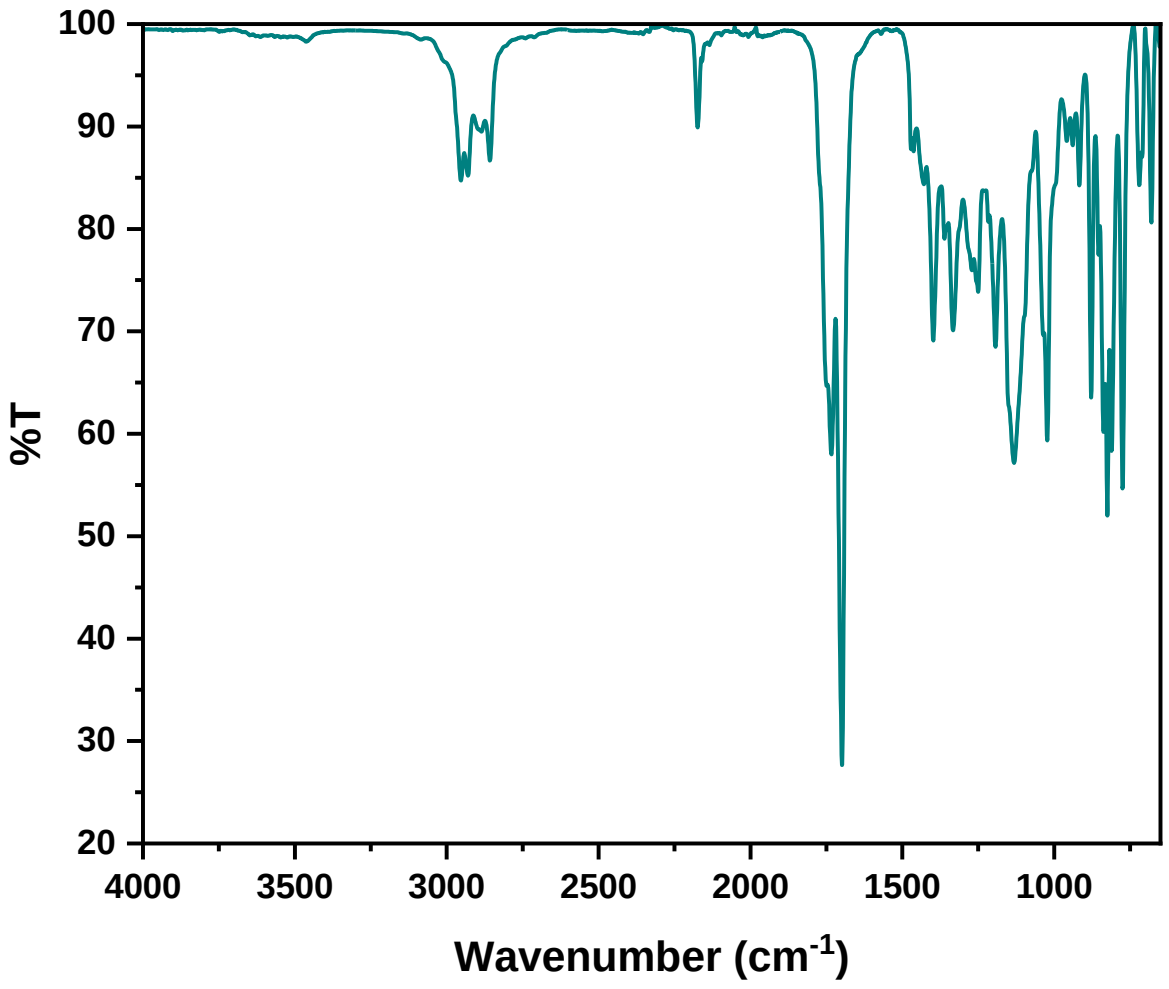

Figure S12. *FT-IR spectrum of compound* ***1****.*

Compound 2

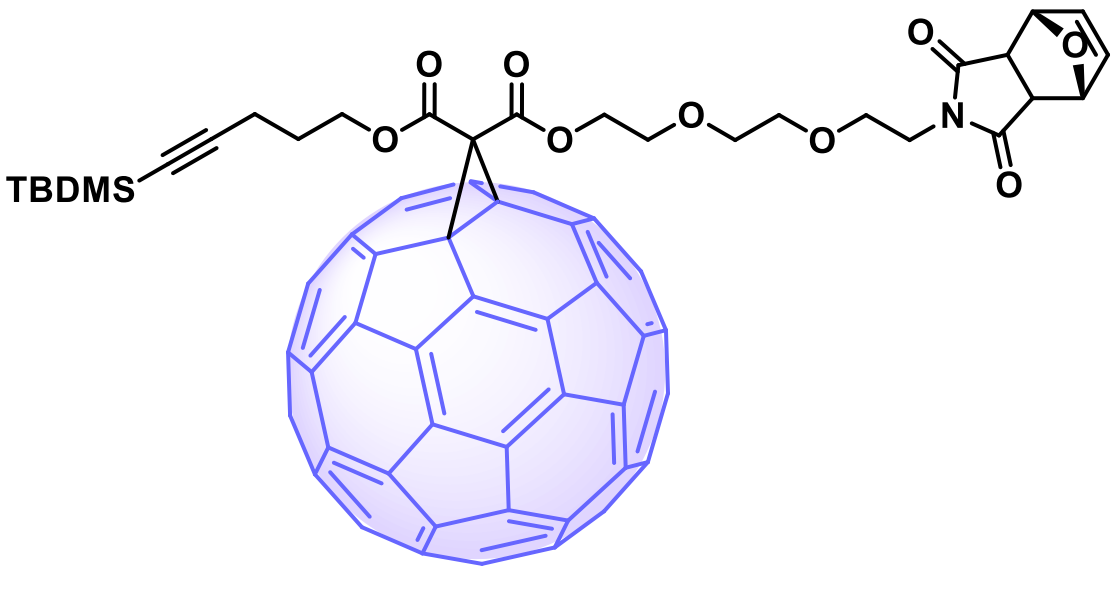

DBU (0.396 mL, 2.65 mmol, 2.5 eq) was added dropwise to a solution of compound **1** (597 mg, 1.06 mmol, 1 eq), fullerene C_{60} (1.53 g, 2.12 mmol, 2 eq) and I_2 (941 mg, 3.71 mmol, 3.5 eq) in dry toluene (500 mL) at 0 °C under argon atmosphere. The mixture was kept under stirring at this temperature for 3 h and then, a saturated aqueous solution of $Na_2S_2O_3$ (100 mL) was added to quench the reaction. The crude was extracted with toluene and washed with HCl 0.5 M (2 x 100 mL), saturated NaCl (1 x 100 mL) and H_2O (1 x 100 mL). The organic phase was dried over anh. $MgSO_4$, filtered and concentrated under reduced pressure. The obtained crude was purified by column chromatography (silica gel, CS_2 → Toluene:Acetone 15:1), affording compound **2** (721 mg, 53%) as a brown solid.

^{1}H NMR (300 MHz, $CDCl_3$) δ 6.52 – 6.48 (m, 2H, C<u>H</u>=C<u>H</u>), 5.26 – 5.24 (m, 2H, 2xC<u>H</u>$_{bridgehead}$), 4.66 – 4.57 (m, 4H, C<u>H</u>$_2$OCO), 3.87 – 3.81 (m, 2H, C<u>H</u>$_2$N$_{maleimide}$), 3.71 – 3.58 (m, 8H, 4xC<u>H</u>$_2$O), 2.86 – 2.84 (m, 2H, 2xNCO<u>C</u>H), 2.44 (t, J = 7.0 Hz, 2H, C≡CC<u>H</u>$_2$CH$_2$), 2.06 (quint., J = 6.6 Hz, 2H, CH$_2$C<u>H</u>$_2$CH$_2$), 0.93 (s, 9H, tBuSi), 0.09 (s, 6H, 2xC<u>H</u>$_3$Si). **^{13}C NMR** (75 MHz, $CDCl_3$) δ 176.23 (CO$_{maleimide}$), 163.73, 163.53 (CO), 145.40, 145.37, 145.32, 145.25, 145.03, 144.82, 144.75, 144.02, 143.23, 143.15, 143.12, 142.34, 142.04, 141.99, 141.11, 141.05, 139.19, 139.16 (C$_{sp2,fullerene}$), 136.69 (<u>C</u>H=<u>C</u>H), 105.87 (TBDMS-C≡<u>C</u>), 84.14 (TBDMS-<u>C</u>≡C), 81.03 (2xC<u>H</u>$_{bridgehead}$), 71.60 (C$_{sp3}$), 70.80, 70.26, 68.89, 67.38 (CH$_2$O), 66.37, 66.03 (2x<u>C</u>H$_2$OCO), 52.22 (C$_q$), 47.61 (2xNCO<u>C</u>H), 38.30 (N$_{maleimide}$-<u>C</u>H$_2$), 27.90 (CH$_2$<u>C</u>H$_2$CH$_2$), 26.25 (tBuSi), 16.74 (C$_{q,tBu}$), 16.65 (C<u>C</u>H$_2$CH$_2$), -4.30 (2x<u>C</u>H$_3$Si).

FTIR-ATR (υ, cm^{-1}): 2941 (C-H), 2174 (C≡C), 1699 (C=O), 1336 (C-N), 1131 (C-O).

HRMS (MALDI-ToF): *m/z* [M]$^{•+}$ calcd for $C_{88}H_{39}NO_9Si$: 1281.2394; Found: 1281.2384.

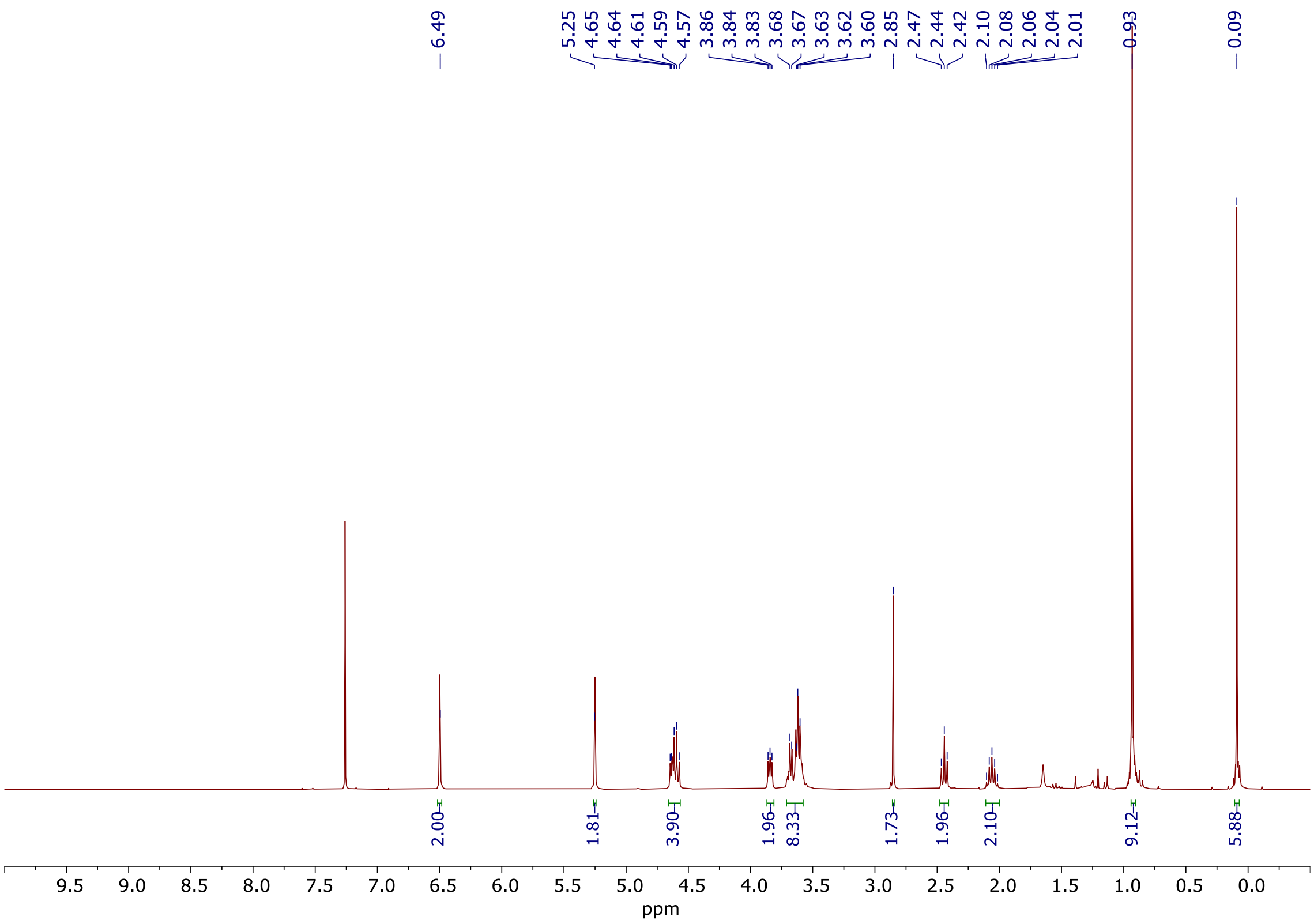

***Figure S13.** ^{1}H-NMR spectrum of compound **2** ($CDCl_3$, 300 MHz).*

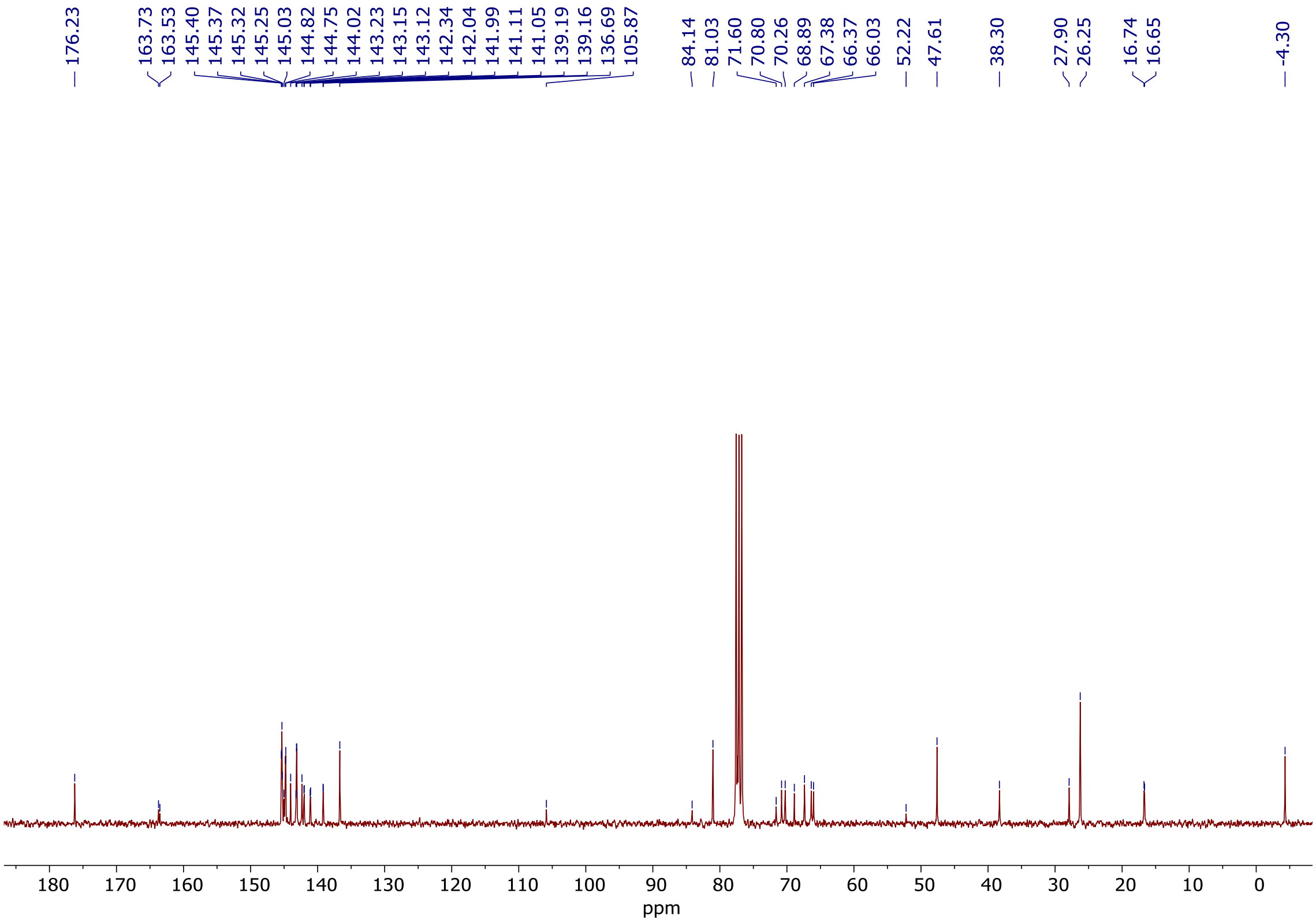

Figure S14. *^{13}C-NMR spectrum of compound **2** ($CDCl_3$, 75 MHz).*

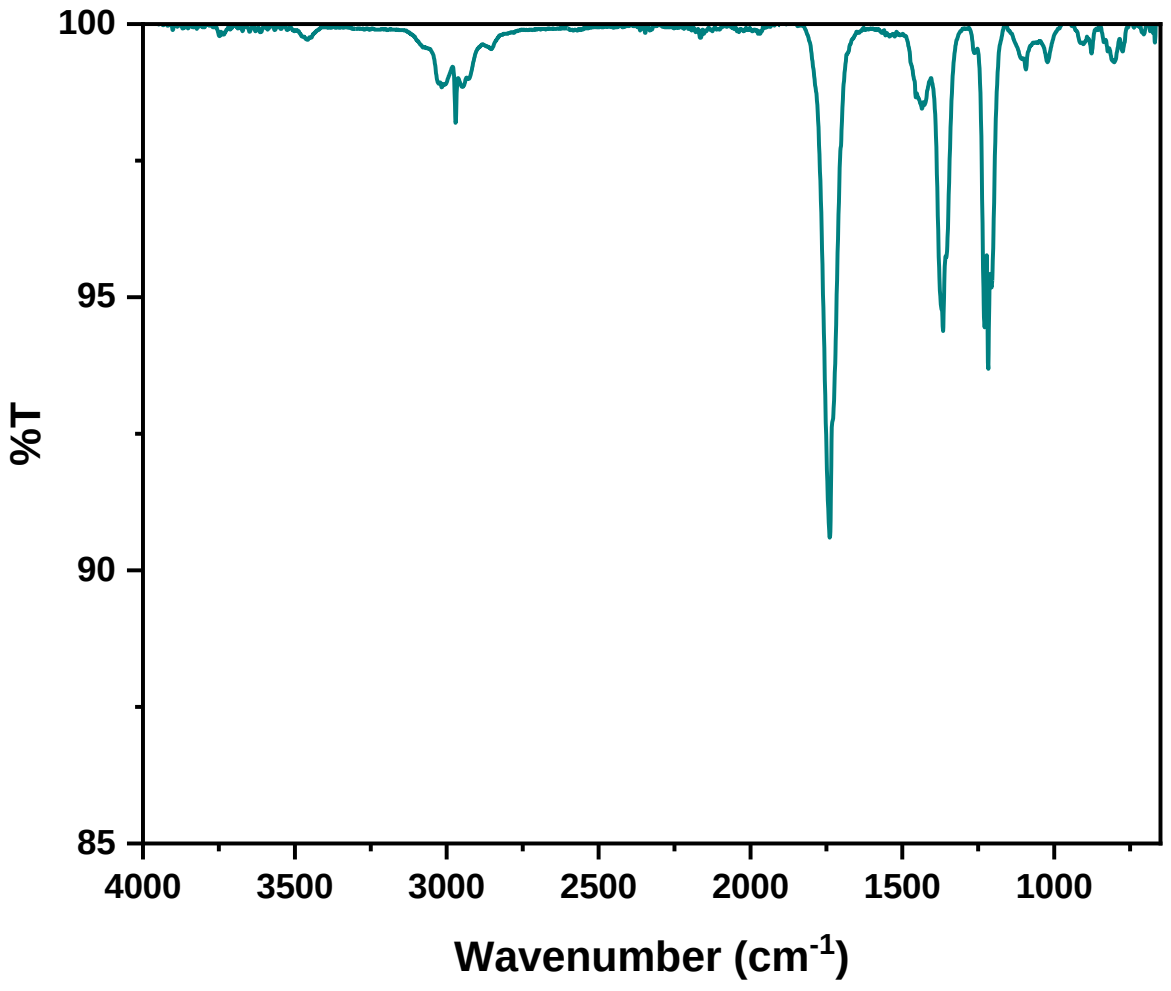

Figure S15. *FT-IR spectrum of compound **2**.*

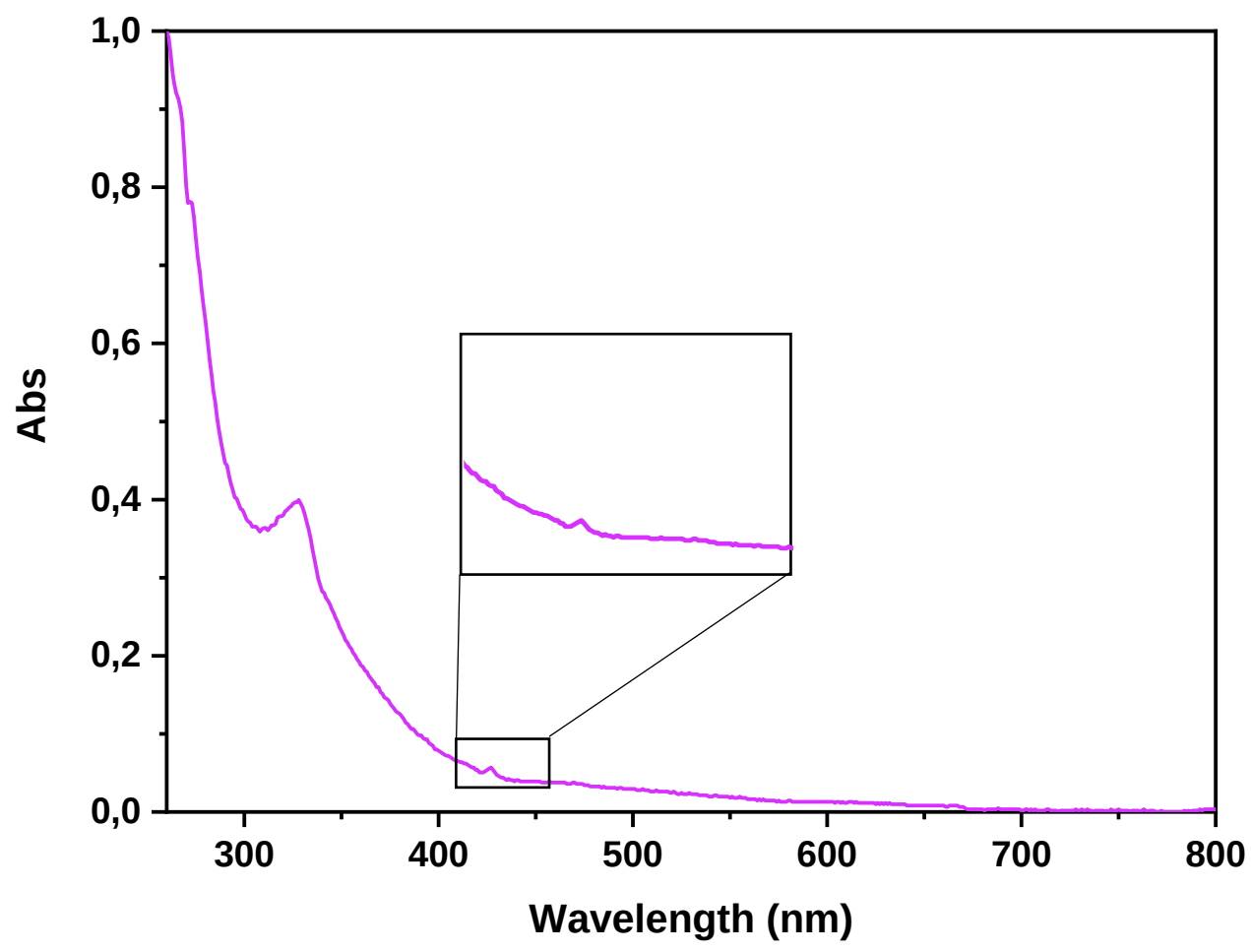

***Figure S16**. UV-Vis spectrum of compound **2** ($CHCl_3$, 10^{-5} M).*

Compound 4

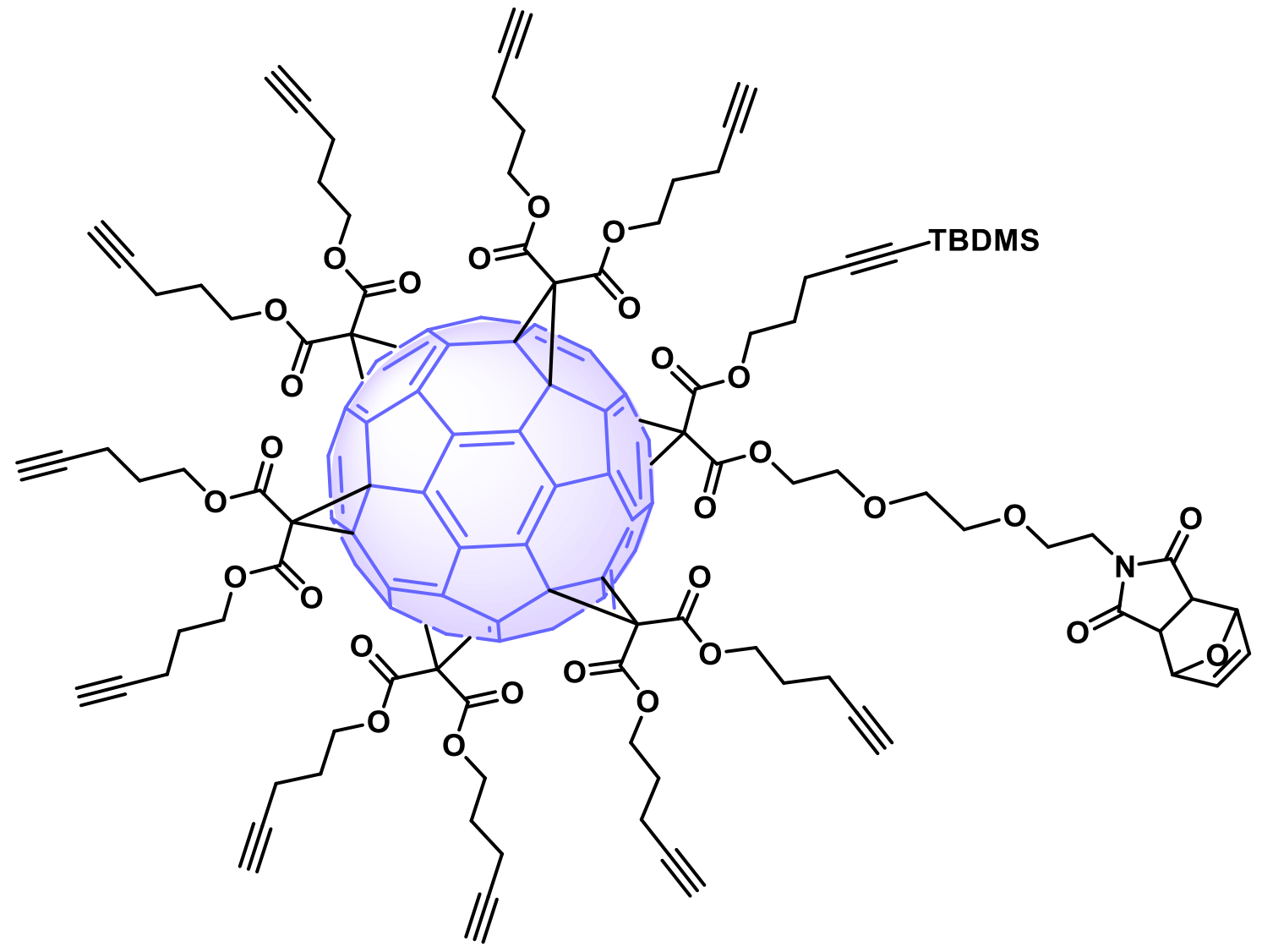

Compound **3** (1.29 g, 5.46 mmol, 10 eq) was added to a solution of compound **2** (0.70 g, 0.55 mmol, 1 eq) and CBr_4 (14.48 g, 43.68 mmol, 80 eq) in dry toluene (160 mL) under argon atmosphere. Then, DBU (1.63 mL, 10.92 mmol, 20 eq) was added dropwise to the mixture and it was kept under stirring at room temperature for 72 hours. After that time, a saturated aqueous solution of $Na_2S_2O_3$ (100 mL) was added to quench the reaction. The crude was extracted with toluene and washed with HCl 1 M (2 x 100 mL), saturated NaCl (1 x 100 mL) and H_2O (1 x 100 mL). The organic phase was dried over anh. $MgSO_4$, filtered and concentrated under reduced pressure. The obtained crude was purified by column chromatography (silica gel, AcOEt:Hexane 1:1→ AcOEt:Hexane 2:1), affording compound **4** (962 mg, 72%) as a dark red solid.

^{1}H NMR (700 MHz, $CDCl_3$) δ 6.55 – 6.43 (m, 2H, C<u>H</u>=C<u>H</u>), 5.27 – 5.18 (m, 2H, 2xC<u>H</u>$_{bridgehead}$), 4.60 – 4.11 (m, 24H, 12xC<u>H</u>$_2$OCO), 3.74 – 3.48 (m, 10H, C<u>H</u>$_2$N$_{maleimide}$, 4xC<u>H</u>$_2$O), 2.89 – 2.78 (m, 2H, 2xNCO<u>C</u>H), 2.47 – 2.17 (m, 22H, 11xC≡CC<u>H</u>$_2$CH$_2$), 2.13 – 1.95 (m, 10H, 10xC≡C<u>H</u>), 1.97 – 1.77 (m, 22H, 11xCH$_2$C<u>H</u>$_2$CH$_2$), 0.91 (s, 9H, tBuSi), 0.09 – 0.03 (s, 6H, 2xC<u>H</u>$_3$Si). **^{13}C NMR** (176 MHz, $CDCl_3$) δ 176.2 (CO$_{maleimide}$), 163.8 (CO), 145.9, 141.3 (C$_{sp2,fullerene}$), 136.7 (<u>C</u>H=<u>C</u>H), 106.1 (TBDMS-C≡<u>C</u>), 83.8 (TBDMS-<u>C</u>≡C), 82.5 (<u>C</u>≡CH), 81.0 (C<u>H</u>$_{bridgehead}$), 78.5 (C≡<u>C</u>H), 70.7, 70.1 (CH$_2$O), 69.9 (C≡<u>C</u>H), 69.1 (C$_{sp3,fullerene}$), 68.6, 67.3 (CH$_2$O), 66.0, 65.8, 65.5 (<u>C</u>H$_2$OCO), 47.6 (NCO<u>C</u>H), 45.2 (C$_q$), 39.5 (C$_q$) , 38.2 (N$_{maleimide}$-<u>C</u>H$_2$), 27.7 (TBDMS-C≡CCH$_2$<u>C</u>H$_2$), 27.2, 27.1 (HC≡CCH$_2$<u>C</u>H$_2$), 26.2 (tBuSi), 16.6 (C$_{q,tBu}$), 16.5 (TBDMS-C≡C<u>C</u>H$_2$), 15.2 (HC≡C<u>C</u>H$_2$CH$_2$), -4.4 (<u>C</u>H$_3$Si).

FTIR-ATR (υ, cm^{-1}): 3279 (≡C-H), 2931, (C-H), 2163 (C≡C), 1736 (C=O), 1398 (C-N), 1202 (C-O).

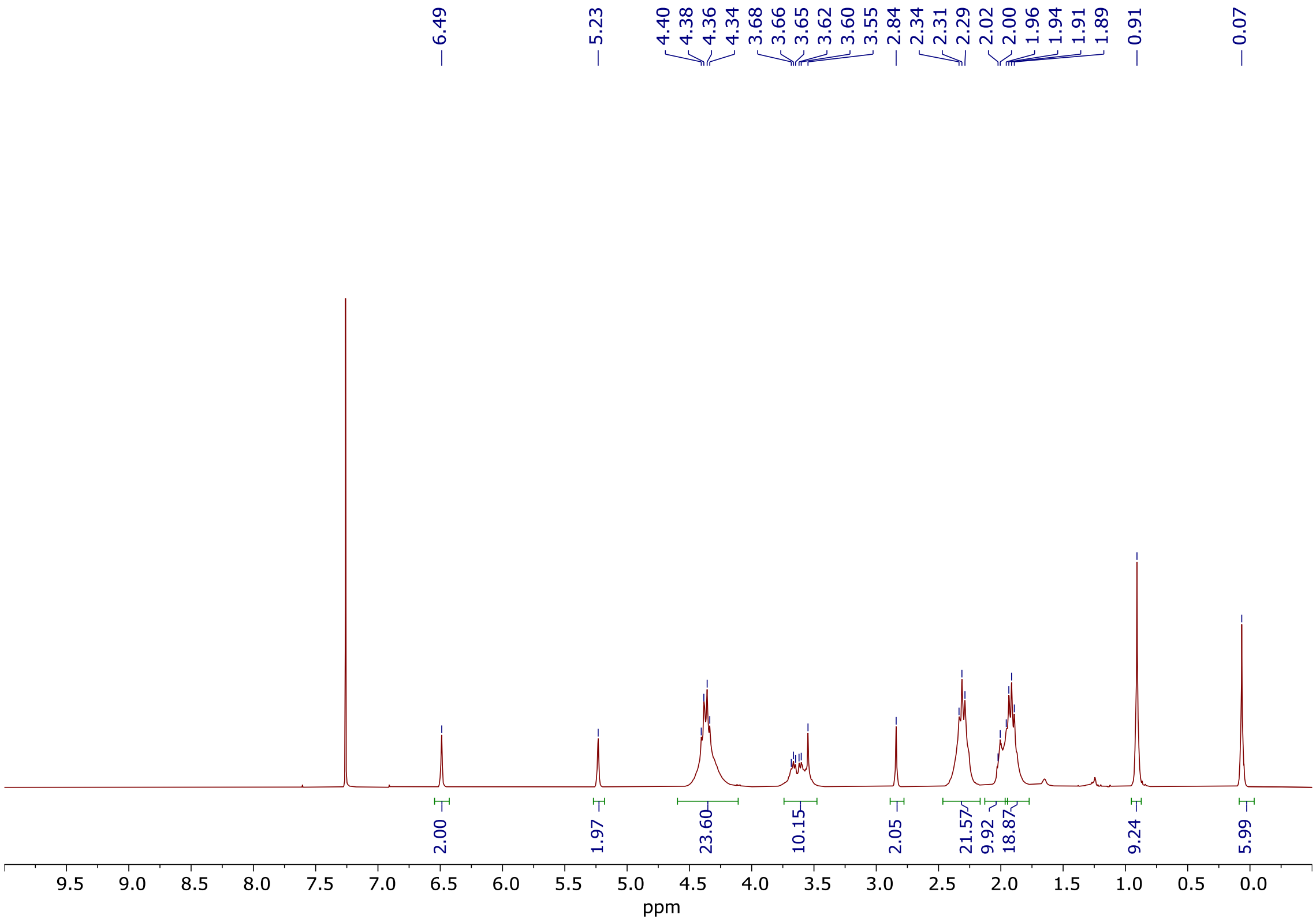

Figure S17. *^{1}H-NMR spectrum of compound **4** ($CDCl_3$, 700 MHz).*

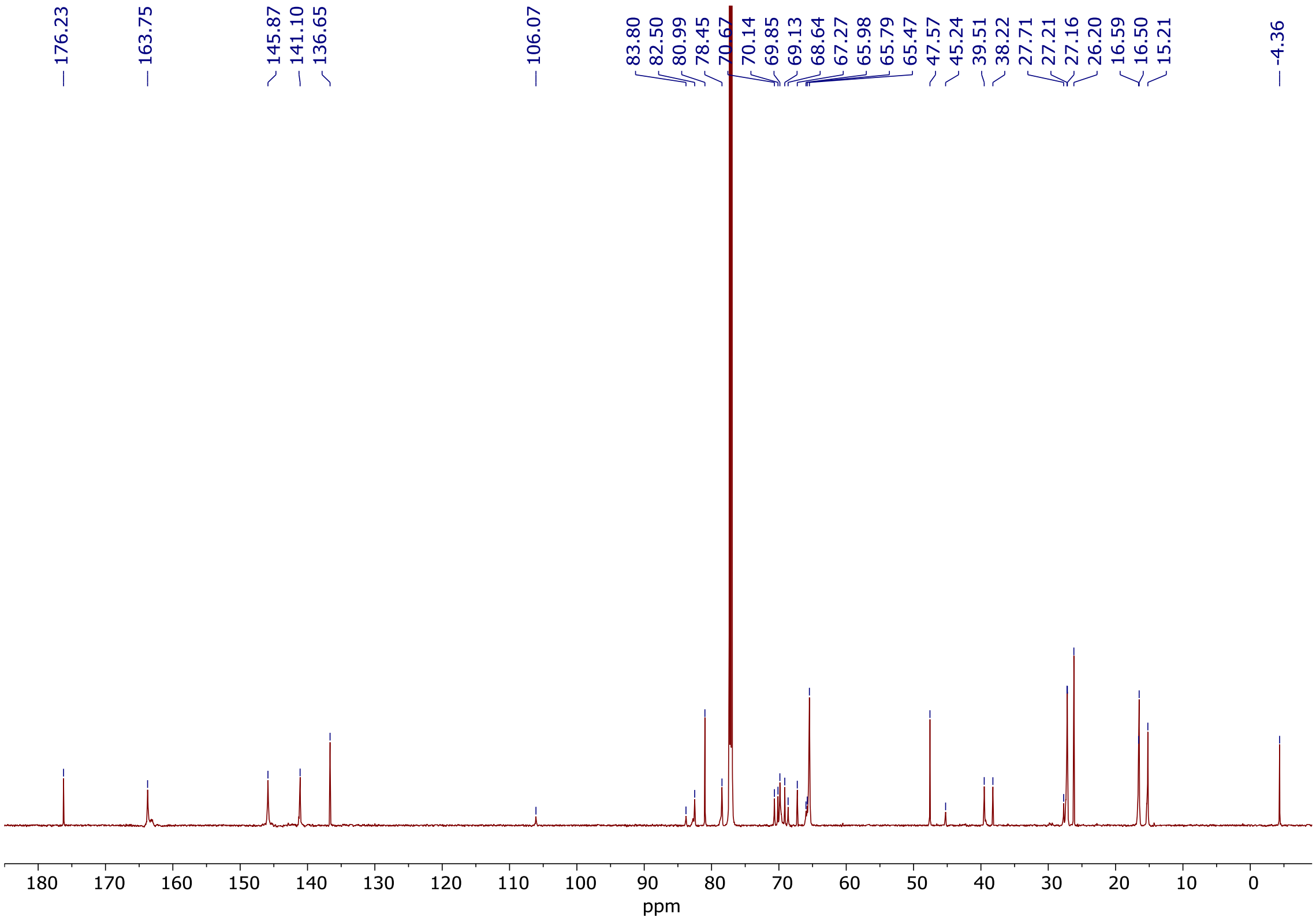

Figure S18. *^{13}C-NMR spectrum of compound **4** ($CDCl_3$, 176 MHz).*

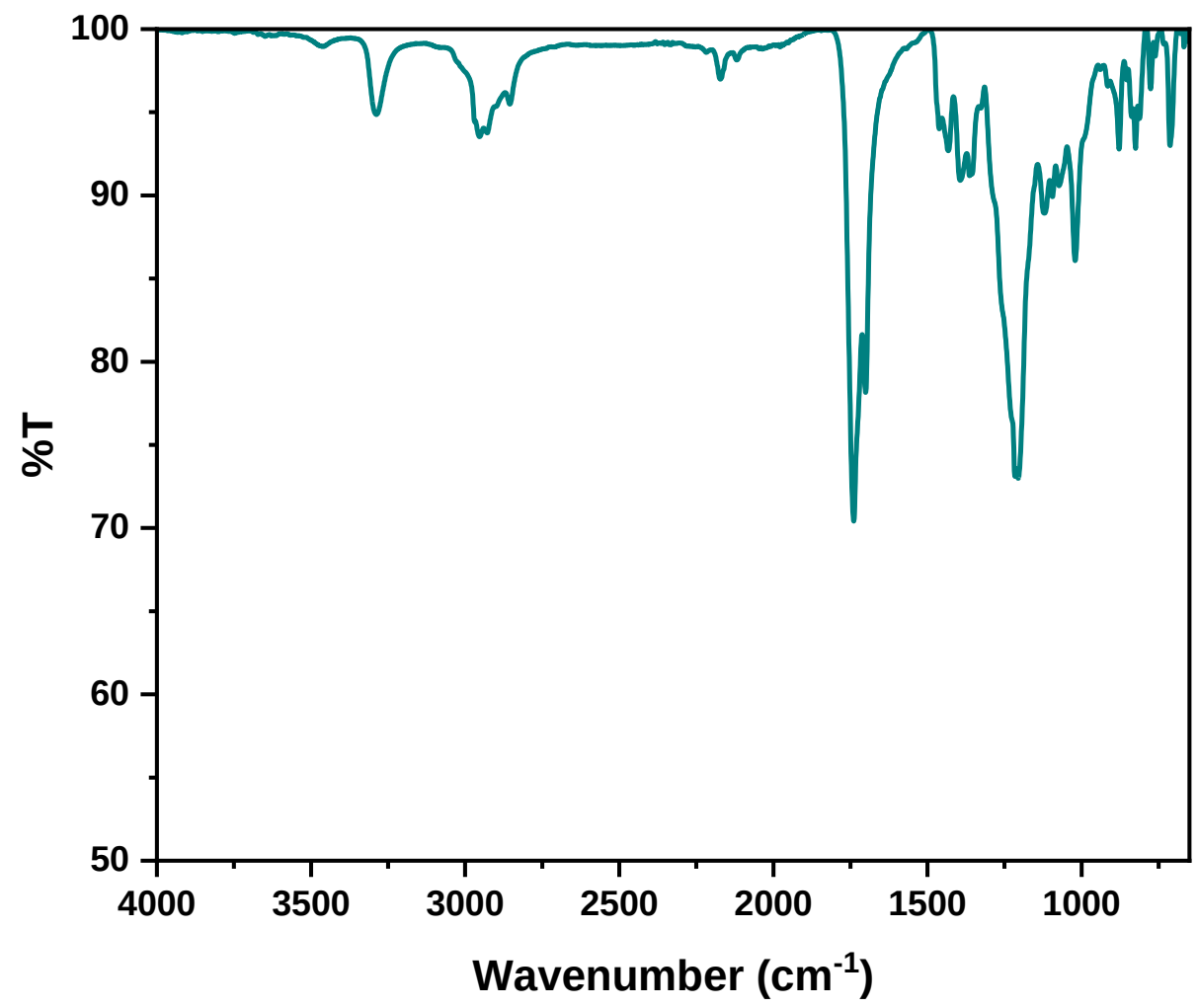

Figure S19. *FT-IR spectrum of compound **4**.*

Compound 5

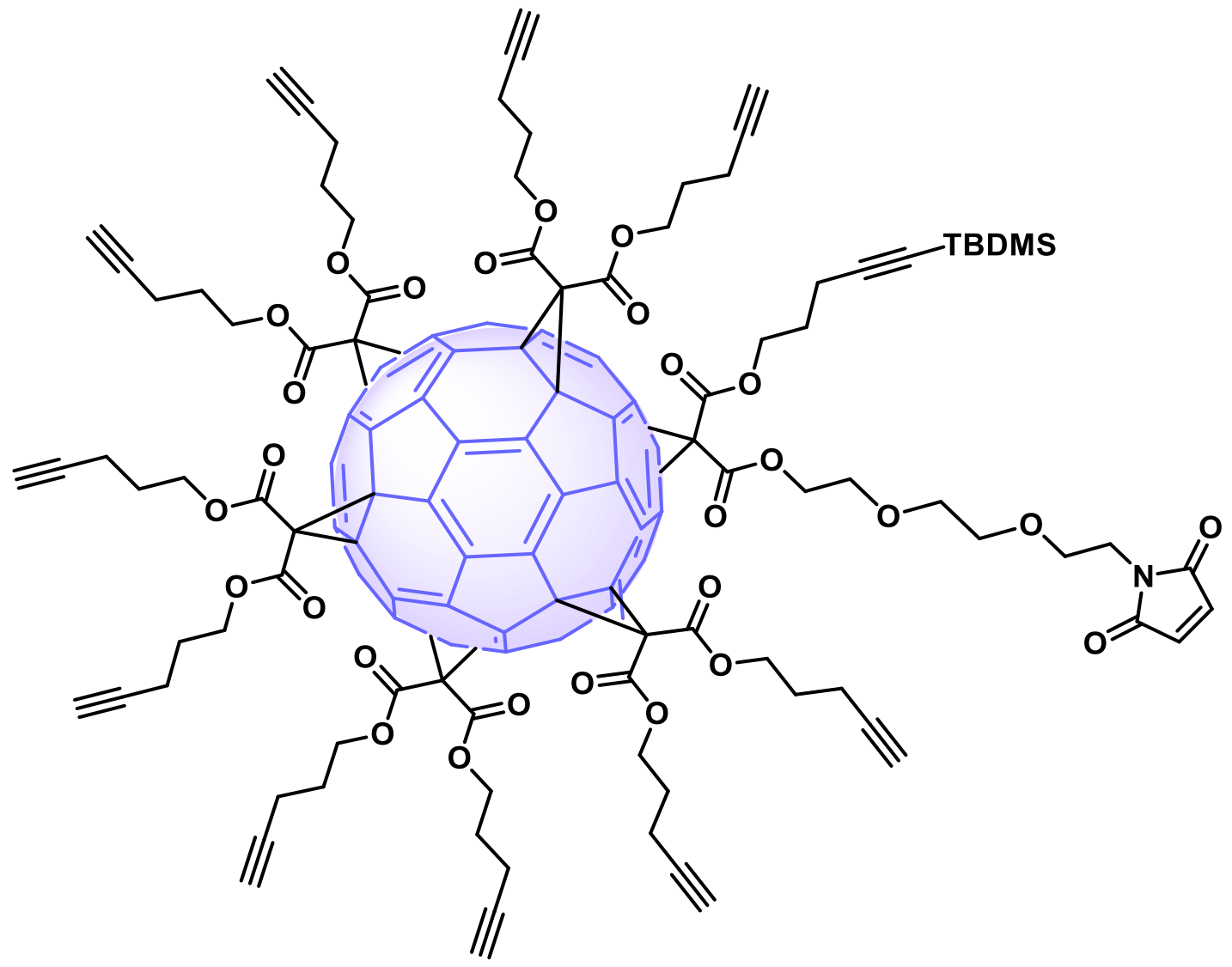

Compound **4** (300 mg, 0.12 mmol) was dissolved in dry toluene (23 mL) under argon atmosphere, and the solution was heated to reflux for 24 h using a sand bath. The crude was concentrated under reduced pressure to obtain compound **5** (290 mg, quant.) as a dark red solid, without additional purification.

^{1}**H NMR** (700 MHz, $CDCl_3$) δ 6.75 – 6.63 (m, 2H, C<u>H</u>=C<u>H</u>), 4.50 – 4.16 (m, 24H, 12xC<u>H</u>$_2$OCO), 3.74 – 3.53 (m, 10H, C<u>H</u>$_2$N$_{maleimide}$, 4xC<u>H</u>$_2$O), 2.46 – 2.21 (m, 22H, 11xC≡CC<u>H</u>$_2$CH$_2$), 2.08 – 1.74 (m, 32H, 11xC≡CC<u>H</u>$_2$CH$_2$,10xC≡C<u>H</u>), 0.91 (s, 9H, tBuSi), 0.07 (s, 6H, 2xC<u>H</u>$_3$Si). ^{13}**C NMR** (176 MHz, $CDCl_3$) δ 170.8 (CO$_{maleimide}$), 163.8 (CO), 145.9, 141.2 (C$_{sp2,fullerene}$), 134.3 (<u>C</u>H=<u>C</u>H), 106.1 (TBDMS-C≡<u>C</u>), 83.8 (TBDMS-<u>C</u>≡C), 82.5 (<u>C</u>≡CH), 78.5 (C≡<u>C</u>H), 70.7, 70.2 (CH$_2$O), 69.9 (C≡<u>C</u>H), 69.2 (C$_{sp3,fullerene}$), 68.7, 68.0 (CH$_2$O), 66.0, 65.8, 65.5 (<u>C</u>H$_2$OCO), 45.3 (C$_q$), 39.5 (C$_q$), 37.2 (N$_{maleimide}$-<u>C</u>H$_2$), 27.7 (TBDMS-C≡CCH$_2$<u>C</u>H$_2$), 27.2, 27.2, (HC≡CCH$_2$<u>C</u>H$_2$), 26.2 (tBuSi), 16.6 (C$_{q,tBu}$), 16.5 (TBDMS-C≡C<u>C</u>H$_2$), 15.2 (HC≡C<u>C</u>H$_2$), -4.3 (<u>C</u>H$_3$Si).

FTIR-ATR (υ, cm^{-1}): 3290 (≡C-H), 2954 (C-H), 2176 (C≡C), 1741 (C=O), 1368 (C-N), 1218 (C-O).

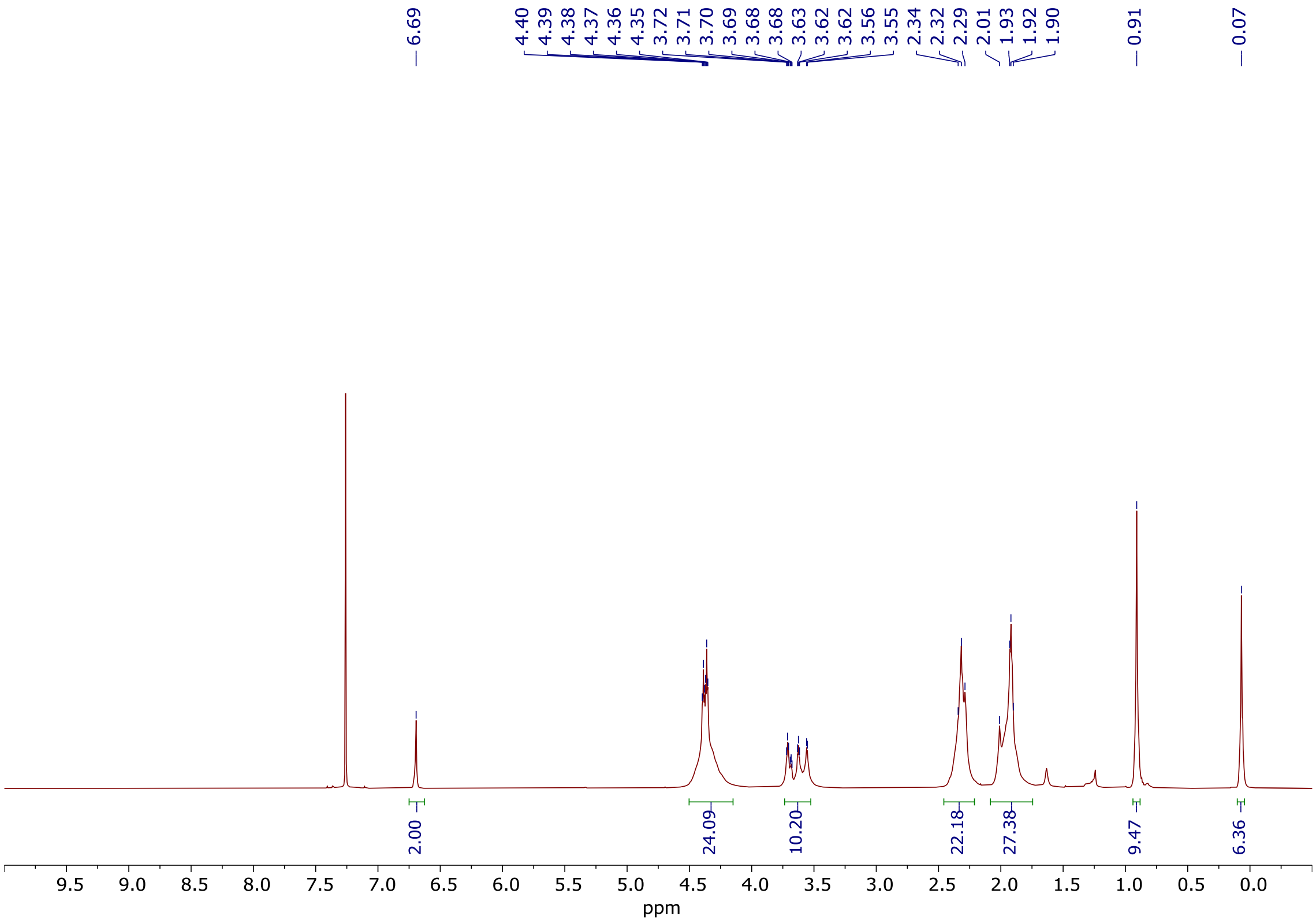

Figure S20. *¹H-NMR spectrum of compound **5** ($CDCl_3$, 700 MHz).*

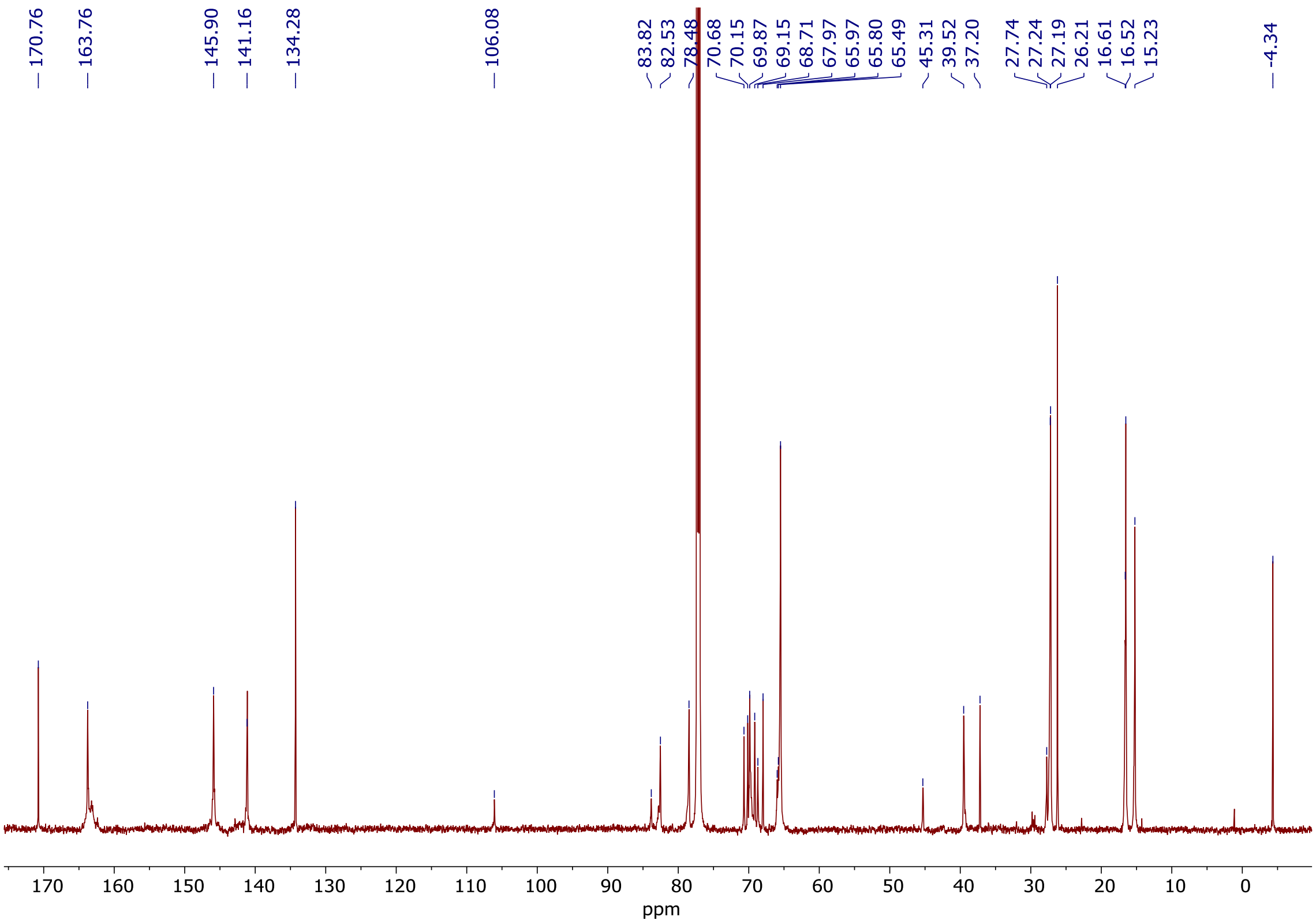

Figure S21. *¹³C-NMR spectrum of compound **5** ($CDCl_3$, 176 MHz).*

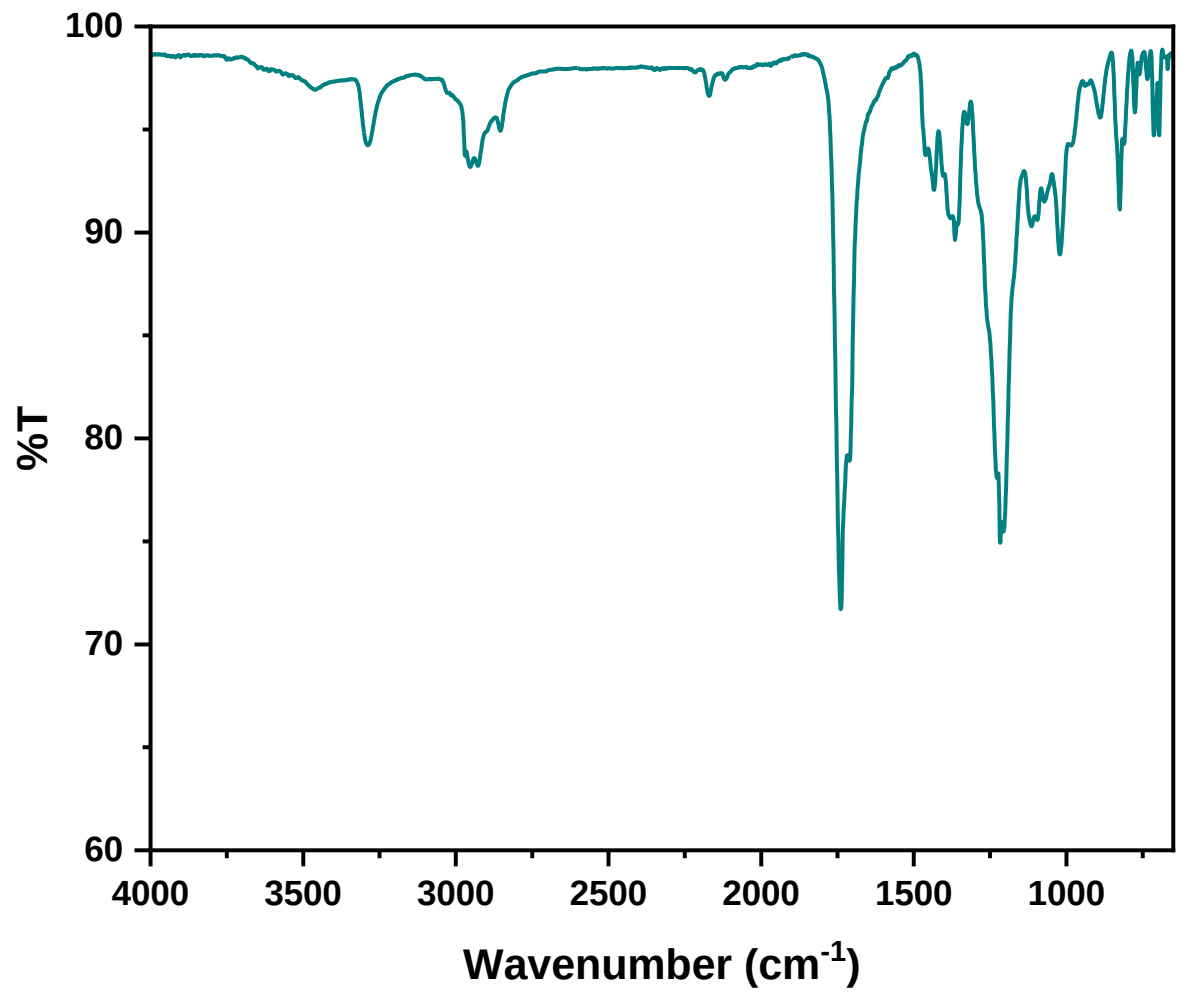

Figure S22. *FT-IR spectrum of compound **5**.*

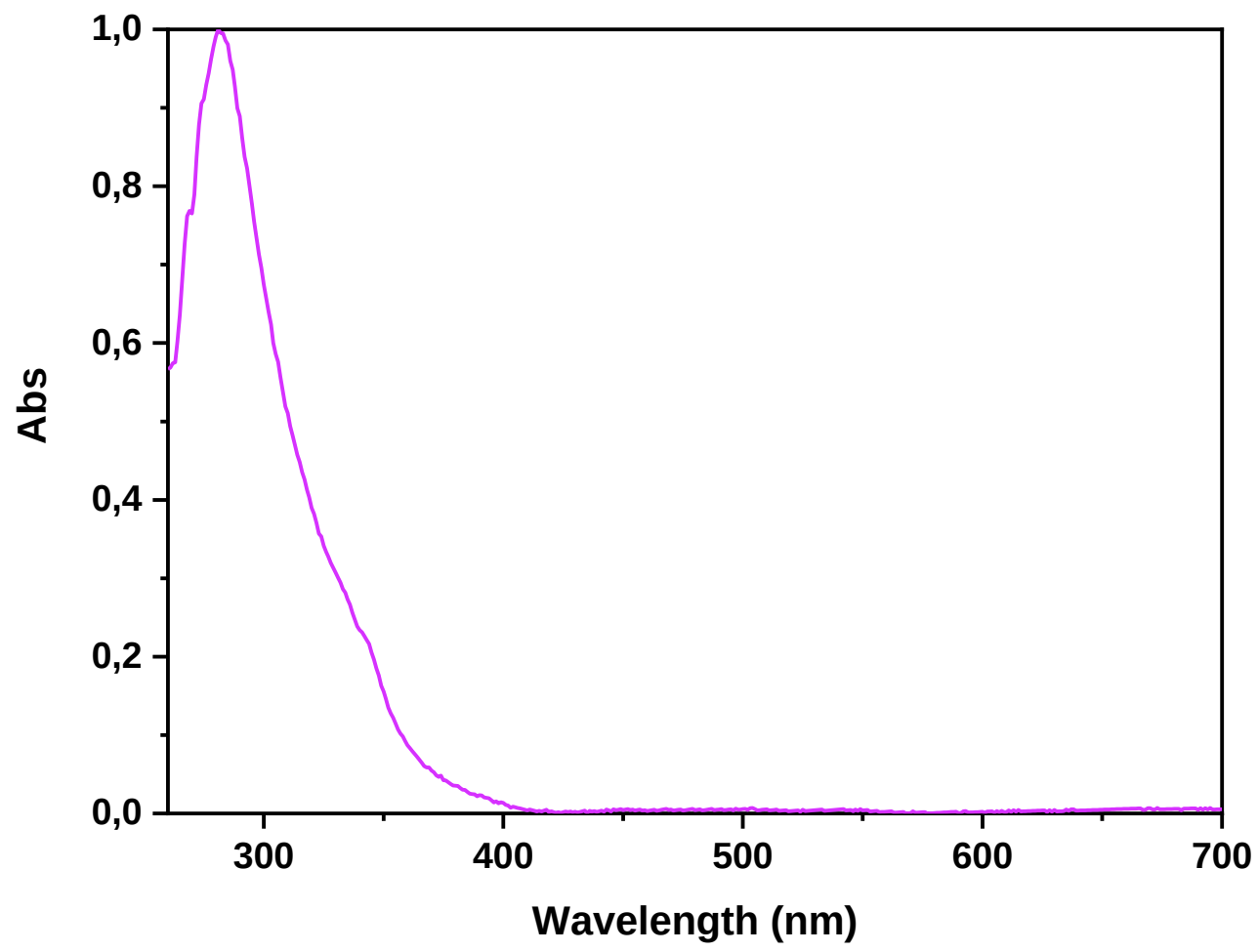

Figure S23. *UV-Vis spectrum of compound **5** ($CHCl_3$, 10^{-5} M).*

Preparation of compound 13

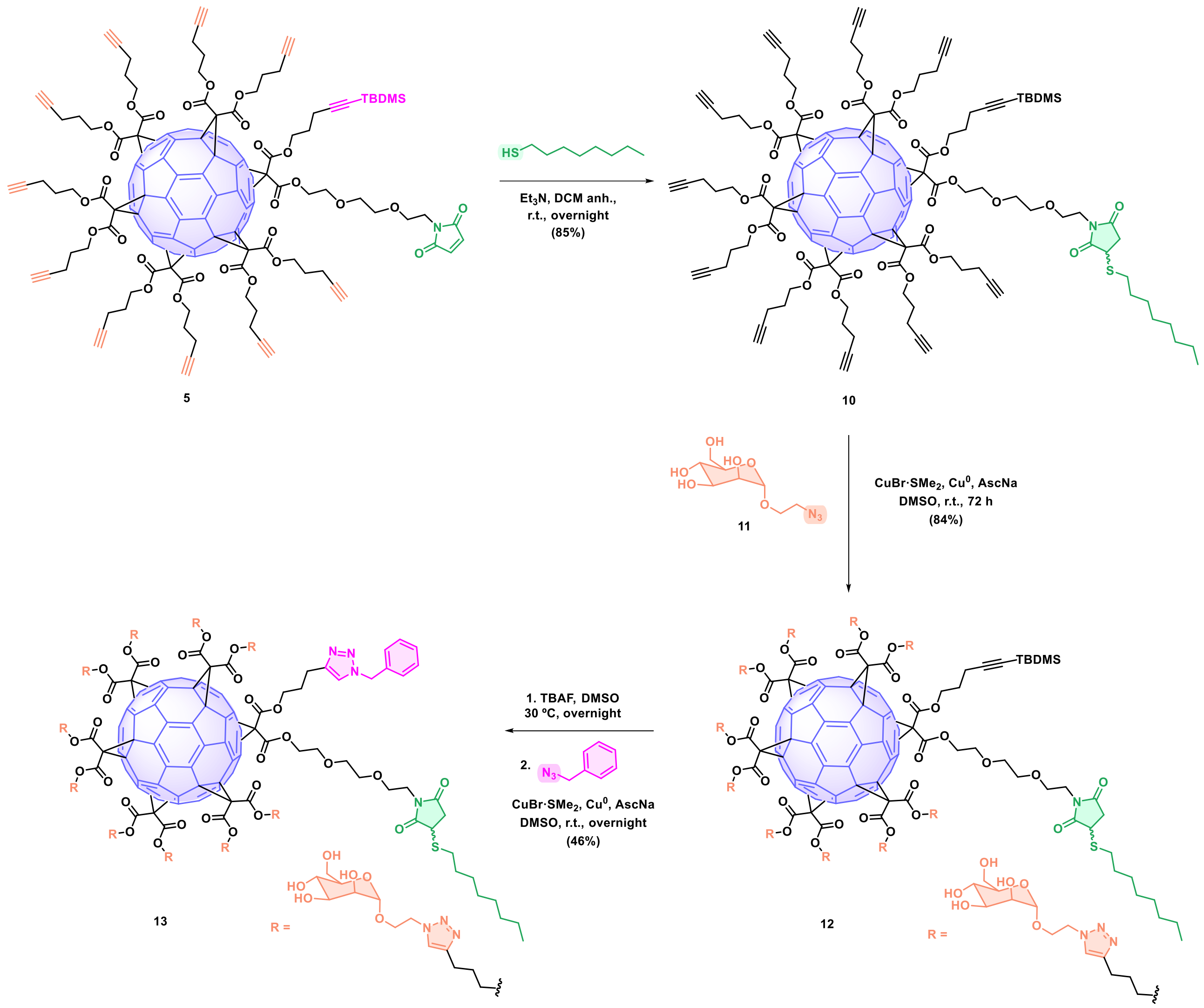

Scheme S2. Synthetic pathway to obtain hexakis-adduct **13**.

Compound 10

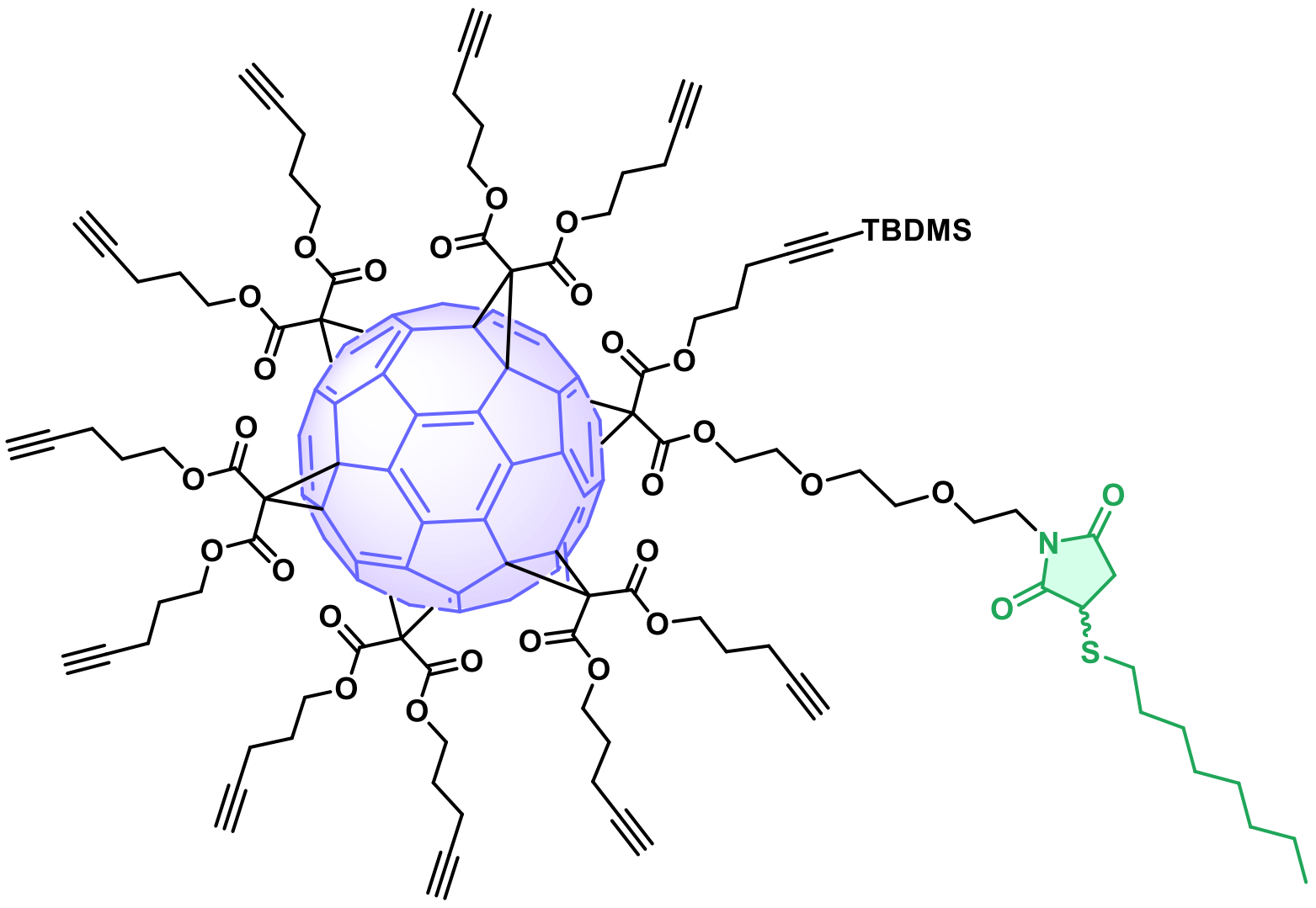

1-Octanethiol (4.40 uL, 0.025 mmol, 2 eq) was added to a solution of the hexakis-adduct **5** (30 mg, 0.013 mmol, 1 eq) and Et_3N (1.8 µL, 0.013 mmol, 1 eq) in dry DCM (1 mL) under argon atmosphere

and the mixture was stirred at room temperature for 24 hours. After that time, the solution was concentrated under reduced pressure and the obtained crude was purified by size-exclusion chromatography (Sephadex LH-20, MeOH/CH_2Cl_2 1:1), obtaining compound **10** (34 mg, quant.) as a dark red oil.

^{1}H NMR (700 MHz, $CDCl_3$) δ 4.61 – 4.17 (m, 24H, 12xCH_2OCO), 3.78 – 3.49 (m, 11H, N_{succ}-C$\underline{H}_2$, 4xC$\underline{H}_2$O, C$\underline{H}_{succ}$), 3.13 (dd, *J* = 18.8, 8.3 Hz, 1H, C$\underline{H}$$H_{succ}$), 2.85 (dt, *J* = 13.4, 6.8 Hz, 1H, SCH$\underline{H}$), 2.73 (dt, *J* = 12.8, 7.3 Hz, 1H, SC$\underline{H}$H), 2.51 (dd, *J* = 18.6, 3.8 Hz, 1H, CH$\underline{H}_{succ}$), 2.44 – 2.17 (m, 22H, 11xC≡CC$\underline{H}_2$$CH_2$), 2.08 – 1.79 (m, 32H, 11xC≡CCH_2C$\underline{H}_2$,10xC≡C$\underline{H}$), 1.66 – 1.57 (m, 2H, SCH_2C$\underline{H}_2$), 1.42 – 1.33 (m, 2H, $CH_{2,aliphatic\ chain}$), 1.33 – 1.20 (m, 10H, $CH_{2,aliphatic\ chain}$, C$\underline{H}_2$$CH_3$), 0.96 – 0.89 (s, 9H, tBuSi), 0.87 (t, *J* = 7.0 Hz, 3H, CH_2C$\underline{H}_3$), 0.07 (s, 6H, 2xC$\underline{H}_3$Si). **^{13}C NMR** (176 MHz, $CDCl_3$) δ 176.7, 174.8 (CO_{succ}), 163.8 (CO), 145.9, 141.2 ($C_{sp2,fullerene}$), 106.1 (TBDMS-C≡$\underline{C}$), 83.9 (TBDMS-$\underline{C}$≡C), 82.6 ($\underline{C}$≡CH), 78.5 (C≡$\underline{C}$H), 70.7, 70.1 (CH_2O), 69.8 (C≡$\underline{C}$H), 69.2 ($C_{sp3,fullerene}$), 68.7, 67.2 (CH_2O), 66.0, 65.8, 65.5 ($\underline{C}H_2$OCO), 45.4 (C_q), 39.5 (C_q), 39.2 (CH_{succ}), 38.3 (N_{succ}-$\underline{C}H_2$), 36.3 ($CH_{2,succ}$), 31.9, 31.8 (SCH_2), 29.3 ($SCH_2\underline{C}H_2$), 29.1, 28.9 ($CH_{2,aliphatic\ chain}$), 27.8 (TBDMS-C≡C$CH_2$$\underline{C}H_2$), 27.3 (HC≡C$CH_2$$\underline{C}H_2$), 26.2 (tBuSi), 22.8 ($\underline{C}H_2CH_3$), 16.6 ($C_{q,tBu}$), 16.5 (TBDMS-C≡C$\underline{C}H_2$), 15.3 (HC≡$\underline{CC}H_2$), 14.2 ($CH_{3,aliphatic\ chain}$), -4.3 ($\underline{C}H_3$Si).

FTIR-ATR (υ, cm^{-1}): 3293 (≡C-H), 2923 (C-H), 2172 (C≡C), 1741 (C=O), 1388 (C-N), 1213 (C-O).

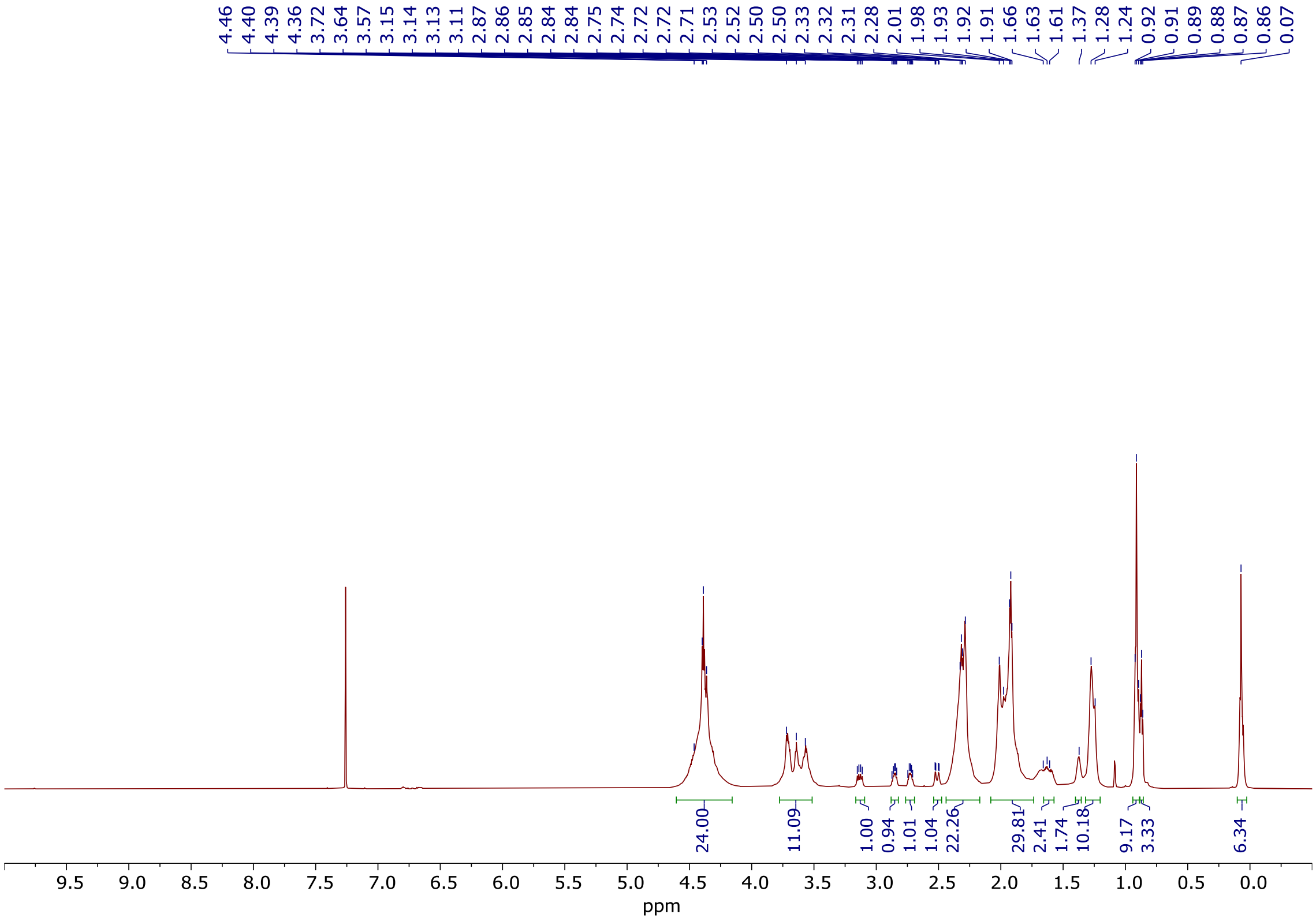

***Figure S24**. ^{1}H-NMR spectrum of compound **10** ($CDCl_3$, 700 MHz).*

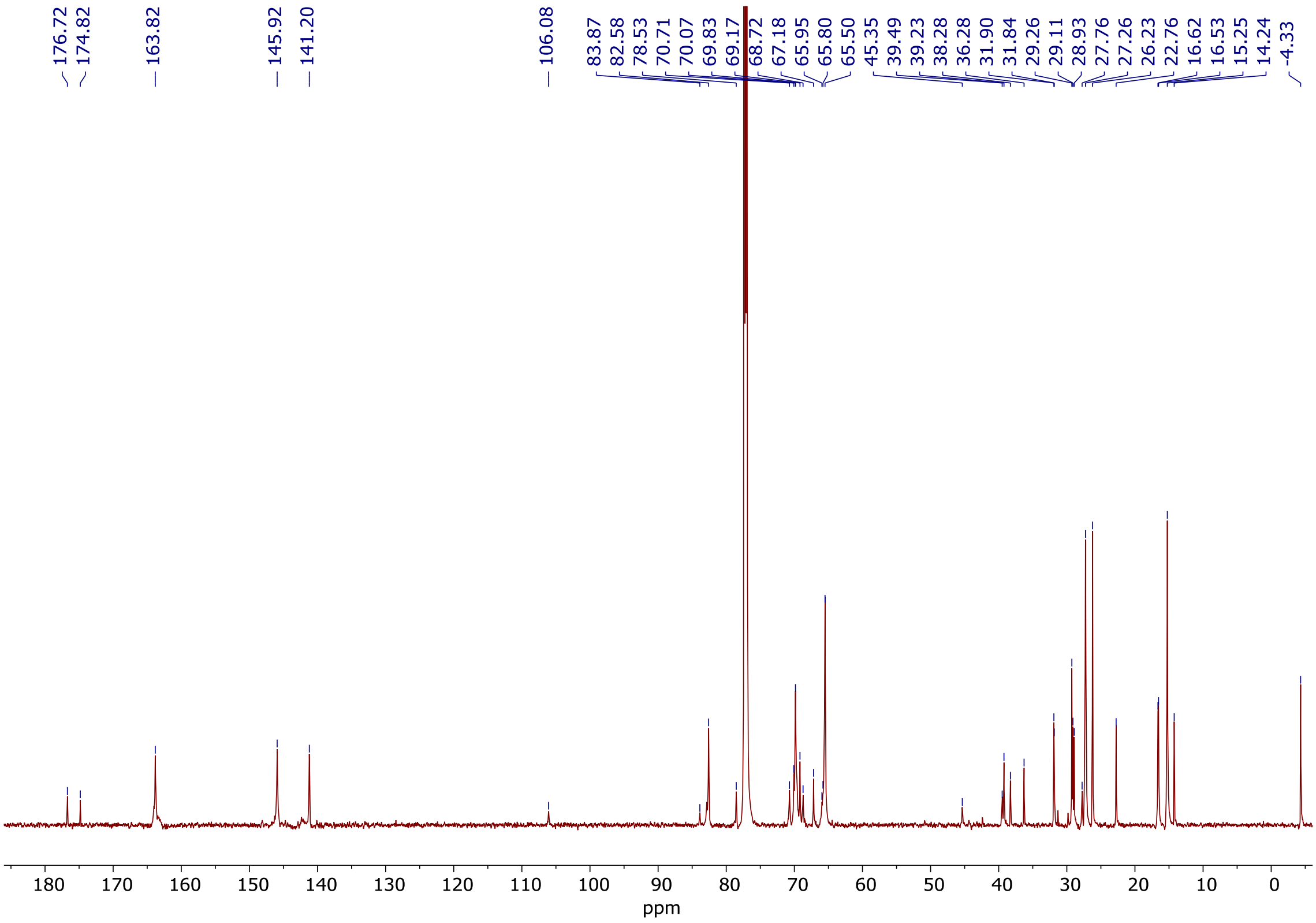

Figure S25. *^{13}C-NMR spectrum of compound **10** ($CDCl_3$, 176 MHz).*

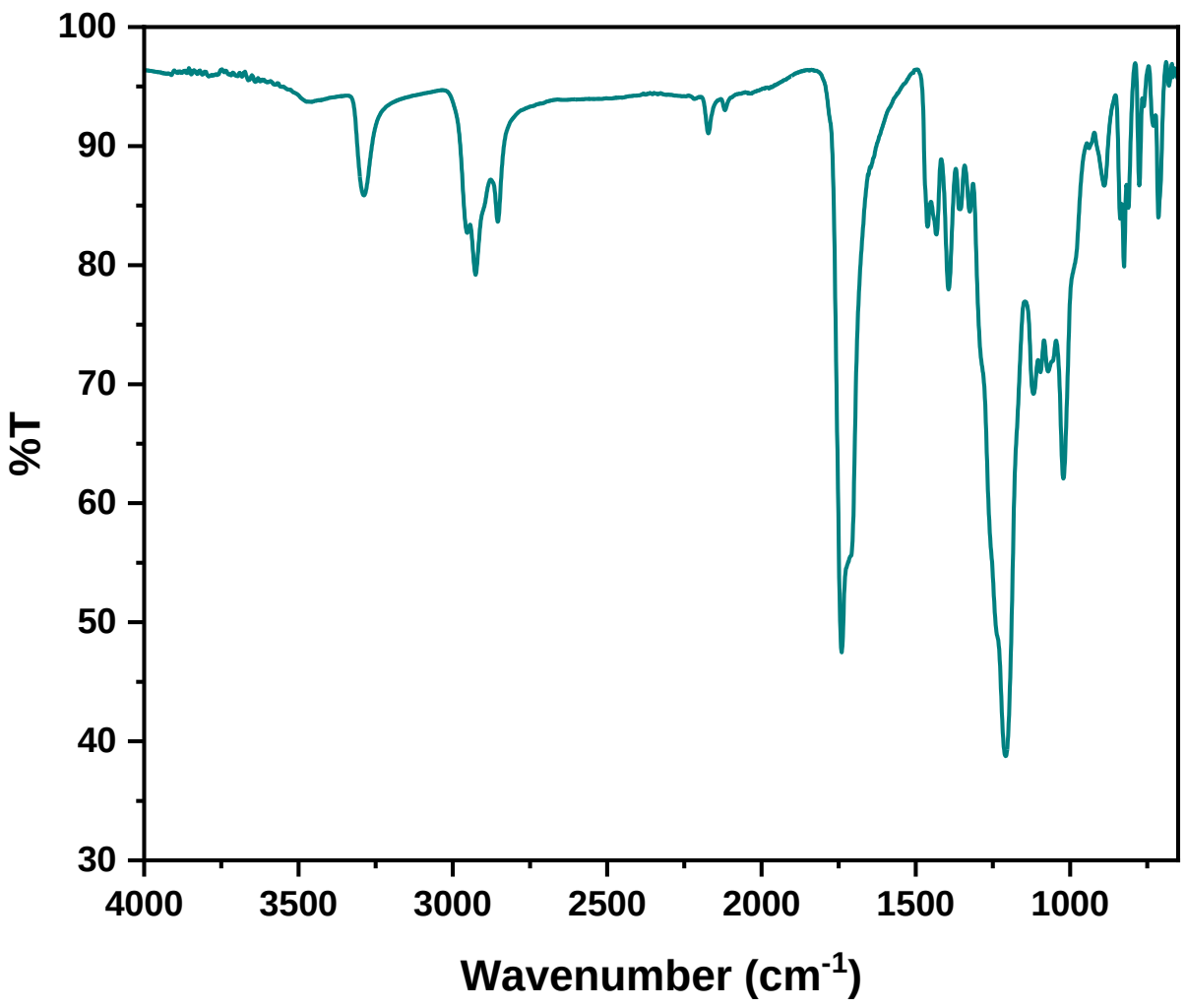

Figure S26*. FT-IR spectrum of compound **10**.*

Compound 12

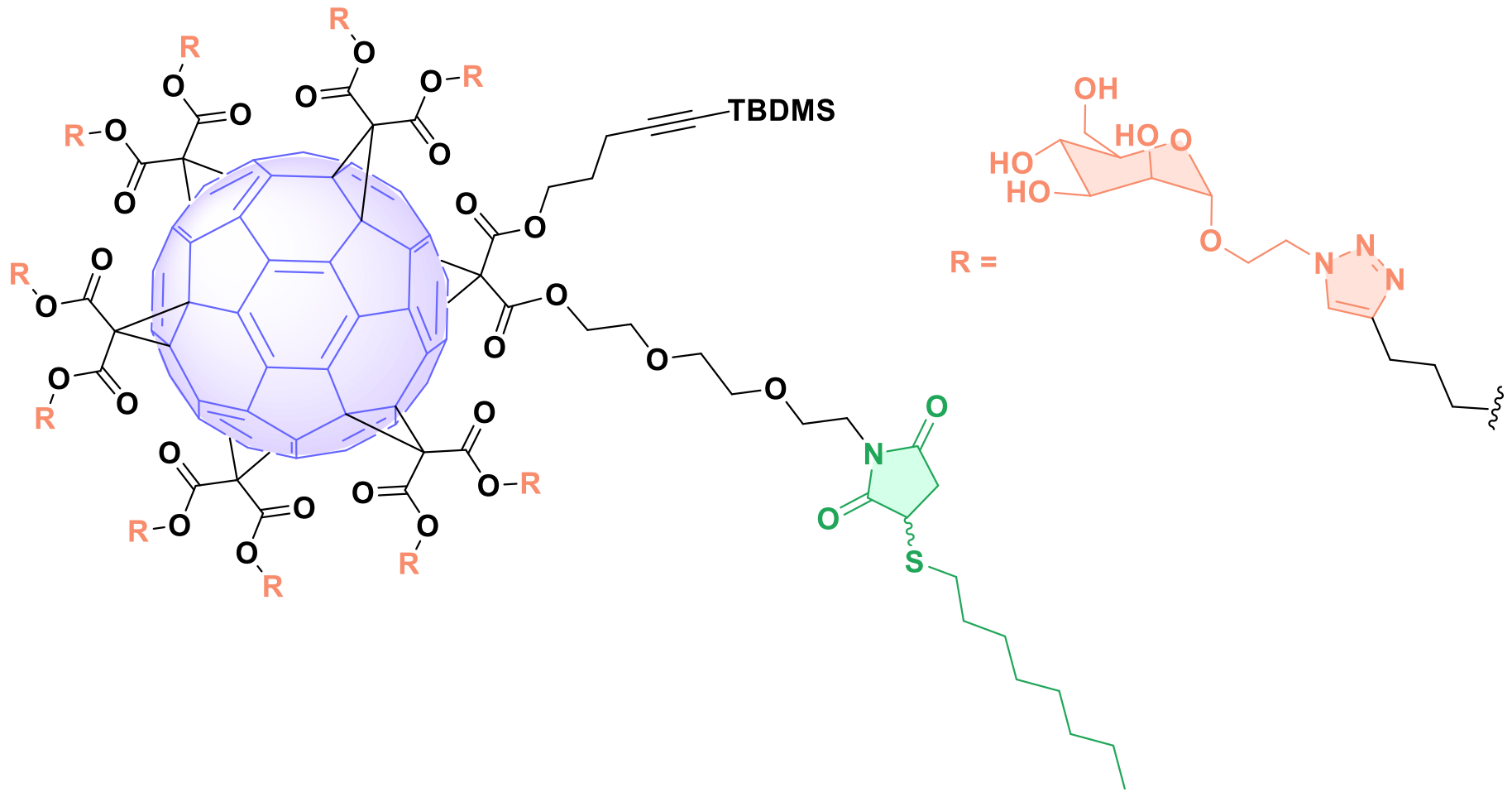

A mixture of the hexakis-adduct **10** (60 mg, 0.024 mmol, 1 eq), mannose-N_3 **11** (106 mg, 0.427 mmol, 18 eq), $CuBr{\cdot}S(CH_3)_2$ (24 mg, 0.118 mmol, 5 eq), sodium ascorbate (35 mg, 0.177 mmol, 7.5 eq) and a piece of copper metal wire in DMSO (1.5 mL) was deoxygenated and kept under argon atmosphere with vigorous stirring for 72 h. The solution was filtered through a QuadraSil® Mercaptopropyl column, and then CH_3OH was added to the mixture. The obtained precipitate was centrifuged (6000 r.p.m., 10 min) and washed three times with CH_3OH, then dried under high vacuum to give compound **12** (100 mg, 84%) as a brown solid.

^{1}H NMR (700 MHz, DMSO-d_6) δ 7.82 (s, 10H, $CH_{triazole}$), 4.73 – 4.63 (m, 20H, OH), 4.57 – 4.39 (m, 50H, $CH_{1,man}$, $OCH_2C\underline{H}_2N_{man}$, OH), 4.35 – 4.10 (m, 24H, $12xCH_2OCO$), 3.92 – 3.83 (m, 11H, $OC\underline{H}_2CH_2N_{man}$, CH_{succ}), 3.76 – 3.68 (m, 10H, $OC\underline{H}_2CH_2N_{man}$), 3.58 – 3.52 (m, 10H, $10xC\underline{H}_2OH$), 3.50 – 3.37 (m, 50H, $4xC\underline{H}_2O$, $30xCH_{man}$, N_{succ}-$C\underline{H}_2$, $10xC\underline{H}_2OH$), 3.10 – 3.01 (m, 12H, $10xCH_{man}$, $CH_{2,succ}$), 2.73 – 2.53 (m, 22H, SCH_2, $10xCOOCH_2CH_2C\underline{H}_2$), 2.28 – 2.20 (m, 2H, TBDMS-C≡$CC\underline{H}_2$), 2.03 – 1.80 (m, 22H, $11xCOOCH_2C\underline{H}_2CH_2$), 1.49 – 1.40 (m, 2H, $CH_{2,aliphatic\ chain}$), 1.24 – 1.12 (m, 10H, $CH_{2,aliphatic\ chain}$, $C\underline{H}_2CH_3$), 0.83 – 0.74 (m, 12H, tBuSi, CH_3), -0.03 (s, 6H, $2xC\underline{H}_3Si$). **^{13}C NMR** (176 MHz, DMSO-d_6) δ 176.7, 175.1 (CO_{succ}), 162.9 (CO), 145.8 ($C_{triazole}$), 145.1, 140.8 ($C_{sp2,fullerene}$), 122.5 ($CH_{triazole}$), 106.9 (TBDMS-C≡$\underline{C}$), 99.8 ($CH_{1,man}$), 83.2 (TBDMS-$\underline{C}$≡C), 74.1, 70.8, 70.1 (CH_{man}), 69.7, 69.4 (CH_2O), 68.7 ($C_{sp3,fullerene}$), 68.0 ($COOCH_2$), 66.7 (CH_{man}), 66.3 ($COOCH_2$), 65.7 ($COOCH_2$), 64.9 ($O\underline{C}H_2CH_2N_{man}$), 61.1 ($CH_2OH_{man}$), 49.3 ($OCH_2\underline{C}H_2N_{man}$), 37.8 ($N_{succ}$-$\underline{C}H_2$), 35.9 ($CH_{2,succ}$), 31.3 ($CH_{2,aliphatic\ chain}$), 30.2 ($SCH_2$), 28.6 ($SCH_2\underline{C}H_2$), 28.5 ($CH_{2,aliphatic\ chain}$), 28.2 (TBDMS-C≡$CCH_2\underline{C}H_2$) 27.8 ($COOCH_2\underline{C}H_2CH_2$), 25.9 (tBuSi), 22.1 ($\underline{C}H_2CH_3$), 21.5 ($COOCH_2CH_2\underline{C}H_2$), 16.2 ($C_{q,tBu}$), 15.7 (TBDMS-C≡$C\underline{C}H_2$), 14.0 ($CH_{3,aliphatic\ chain}$), -4.5 ($\underline{C}H_3Si$).

FTIR-ATR (υ, cm^{-1}): 3339 (OH), 2920 (C-H), 1740 (C=O), 1218 (C-O), 1025 (C-O-C).

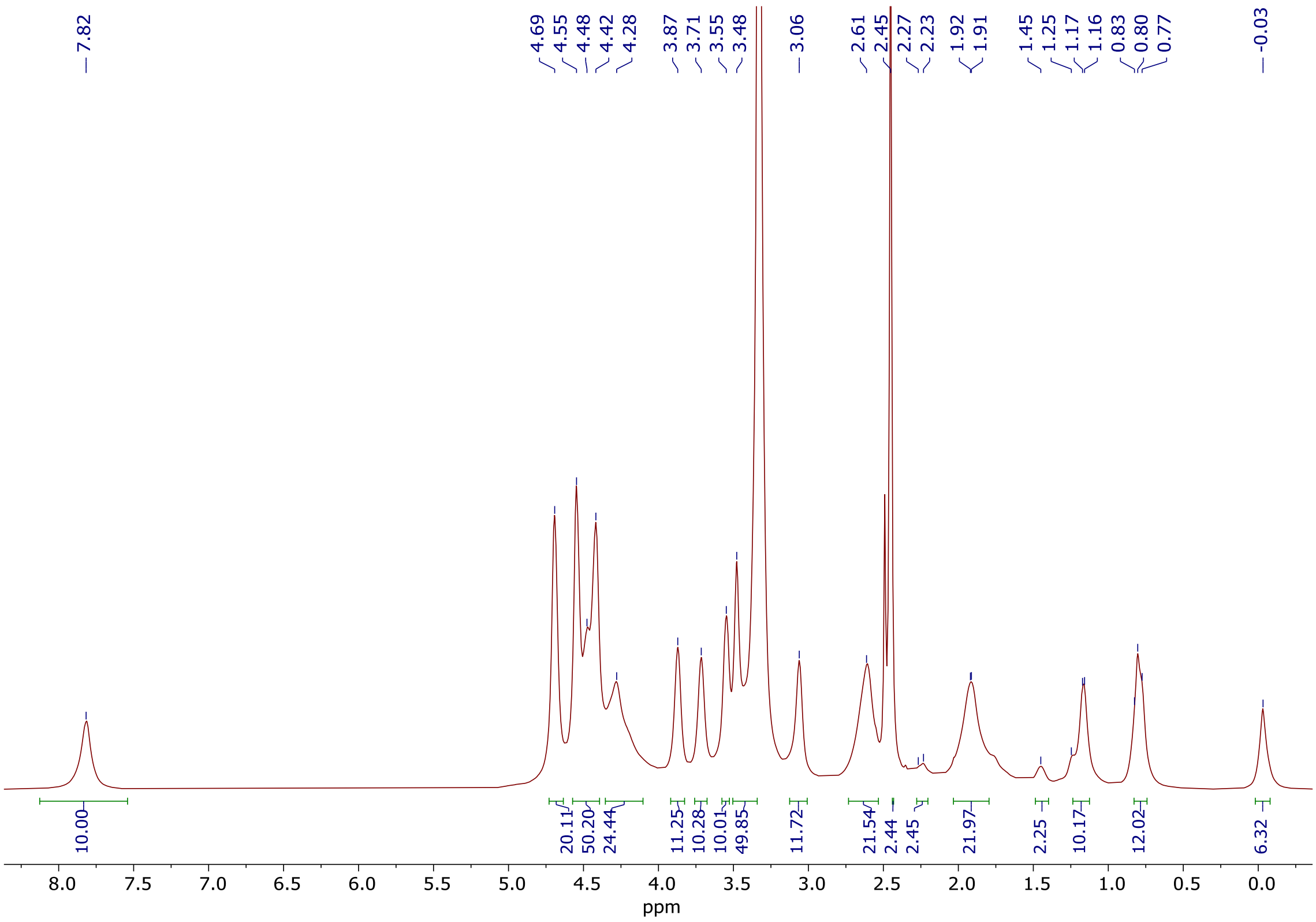

Figure S27. *^{1}H-NMR spectrum of compound **12** (DMSO-d$_6$, 700 MHz).*

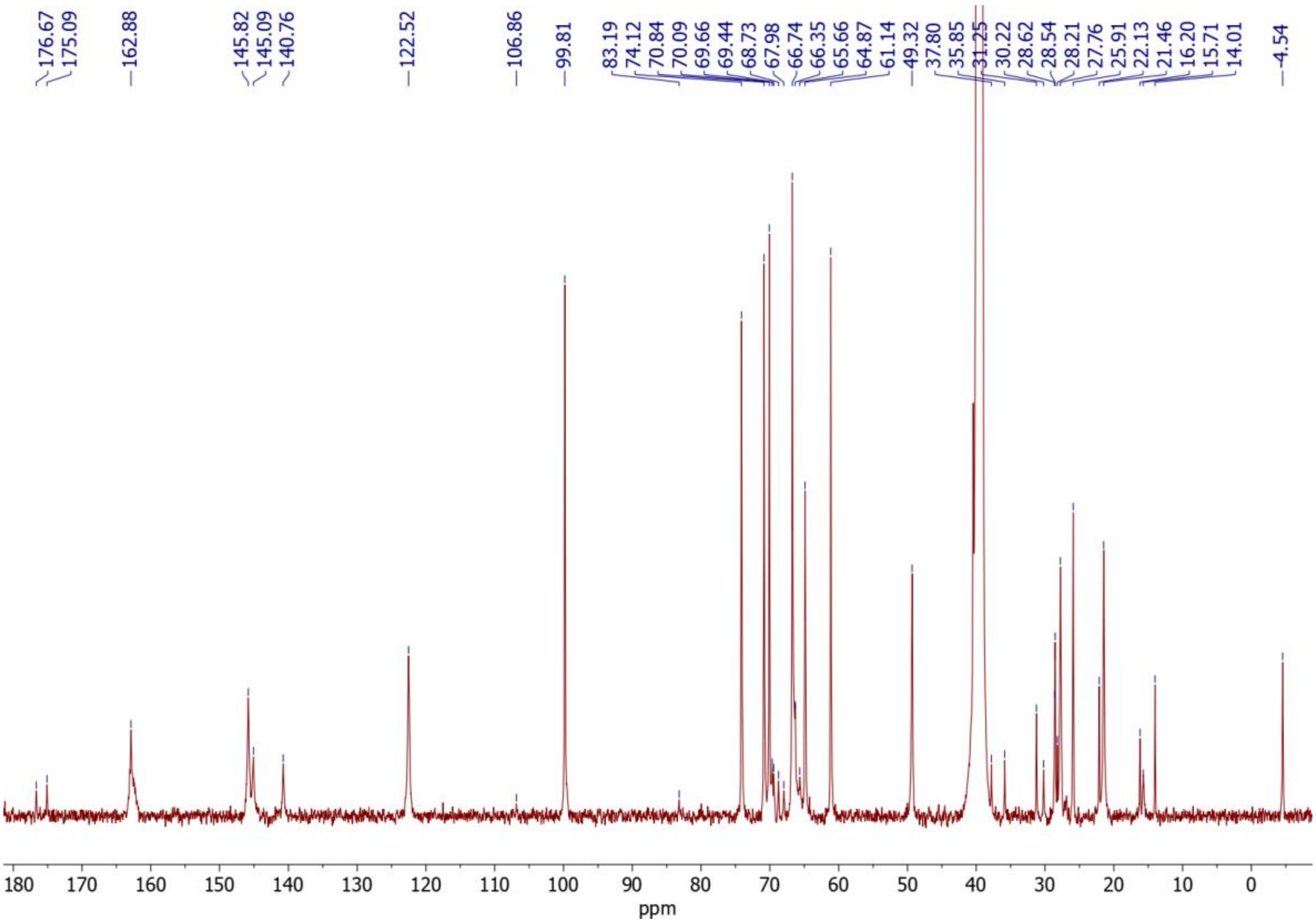

Figure S28. *^{13}C-NMR spectrum of compound **12** (DMSO-d$_6$, 176 MHz).*

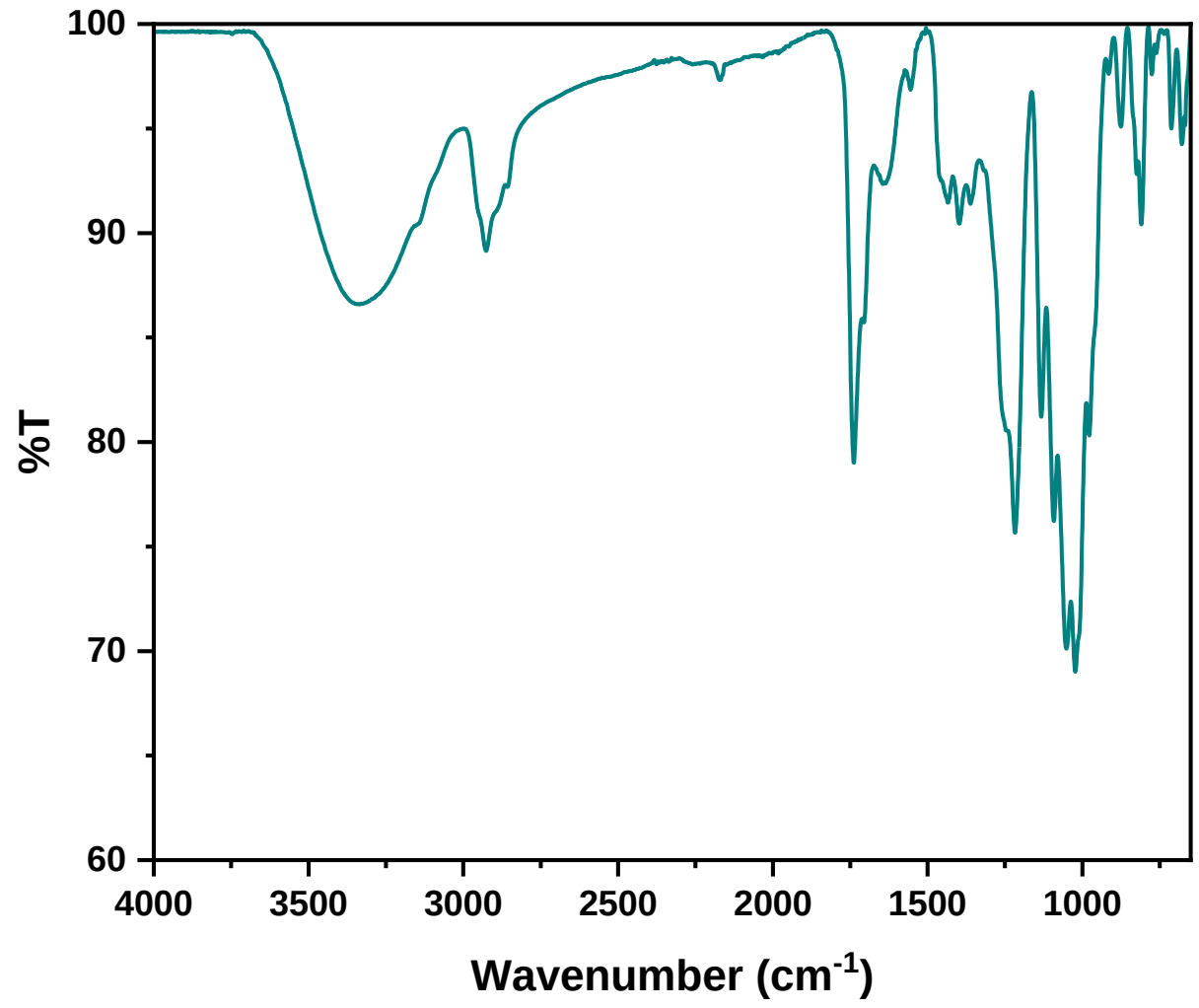

***Figure S29**. FT-IR spectrum of compound **12**.*

Compound 13

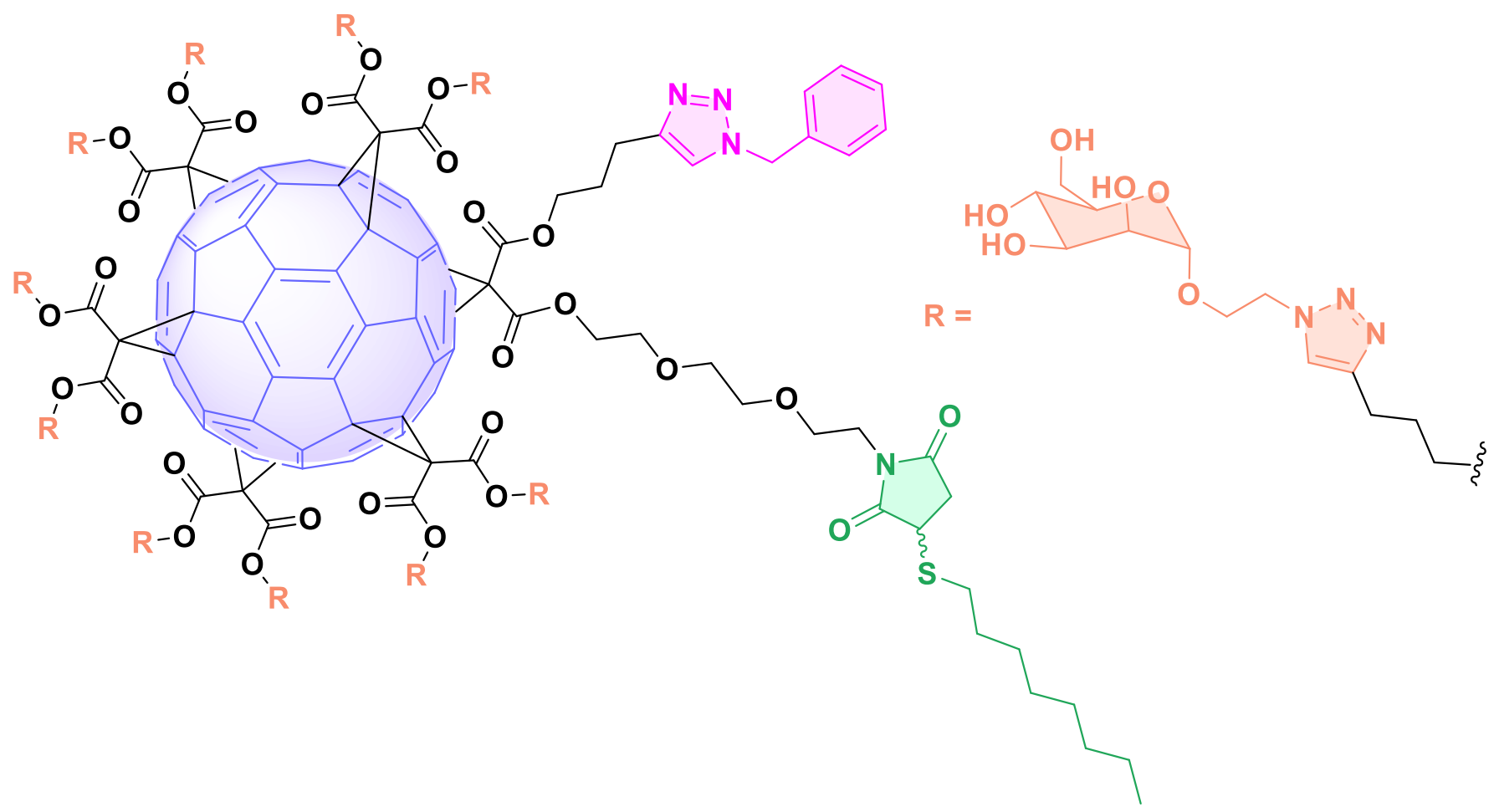

TBAF (1M in THF, 0.06 mL, 8 eq.) was added to a solution of the glycofullerene **12** (35 mg, 0.007 mmol, 1 eq.) in DMSO (0.7 mL). The solution was heated to 30 ºC using a sand bath and kept under stirring for 24 h. Then, benzyl azide (0.54 uL, 0.58x10^{-3} mmol, 1.25 eq), CuBr·$S(CH_3)_2$ (0.72 mg, 0.0035 mmol, 1 eq), sodium ascorbate (1.04 mg, 0.0052 mmol, 1.5 eq) and a piece of copper metal wire were added to the mixture and it was deoxygenated and kept under argon atmosphere with vigorous stirring for 72 h. The crude was filtered through a QuadraSil® Mercaptopropyl column, and then AcOEt was added to the mixture. The obtained precipitate was centrifuged (6000 r.p.m., 10 mins) and washed three times with AcOEt, then dried under high vacuum to give compound **13** (16 mg, 46%) as a brown solid.

^{1}H NMR (700 MHz, DMSO-d_6) δ 7.94 – 7.65 (m, 10H, $CH_{triazole}$), 7.34 – 7.29 (m, 1H, $CH_{triazole}$), 7.28 – 7.20 (m, 5H, 5xCH_{Ar}), 5.52 – 5.45 (m, 2H, CH_2-Ar), 4.85 – 4.68 (m, 20H, OH), 4.62 – 4.36 (m, 50H, $CH_{1,man}$, $OCH_2C\underline{H_2}N_{man}$, OH), 4.33 – 4.08 (m, 24H, 12xCH_2OCO), 3.91 – 3.84 (m, 11H, $OC\underline{H_2}CH_2N_{man}$, CH_{succ}), 3.75 – 3.68 (m, 10H, $OC\underline{H_2}CH_2N_{man}$), 3.58 – 3.54 (m, 10H, 10x$C\underline{H_2}OH$), 3.52 – 3.37 (m, 50H, 4x$C\underline{H_2}O$, 30xCH_{man}, N_{succ}-$C\underline{H_2}$, 10x$C\underline{H_2}OH$), 3.13 – 3.02 (m, 12H, , 10xCH_{man}, $CH_{2,succ}$), 2.77-2.61 (m, 22H, SCH_2, 11x$COOCH_2CH_2C\underline{H_2}$), 1.98 – 1.59 (m, 26H, 12x$COOCH_2C\underline{H_2}CH_2$, $CH_{2,aliphatic\ chain}$), 1.58 – 1.47 (m, 2H, $CH_{2,aliphatic\ chain}$), 1.34 – 1.01 (m, 8H, $CH_{2,aliphatic\ chain}$, $C\underline{H_2}CH_3$), 0.84 – 0.67 (m, 3H, CH_3).**^{13}C NMR** (176 MHz, DMSO-d_6) δ 145.8 ($C_{triazole}$), 128. 8 ($CH_{triazole}$), 128.0, 127.9 (CH_{Ar}), 122.3 ($CH_{triazole}$), 99.8 ($CH_{1,man}$), 74.1 (CH_{man}), 72.4 (CH_2O),

70.8, 70.1 (CH_{man}), 69.7 (CH_2O), 66.8 (CH_{man}), 64.9 (O<u>C</u>$H_2CH_2N_{man}$), 61.1 (CH_2OH_{man}), 52.8 (CH_2-Ar), 49.2 (OCH_2<u>C</u>H_2N_{man}), 47.8, 36.0 ($CH_{2,succ}$), 32.3, 31.1, 28.7, 28.6 ($CH_{2,aliphatic\ chain}$) 27.9 ($COOCH_2$<u>C</u>H_2CH_2), 22.1 (<u>C</u>H_2CH_3), 21.5 ($COOCH_2CH_2$<u>C</u>H_2), 14.0 ($CH_{3,aliphatic\ chain}$).

FTIR-ATR (υ, cm^{-1}): 3380 (OH), 2922 (C-H st.), 1721 (C=O), 1635 (C=C) 1223 (C-O), 1023 (C-O-C).

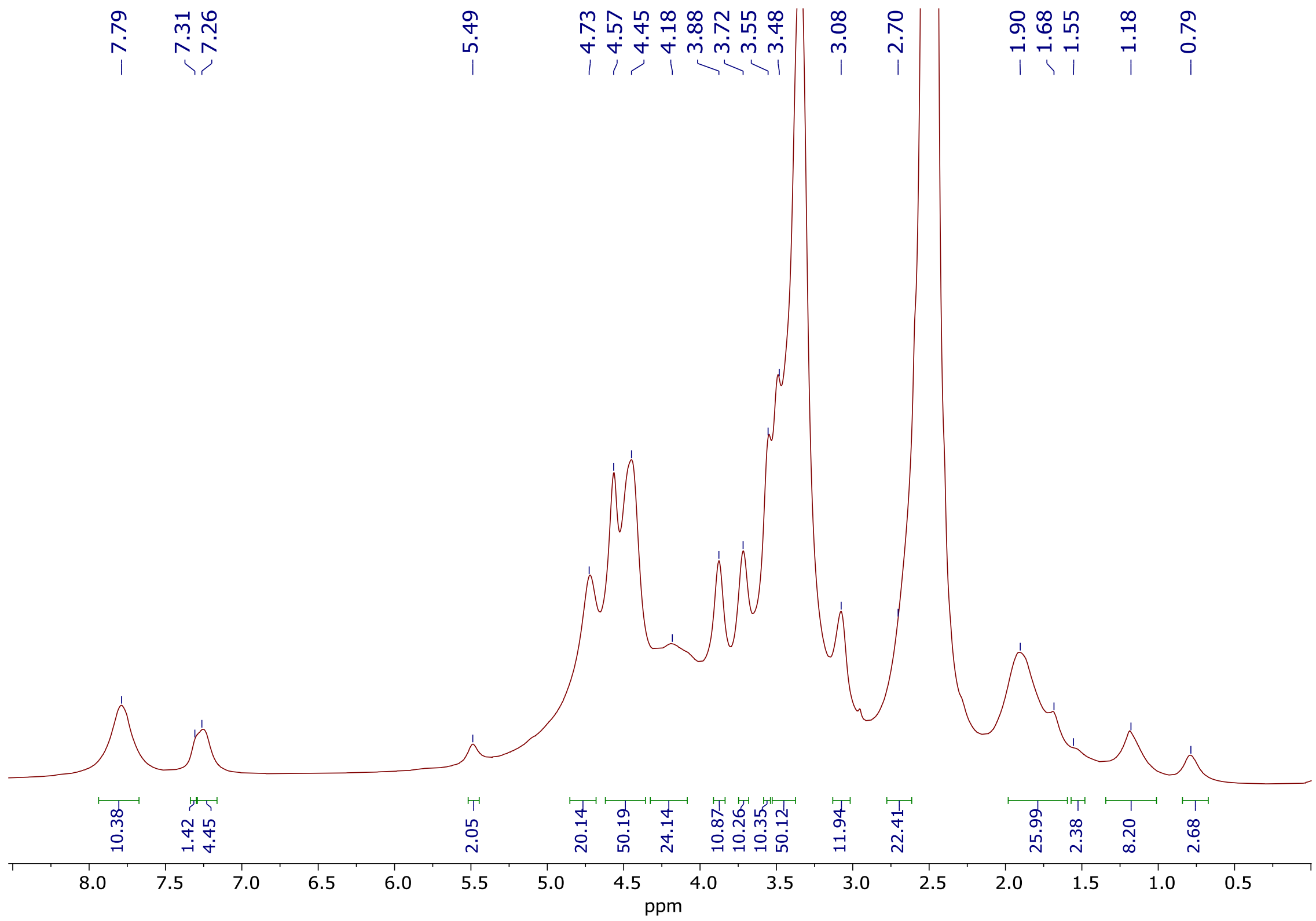

Figure S30. *^{1}H-NMR spectrum of compound **13** (DMSO-d$_6$, 700 MHz).*

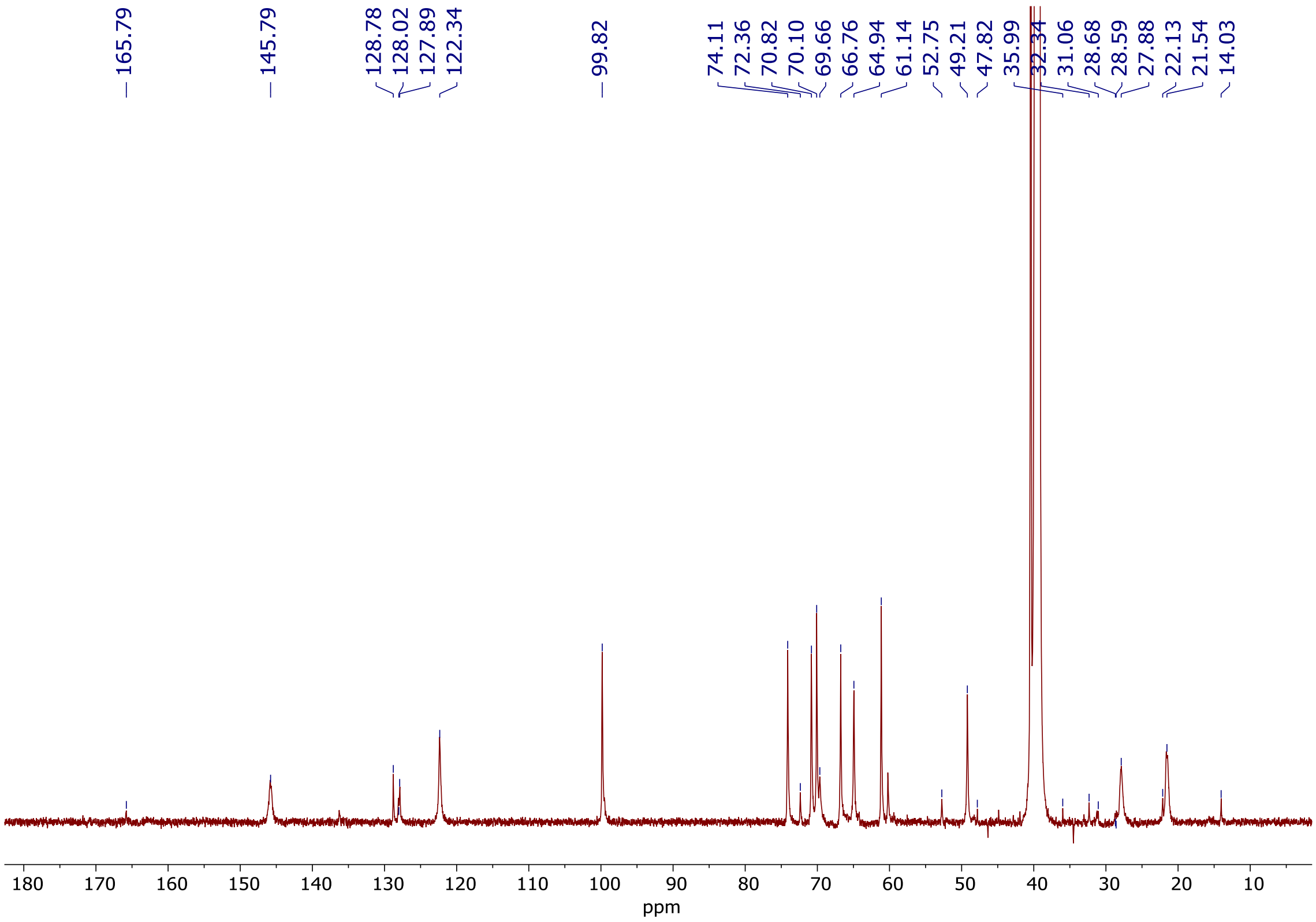

Figure S31. *^{13}C-NMR spectrum of compound* ***13*** *(DMSO-d$_6$, 176 MHz).*

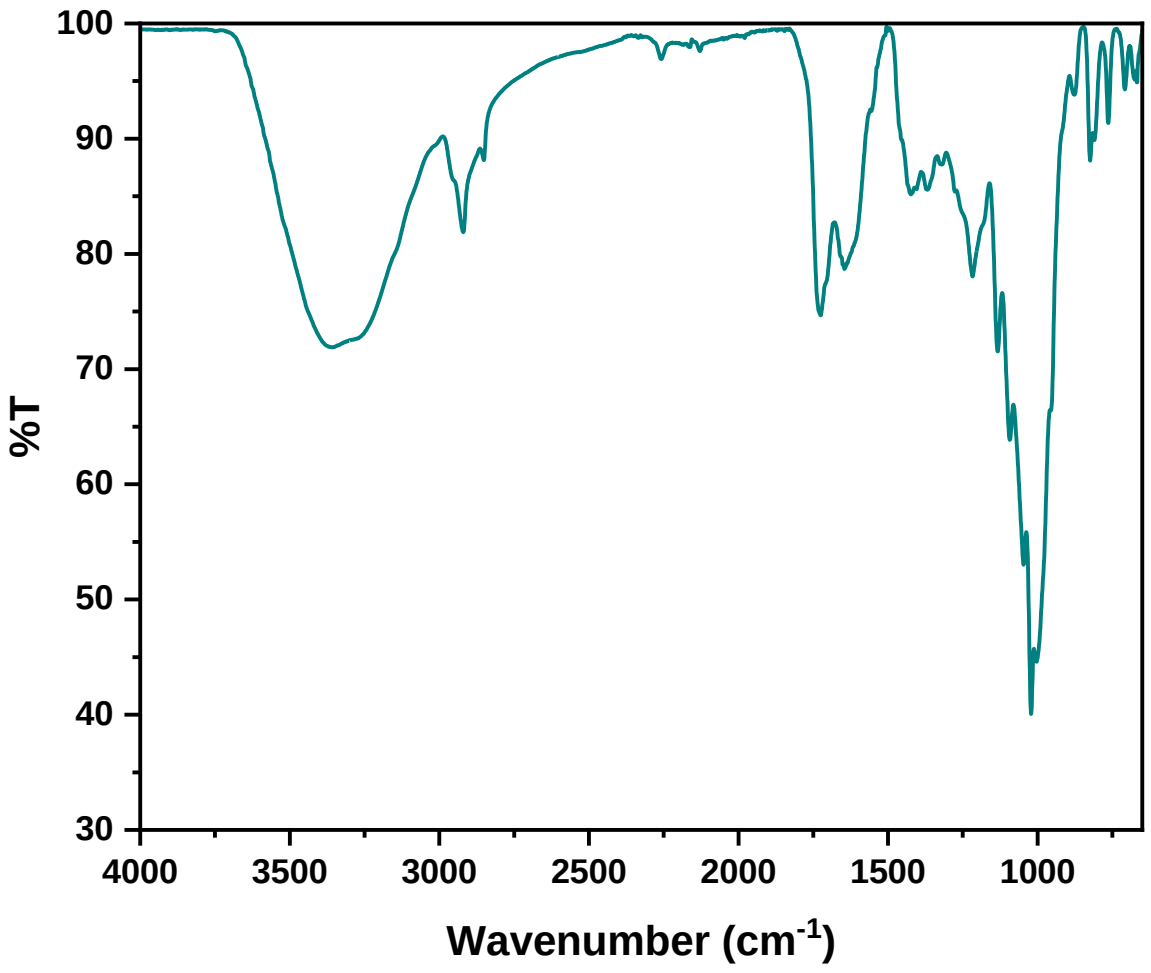

Figure S32. *FT-IR spectrum of compound* ***13****.*

Compound 15

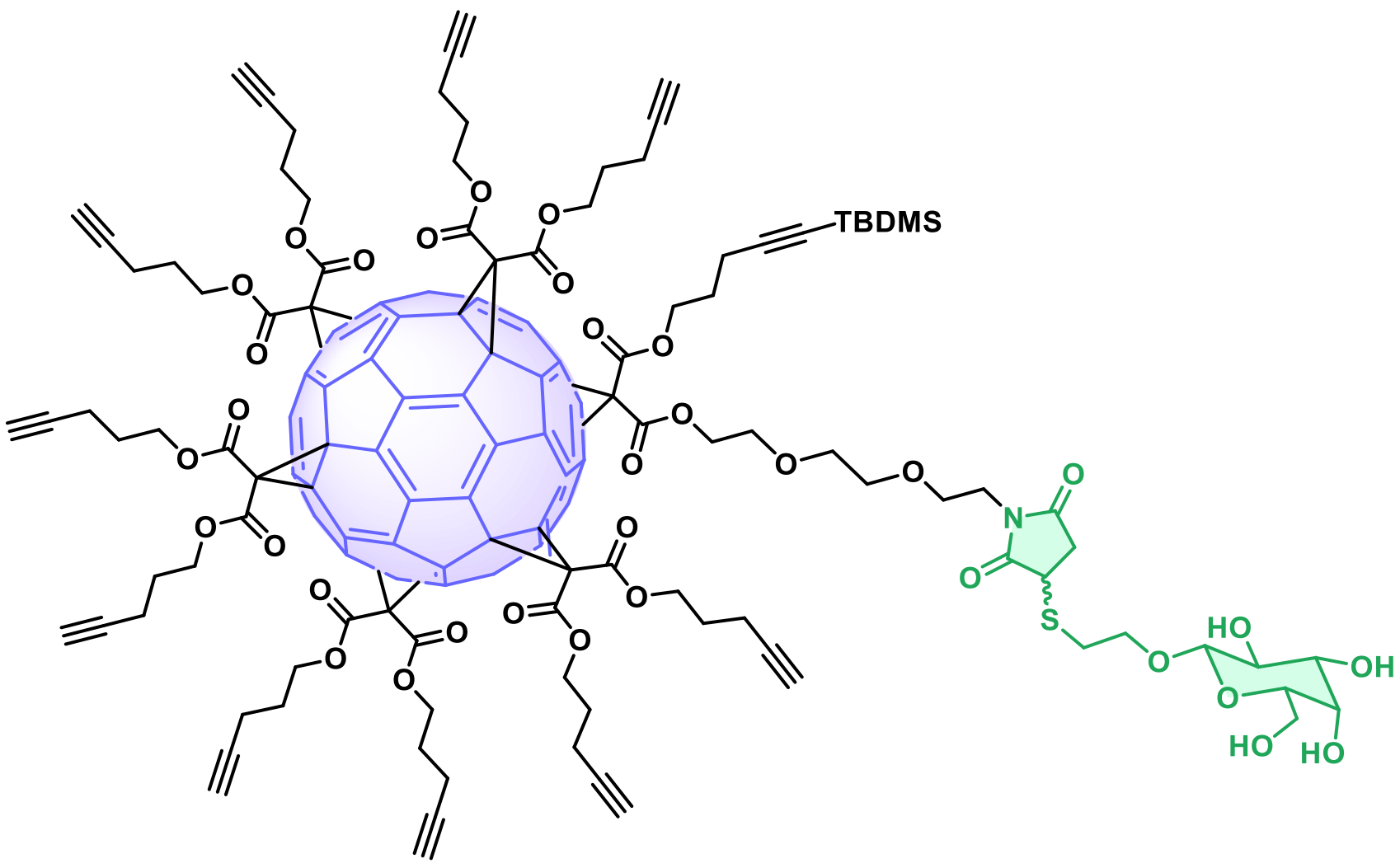

Galactose-SH **14** (10 mg, 0.042 mmol, 2 eq) was added to a solution of the hexakis-adduct **5** (50 mg, 0.021 mmol, 1 eq) and Et_3N (2.9 µL, 0.021 mmol, 1 eq) in dry DCM/MeOH (1:1) under argon atmosphere (1 mL) and the mixture was stirred at room temperature for 24 hours. After that time, the solution was concentrated under reduced pressure and the obtained crude was purified by column chromatography (silica gel, AcOEt → Acetone), obtaining compound **15** (35 mg, 77%) as a dark red oil.

^{1}H NMR (700 MHz, $CDCl_3$) δ 4.56 – 4.22 (m, 25H, CH_1, 12xCH_2OCO), 4.19 – 4.08 (m, 2H, C$\underline{H}$HO-gal, OH), 4.06 – 3.97 (m, 2H, CH_{succ}, OH), 3.97 – 3.89 (m, 2H, 2xOH), 3.89 – 3.79 (m, 3H, C$\underline{H}$HO-gal, CH_2OH), 3.76 – 3.68 (m, 4H, N_{succ}-C$\underline{H}_2$, CH_2O), 3.67 – 3.47 (m, 10H, 4xCH_{gal}, 3xCH_2O), 3.23 – 3.13 (m, 2H, SC$\underline{H}$H, CH$\underline{H}_{succ}$), 2.91 – 2.84 (m, 1H, SC$\underline{H}$H), 2.64 – 2.56 (m, 1H, CH$\underline{H}_{succ}$), 2.42 – 2.18 (m, 22H, 11xC≡CC$\underline{H}_2$$CH_2$), 2.07 – 1.74 (m, 32H, 11xC≡CCH_2C$\underline{H}_2$,10xC≡C$\underline{H}$), 0.91 (s, 9H, tBuSi), 0.07 (s, 6H, 2xC$\underline{H}_3$Si). **^{13}C NMR** (176 MHz, $CDCl_3$) δ 177.4, 175.1 (CO_{succ}), 163.8 (CO), 145.9, 141.2 ($C_{sp2,fullerene}$), 106.1 (TBDMS-C≡$\underline{C}$), 103.8, 103.7 ($CH_{1,galactose}$), 83.9 (TBDMS-$\underline{C}$≡C), 82.6 ($\underline{C}$≡CH), 78.5 (C≡$\underline{C}$H), 74.6, 74.5 ($CH_{gal,both\ isomers}$), 73.6, 73.5 ($CH_{gal,both\ isomers}$), 71.8 (CH_{gal}), 70.6 ($CH_2$$\underline{C}H_2$O-Gal), 69.9 ($CH_2$O, $CH_2$$\underline{C}H_2$O-Gal), 69.8 ($CH_{gal}$), 69.4 (C≡$\underline{C}$H), 69.2 ($C_{sp3,fullereno}$), 68.7, 67.1 ($CH_2$O), 65.5 ($\underline{C}H_2$OCO), 62.9 ($CH_2$OH), 45.4 ($C_q$), 39.7 ($C_q$), 39.5 ($CH_{succ.}$), 38.5 ($N_{succ}$-$\underline{C}H_2$), 36.7, 36.4 ($CH_{2,succ}$), 31.7, 31.5 ($SCH_2$), 29.8, 27.8 (TBDMS-C≡C$CH_2$$\underline{C}H_2$), 27.3 (HC≡C$CH_2$$\underline{C}H_2$), 26.2 (tBuSi), 16.6 ($C_{q,tBu}$), 16.5 (TBDMS-C≡C$\underline{C}H_2$), 15.3 (HC≡C$\underline{C}H_2$), -4.3 ($\underline{C}H_3$Si).

FTIR-ATR (υ, cm^{-1}): 3440 (O-H), 3296 (≡C-H), 2931 (C-H), 1743 (C=O), 1394 (C-N), 1218 (C-O), 1023 (C-O-C).

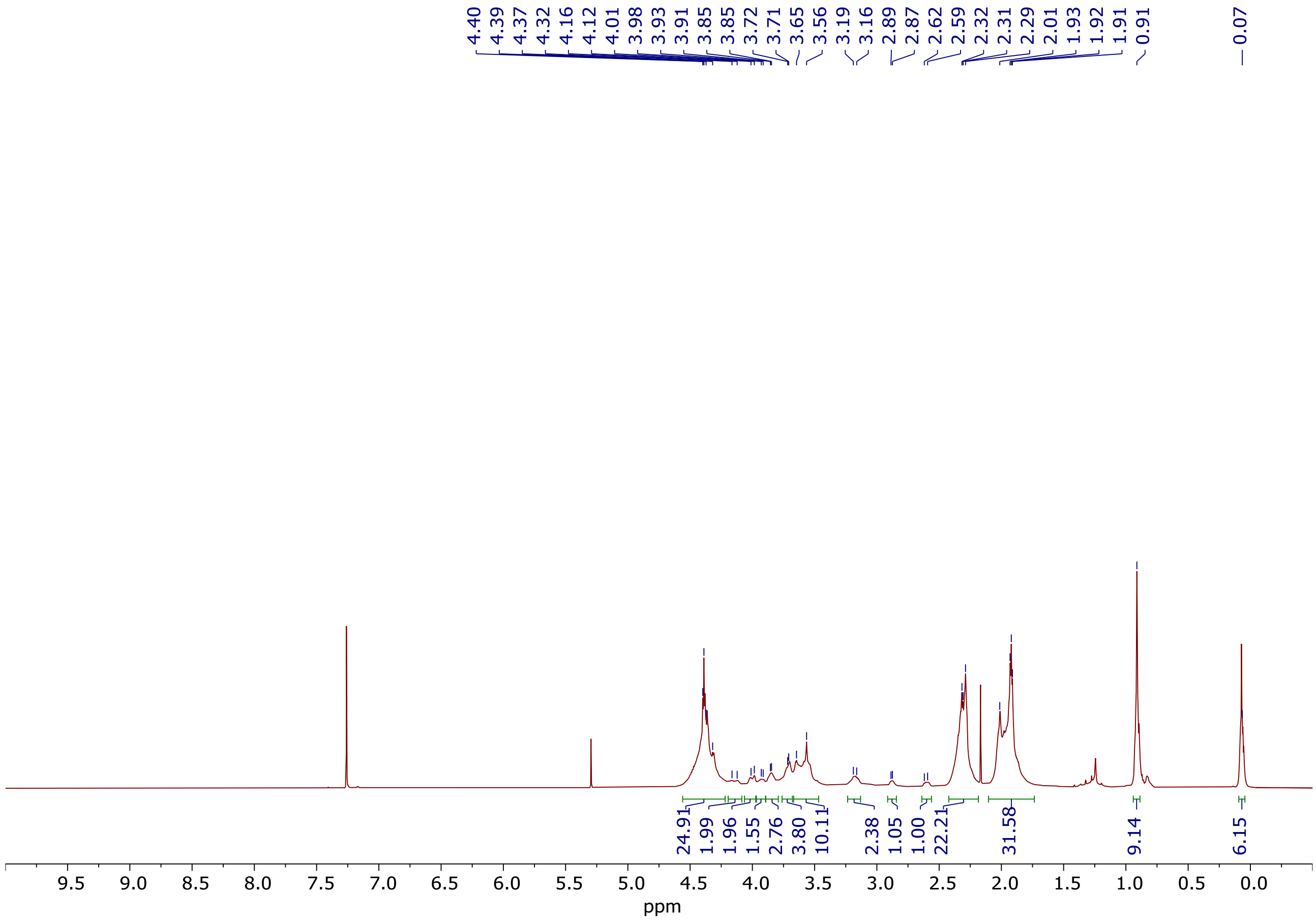

Figure S33. *¹H-NMR spectrum of compound **15** (DMSO-d$_6$, 700 MHz).*

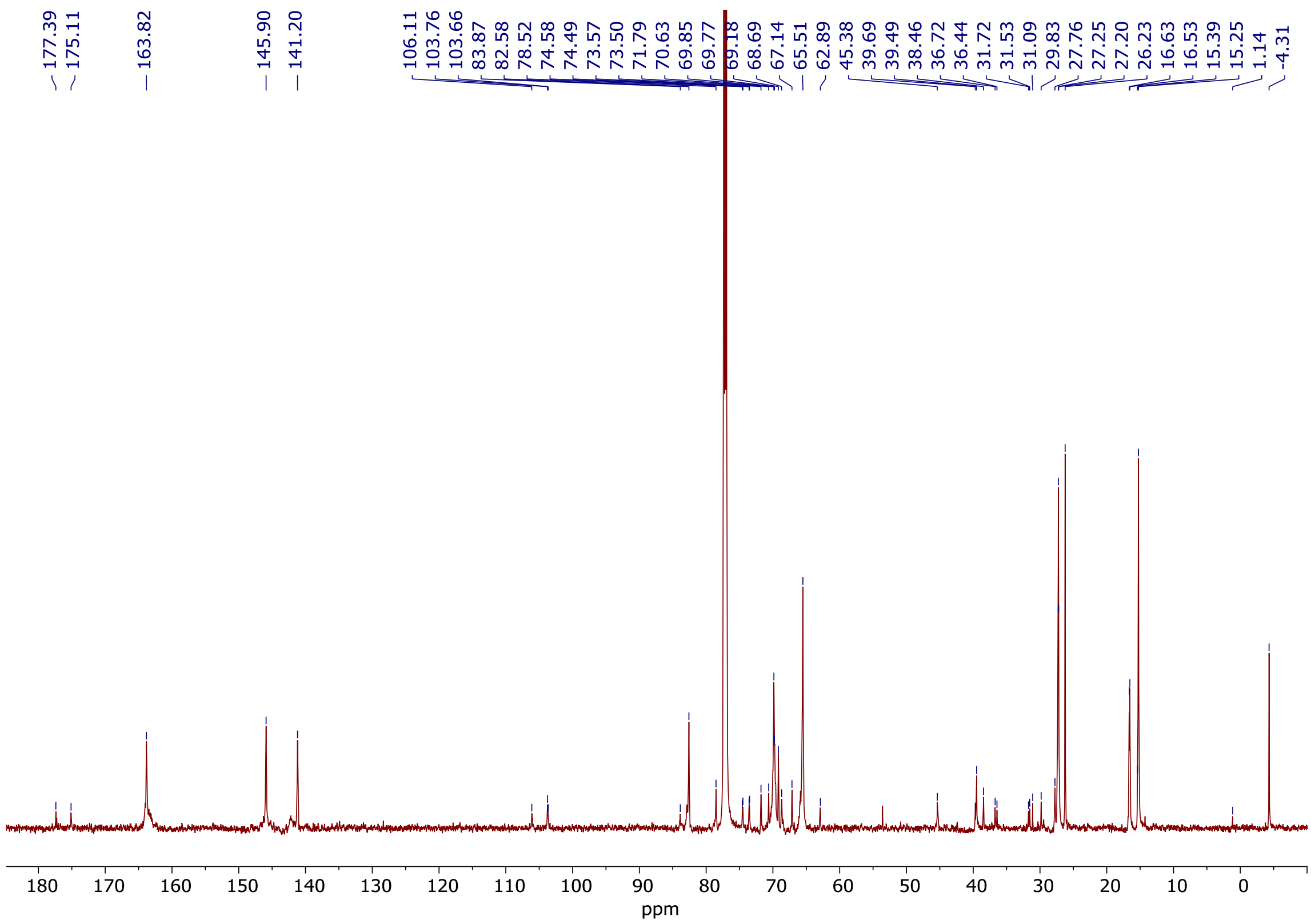

Figure S34. *¹³C-NMR spectrum of compound **15** (DMSO-d$_6$, 176 MHz).*

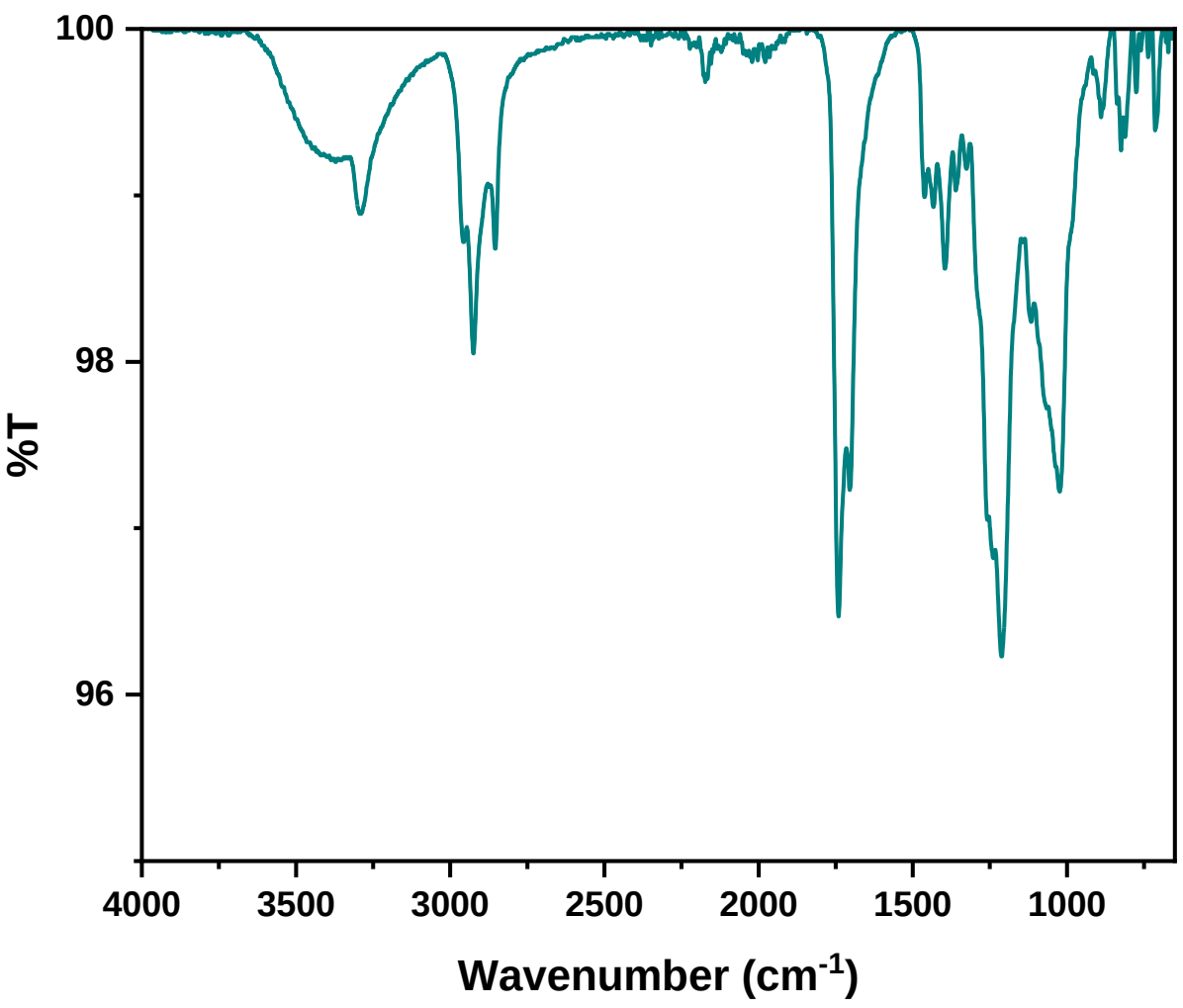

Figure S35. *FT-IR of compound **15**.*

Compound 16

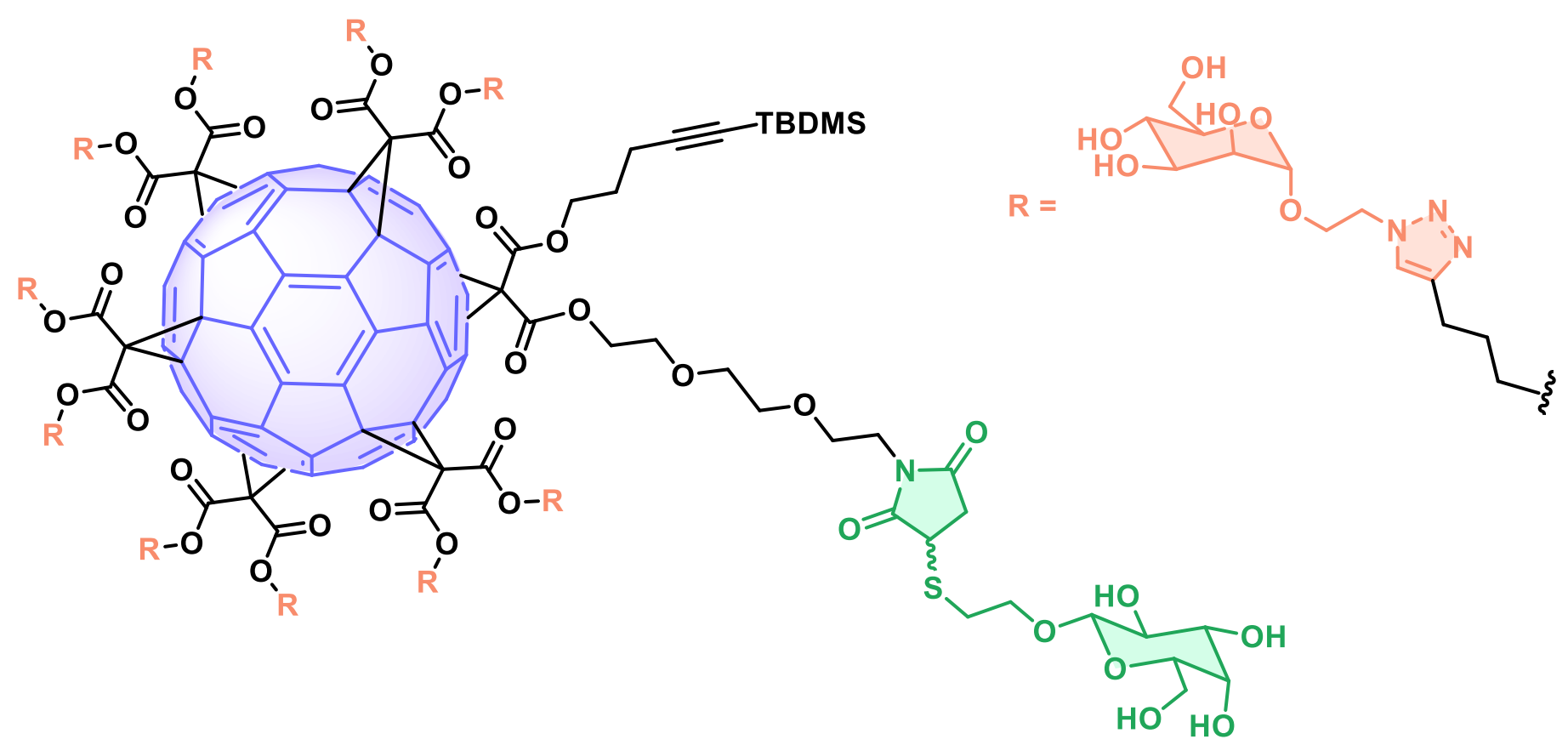

A mixture of the hexakis-adduct **15** (87 mg, 0.033 mmol, 1 eq), mannose-N_3 **11** (148 mg, 0.596 mmol, 18 eq), $CuBr\cdot S(CH_3)_2$ (34 mg, 0.166 mmol, 5 eq), sodium ascorbate (49 mg, 0.247 mmol, 7.5 eq.) and a piece of copper metal wire in DMSO (1.5 mL) was deoxygenated and kept under argon atmosphere with vigorous stirring for 72 h. The solution was filtered through a QuadraSil® Mercaptopropyl column, and then CH_3OH was added to the mixture. The obtained precipitate was centrifuged (6000 r.p.m., 10 min) and washed three times with CH_3OH, then dried under high vacuum to give compound **16** (136 mg, 80%) as a brown solid.

^{1}H NMR (700 MHz, DMSO-d_6) δ 7.88 (s, 10H, $CH_{triazole}$), 4.81 – 4.63 (m, 22H, OH), 4.62 – 4.35 (m, 52H, 10x$CH_{1,man}$, $OCH_2C\underline{H}_2N_{man}$, 22xOH), 4.34 – 4.03 (25H, 12xCH_2OCO, $CH_{1,gal}$), 3.96 – 3.83 (m, 12H, $OC\underline{H}_2CH_2N_{man}$, $OC\underline{H}_2CH_2S_{gal}$, CH_{succ}), 3.80 – 3.68 (m, 11H, $OC\underline{H}_2CH_2N_{man}$, $OC\underline{H}_2CH_2S_{gal}$), 3.62 – 3.54 (m, 11H, 10x$C\underline{H}_2OH_{man}$, $C\underline{H}_2OH_{gal}$), 3.53 – 3.34 (m, 54H, 4x$C\underline{H}_2O$, 30xCH_{man}, 3xCH_{gal}, N_{succ}-$C\underline{H}_2$, 10x$C\underline{H}_2OH$, $C\underline{H}_2OH_{gal}$), 3.17 – 3.02 (m, 13H, 10xCH_{man}, CH_{gal}, $CH_{2,succ}$), 2.93 – 2.57 (m, 22H, SCH_2, 10x$COOCH_2CH_2C\underline{H}_2$), 2.34 – 2.17 (m, 2H, TBDMS-C≡$CC\underline{H}_2$), 2.08 – 1.70 (m, 22H, 11x$COOCH_2C\underline{H}_2CH_2$), 0.89 – 0.70 (m, 9H, tBuSi), 0.07 – 0.11 (m, 6H, 2x$C\underline{H}_3Si$). **^{13}C NMR** (176 MHz, DMSO-d_6) δ 176.8, 174.9 (CO_{succ}), 162.7 (CO), 146.1 ($C_{triazole}$), 144.9, 140.6 ($C_{sp2,fullerene}$), 122.7 ($CH_{triazole}$), 106.8 (TBDMS-C≡$\underline{C}$), 103.4 ($CH_{1,gal}$), 99.7 ($CH_{1,man}$), 83.2 (TBDMS-$\underline{C}$≡C), 75.1 (CH_{gal}), 74.0 (CH_{man}), 73.3 (CH_{gal}), 70.7 (CH_{man}), 70.4 (CH_{gal}), 70.0 (CH_{man}), 69.5, 69.3 (CH_2O), 68.6 ($C_{sp3,fullerene}$), 68.4 ($O\underline{C}H_2CH_2S$), 68.0 (CH_{gal}), 66.6 (CH_{man}), 66.2, 65.5 ($COOCH_2$), 64.7 ($O\underline{C}H_2CH_2N_{man}$), 61.0 ($CH_2OH_{man}$), 60.3 ($CH_2OH_{gal}$), 49.3 ($OCH_2\underline{C}H_2N_{man}$), 45.4 ($C_q$), 37.6 ($N_{succ}$-$\underline{C}H_2$), 36.0, 35.8 ($CH_{2,succ}$), 30.4, 30.3 ($SCH_2$) 27.60($COOCH_2\underline{C}H_2CH_2$), 26.7 (TBDMS-C≡$CCH_2\underline{C}H_2$), 25.8 (tBuSi), 21.4 ($COOCH_2CH_2\underline{C}H_2$), 16.1 ($C_{q,tBu}$), 15.6 (TBDMS-C≡$C\underline{C}H_2$), -4.7 ($\underline{C}H_3Si$).

FTIR-ATR (υ, cm^{-1}): 3345 (OH), 2933 (C-H), 1736 (C=O), 1221 (C-O), 1005 (C-O-C).

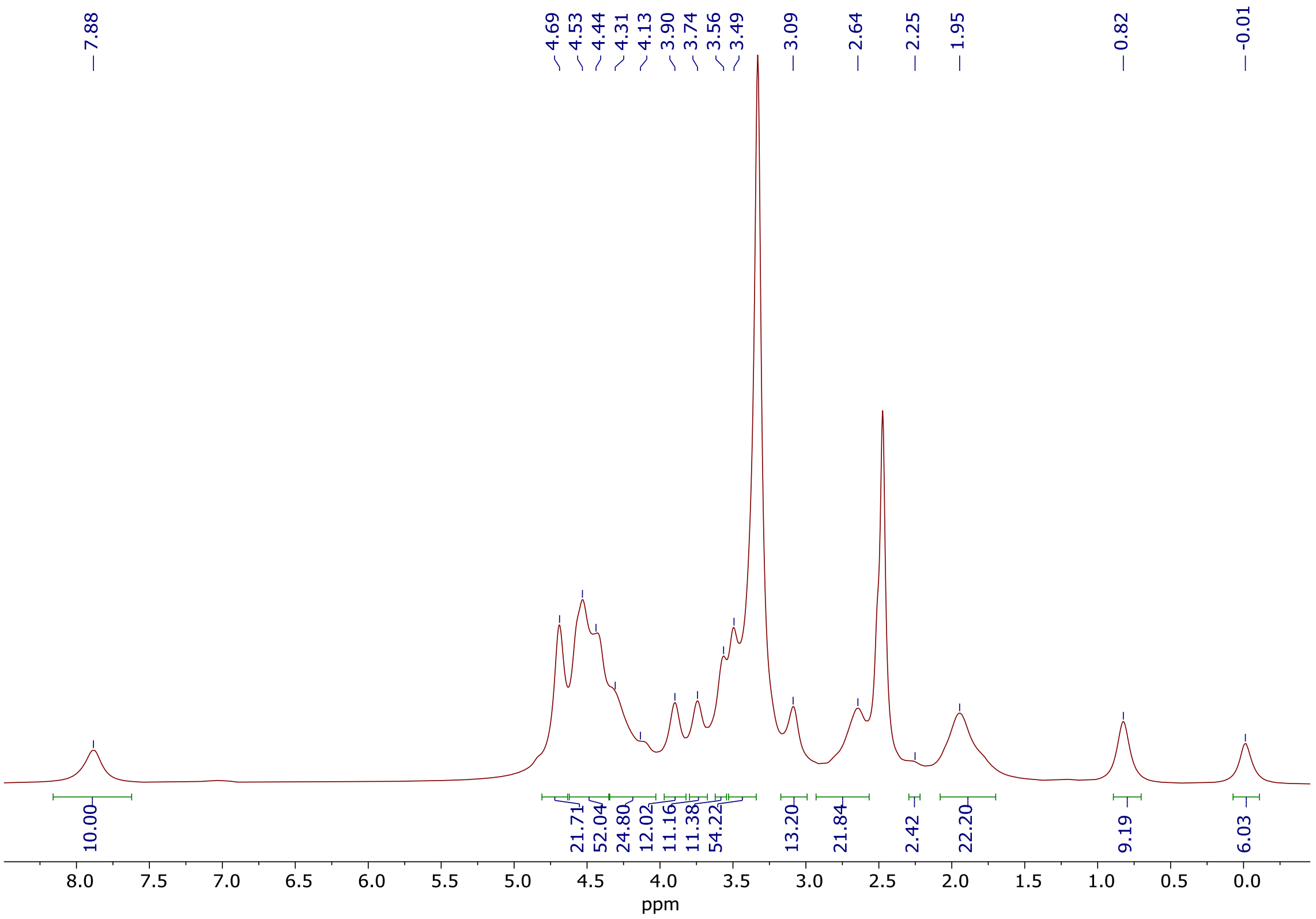

Figure S36. *¹H-NMR spectrum of compound **16** (DMSO-d₆, 700 MHz).*

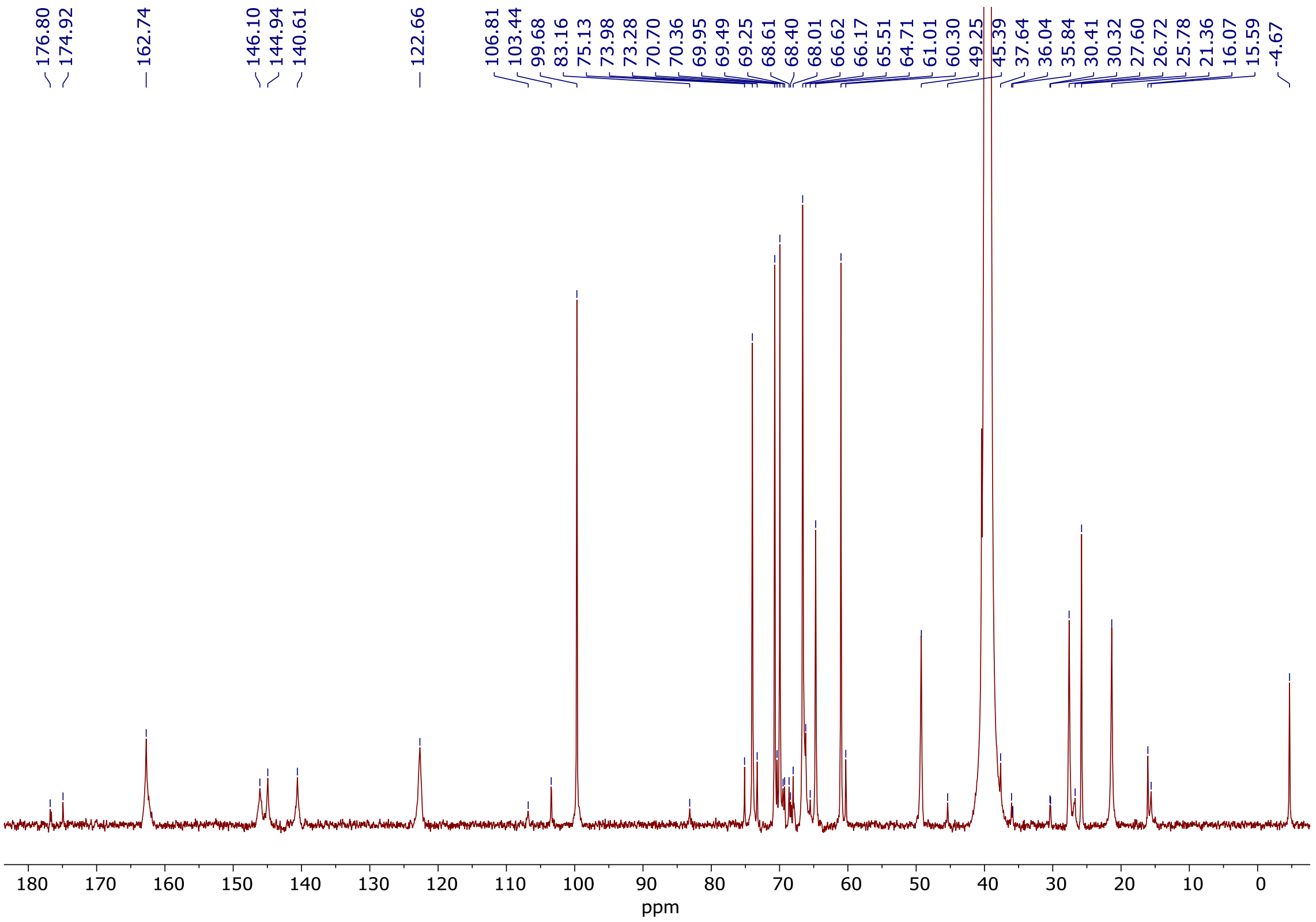

Figure S37. *^{13}C-NMR spectrum of compound **16** (DMSO-d$_6$, 176 MHz).*

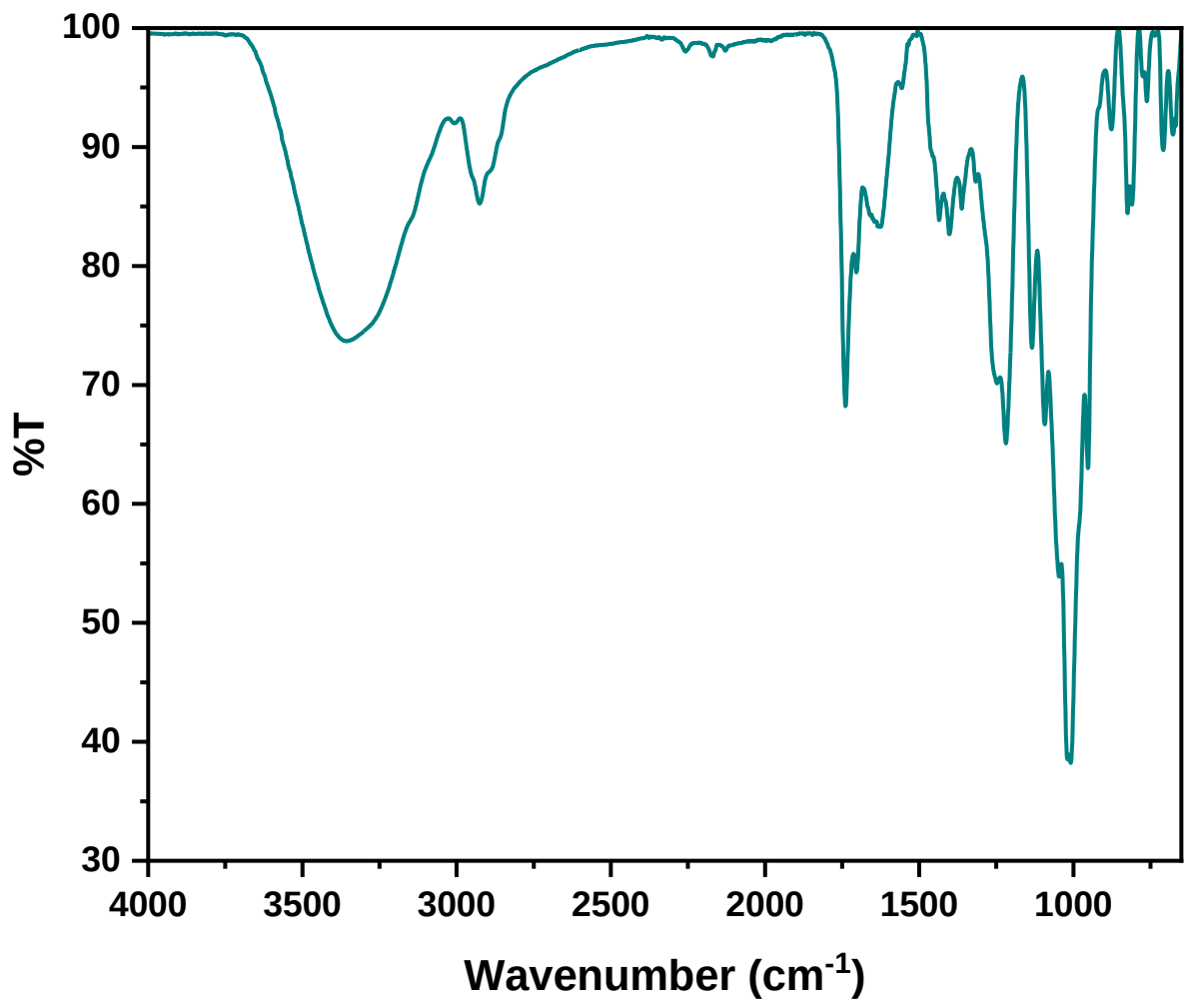

Figure S38. *FT-IR spectrum of compound* ***16.***

Compound 16 - *One-pot procedure*

Galactose-SH **14** (3 mg, 0.013 mmol, 2 eq) was added to a solution of the hexakis-adduct **5** (15 mg, 0.0063 mmol, 1 eq) and Et_3N (1.76 µL, 0.013 mmol, 1 eq) in DMSO (1 mL) under argon atmosphere and the mixture was stirred at room temperature for 24 hours. After that time, mannose-N_3 **11** (26 mg, 0.103 mmol, 18 eq), CuBr·$S(CH_3)_2$ (5.9 mg, 0.028 mmol, 5 eq.), sodium ascorbate (8.5 mg, 0.043 mmol, 7.5 eq) and a piece of copper metal wire were added to the mixture and deoxygenated. The solution was kept under argon atmosphere with vigorous stirring for 72 h. Then, the crude was filtered through a QuadraSil® Mercaptopropyl column and purified by size-exclusion chromatography (Sephadex G-50, H_2O/MeOH 9:1), obtaining compound **16** (28 mg, 86%) as a brown solid.

Compound 19

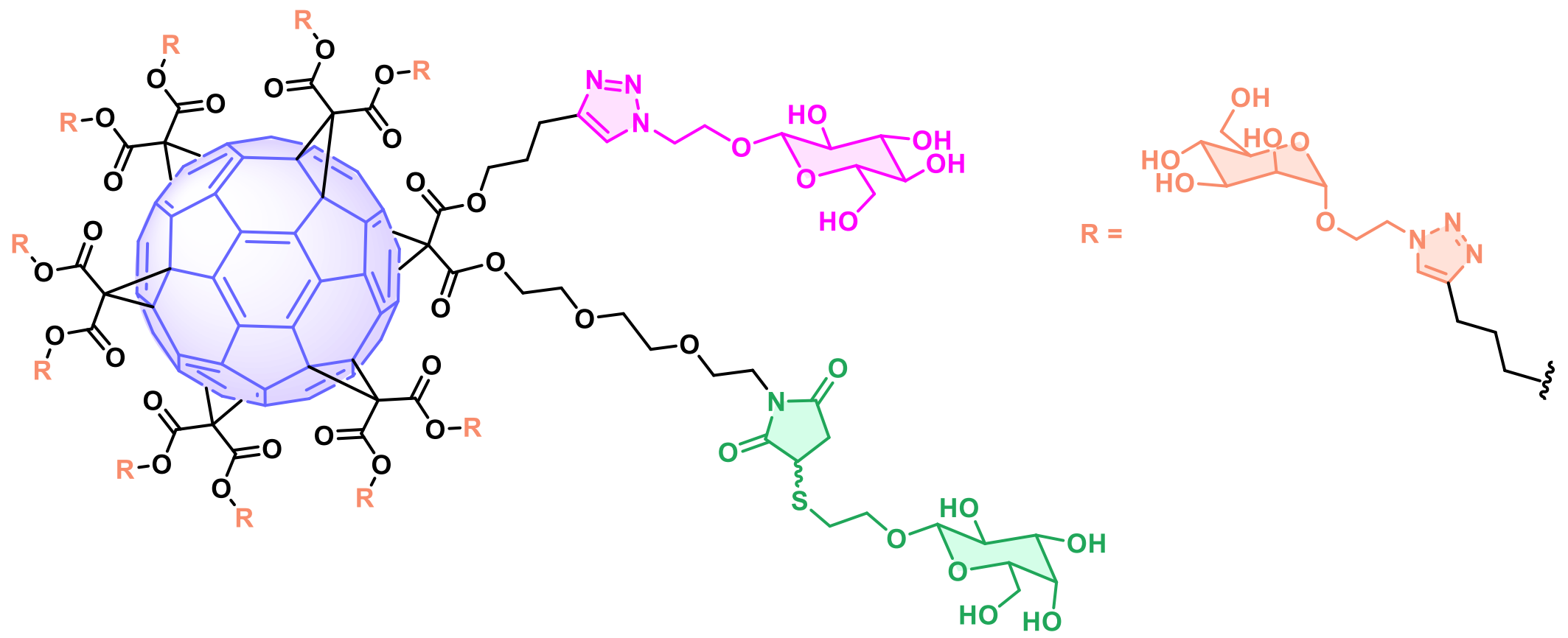

TBAF (1M in THF, 0.062 mL, 8 eq) was added to a solution of the glycofullerene **16** (40 mg, 0.0078 mmol, 1 eq) in DMSO (1 mL) under argon atmosphere. The solution was heated to 30 ºC using a sand bath and kept under stirring for 24 h. Then, glucose-N_3 **17** (2.4 mg, 0.0097 mmol, 1.25 eq), CuBr·$S(CH_3)_2$ (1.6 mg, 0.0078 mmol, 1 eq), sodium ascorbate (2.3 mg, 0.012 mmol, 1.5 eq) and a piece of copper metal wire were added to the mixture and it was deoxygenated and kept under argon atmosphere with vigorous stirring for 72 h. The crude was filtered through a QuadraSil® Mercaptopropyl column and purified by size-exclusion chromatography (Sephadex G-50, H_2O/MeOH 9:1), obtaining compound **19** (14 mg, 34%) as a brown solid.

^{1}H NMR (700 MHz, DMSO-d_6) δ 7.95 – 7.87 (s, 1H, $CH_{triazole,glc}$), 7.87 – 7.74 (s, 10H, $CH_{triazole,man}$), 4.74 – 4.67 (m, 24H, OH), 4.60 – 4.58 (m, 10H, 10x$CH_{1,man}$), 4.56 – 4.53 (m, 11H, 10x$OCH_2C\underline{H}_2N_{man}$, $OCH_2C\underline{H}_2N_{glc}$), 4.51 – 4.48 (m, 11H, 10x$OCH_2C\underline{H}_2N_{man}$, $OCH_2C\underline{H}_2N_{glc}$), 4.46 – 4.41 (m, 24H, OH), 4.38 – 4.24 (m, 24H, 12xCH_2OCO), 4.20 – 4.19 (m, 1H, $CH_{1,gal}$), 4.13 – 4.10 (m, 1H, $CH_{1,glc}$), 3.93 – 3.87 (m, 13H, 10x$OC\underline{H}_2CH_2N_{man}$, $OC\underline{H}_2CH_2S_{gal}$, $OC\underline{H}_2CH_2N_{glc}$, CH_{succ}), 3.77 – 3.72 (m, 12H, 10x$OC\underline{H}_2CH_2N_{man}$, $OC\underline{H}_2CH_2S_{gal}$, $OC\underline{H}_2CH_2N_{glc}$), 3.60 – 3.56 (m, 12H, 10x$C\underline{H}_2OH_{man}$, $C\underline{H}_2OH_{gal}$, $C\underline{H}_2OH_{glc}$), 3.53 – 3.49 (m, 12H, 10xCH_{man}, CH_{gal}, CH_{glc}), 3.43 – 3.37 (m, 38H, 2xCH_{gal}, 2xCH_{glc}, N_{succ}-$C\underline{H}_2$, 10x$C\underline{H}_2OH$, $C\underline{H}_2OH_{gal}$, $C\underline{H}_2OH_{glc}$, 20xCH_{man}), 3.13 – 3.08 (m, 12H, 10xCH_{man}, $CH_{2,succ}$), 3.04 – 3.01 (m, 1H, CH_{gal}), 2.97 – 2.93 (m, 1H, CH_{glc}), 2.71 – 2.57 (m, 24H, SCH_2, 11x$COOCH_2CH_2C\underline{H}_2$), 2.00 – 1.89 (m, 22H, 11x$COOCH_2C\underline{H}_2CH_2$). **^{13}C NMR** (176 MHz, DMSO-d_6) δ 145.7, 122.3, 99.8, 74.1, 70.8, 70.1, 66.7, 64.9, 61.1, 49.2, 27.7, 21.4.

FTIR-ATR (υ, cm^{-1}): 3343 (OH), 2919 (C-H), 1737 (C=O), 1216 (C-O), 1019 (C-O-C).

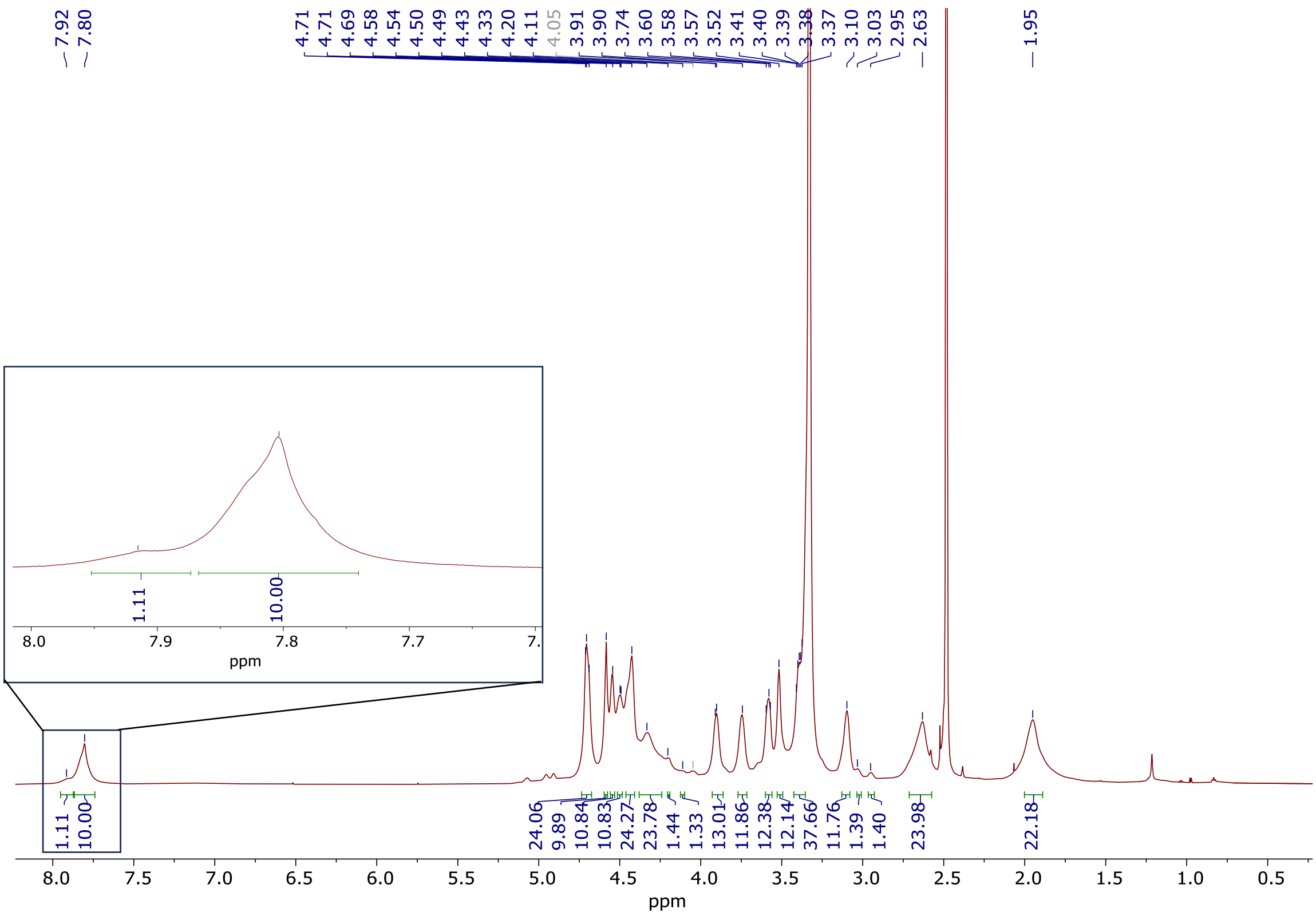

Figure S39. *^{1}H-NMR spectrum of compound **19** (DMSO-d_6, 700 MHz).*

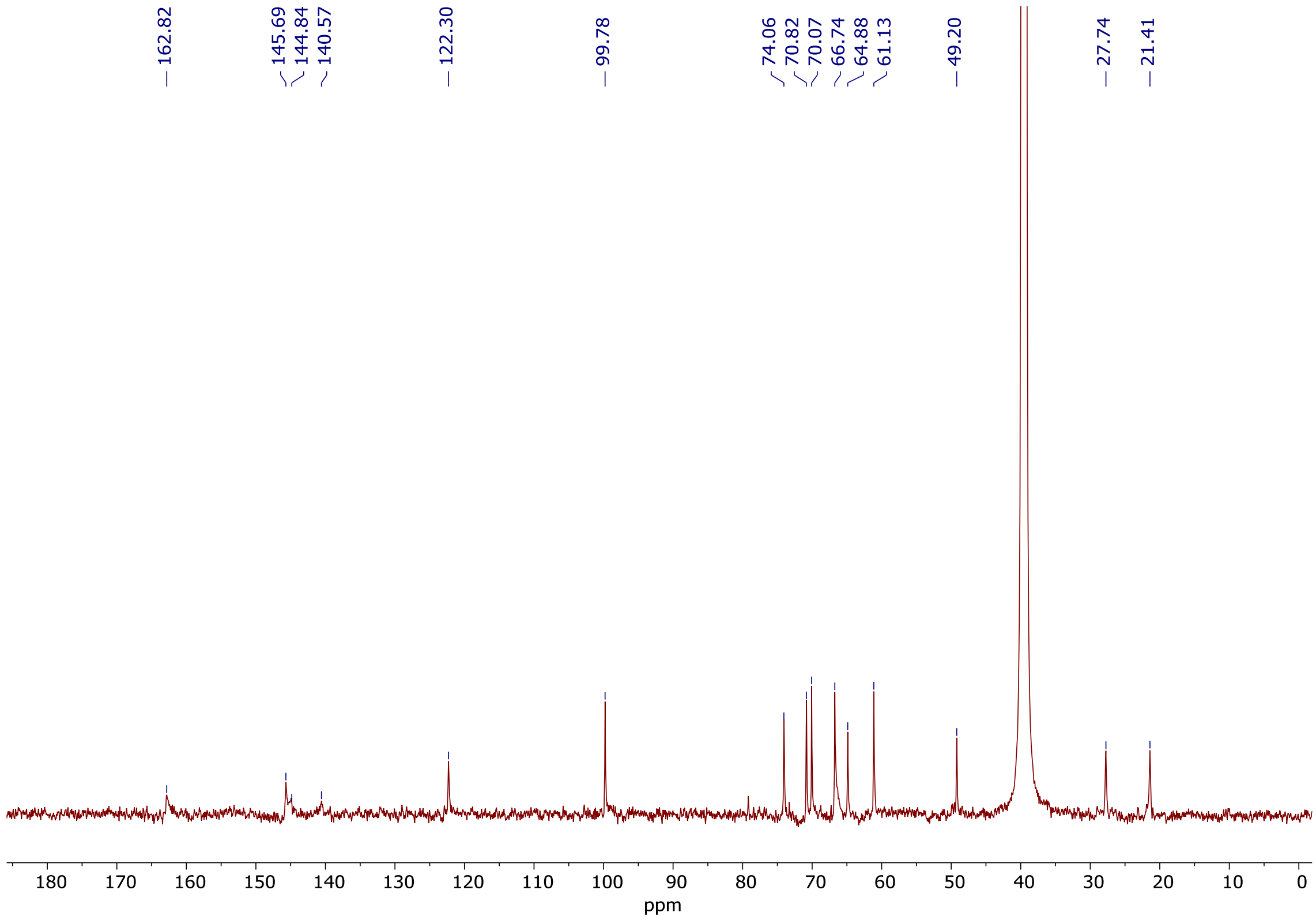

Figure S40. ^{13}C-NMR spectrum of compound **19** (DMSO-d_6, 176 MHz).

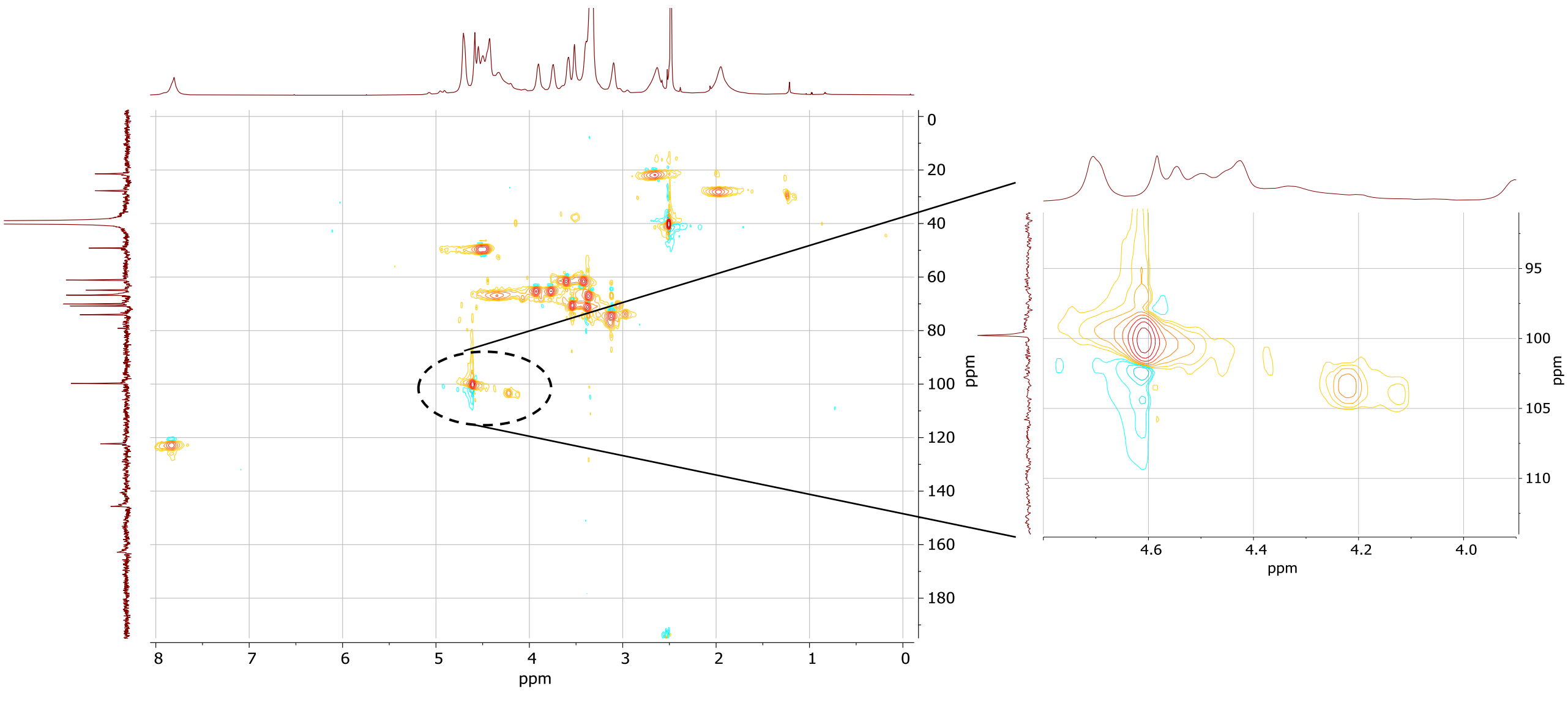

Figure S41. ^{1}H-^{13}C HSCQ NMR spectrum of compound **19** on the left (DMSO-d_6, 700 MHz) and zoom on the anomeric carbons region on the right. Red/orange and blue/cyan contours represent positive and negative contour levels of the same cross-peaks, respectively.

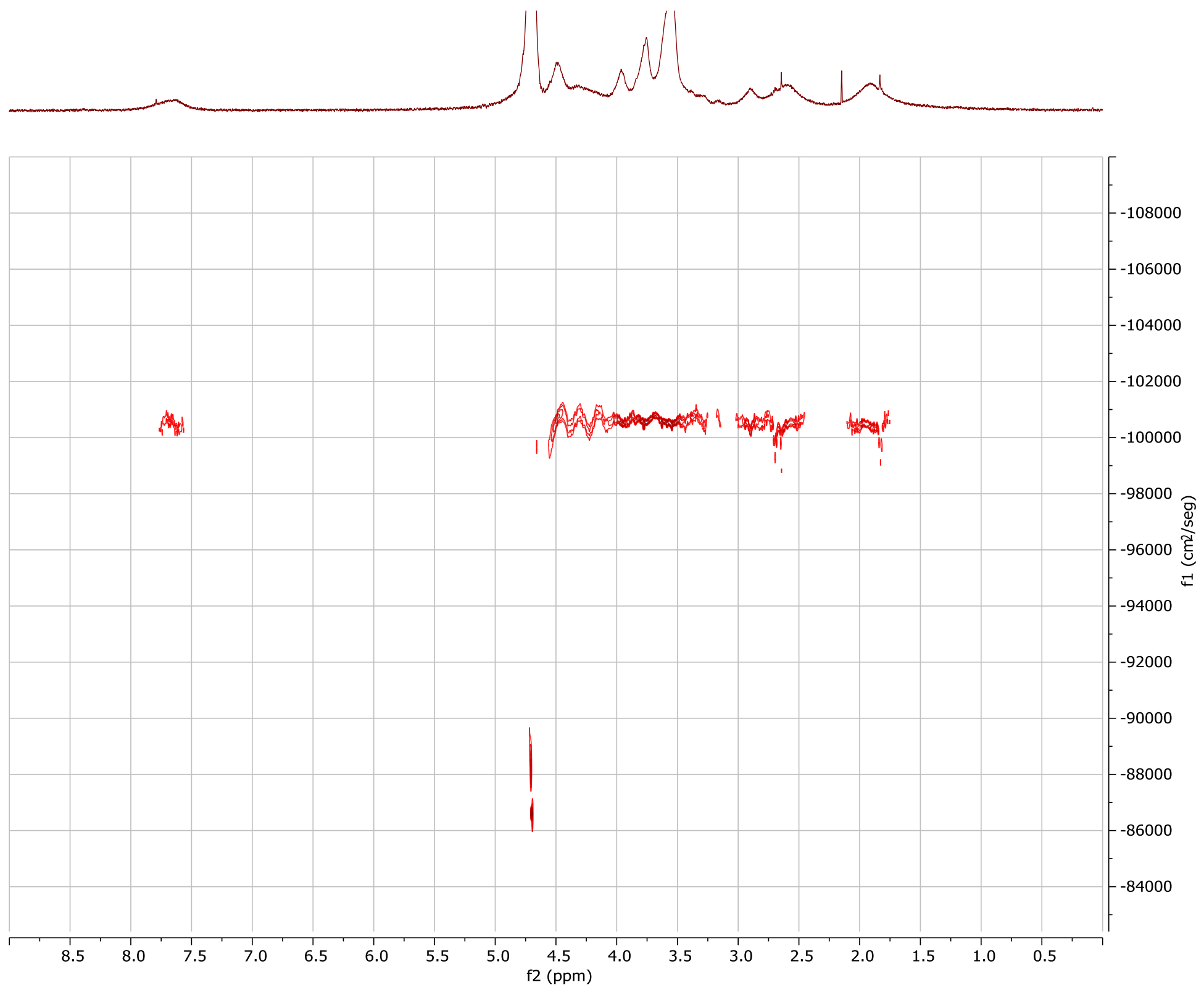

Figure S42. *DOSY-NMR spectrum of compound **19** (D_2O, 700 MHz).*

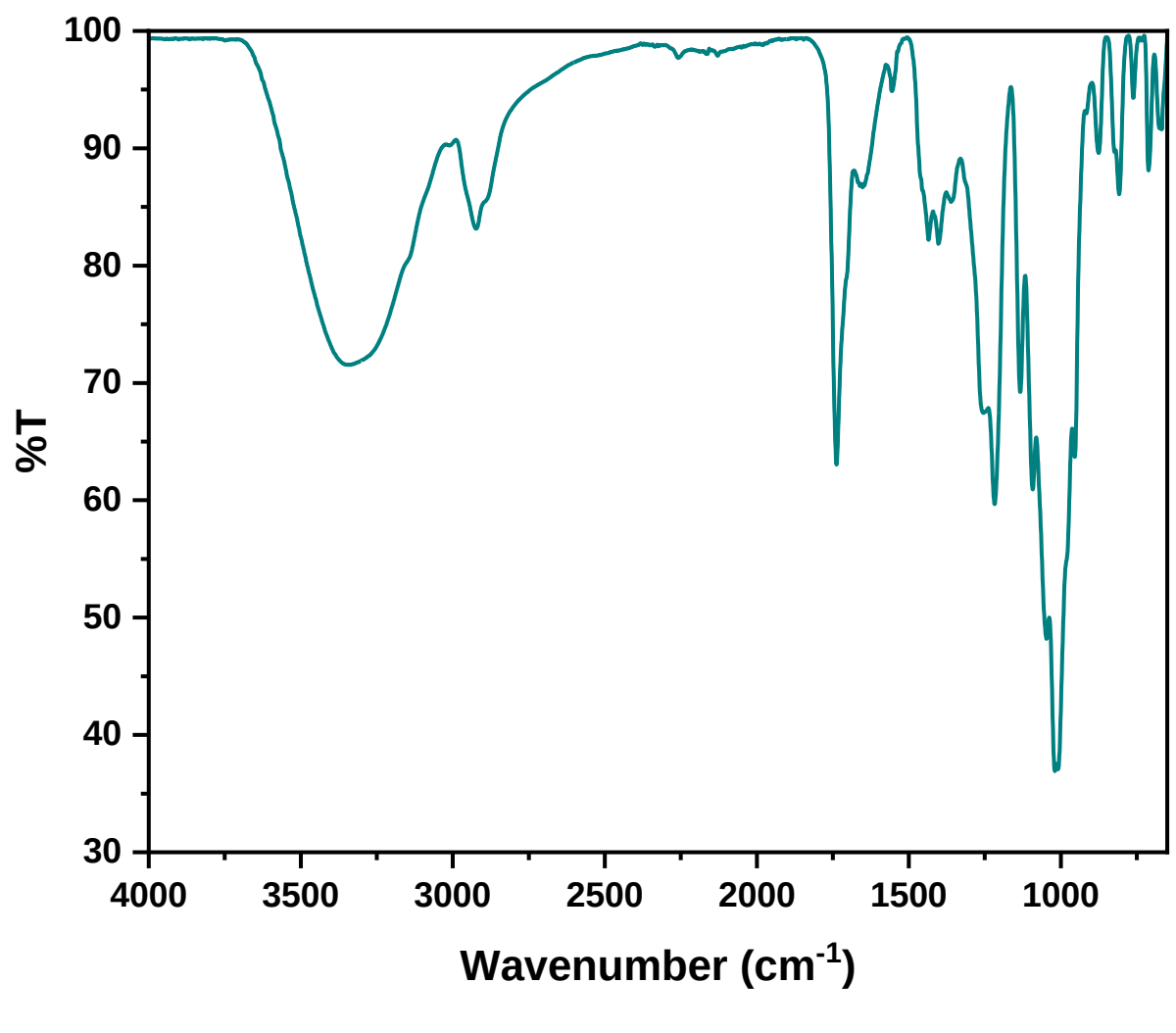

Figure S43*. FT-IR spectrum of compound* ***19****.*

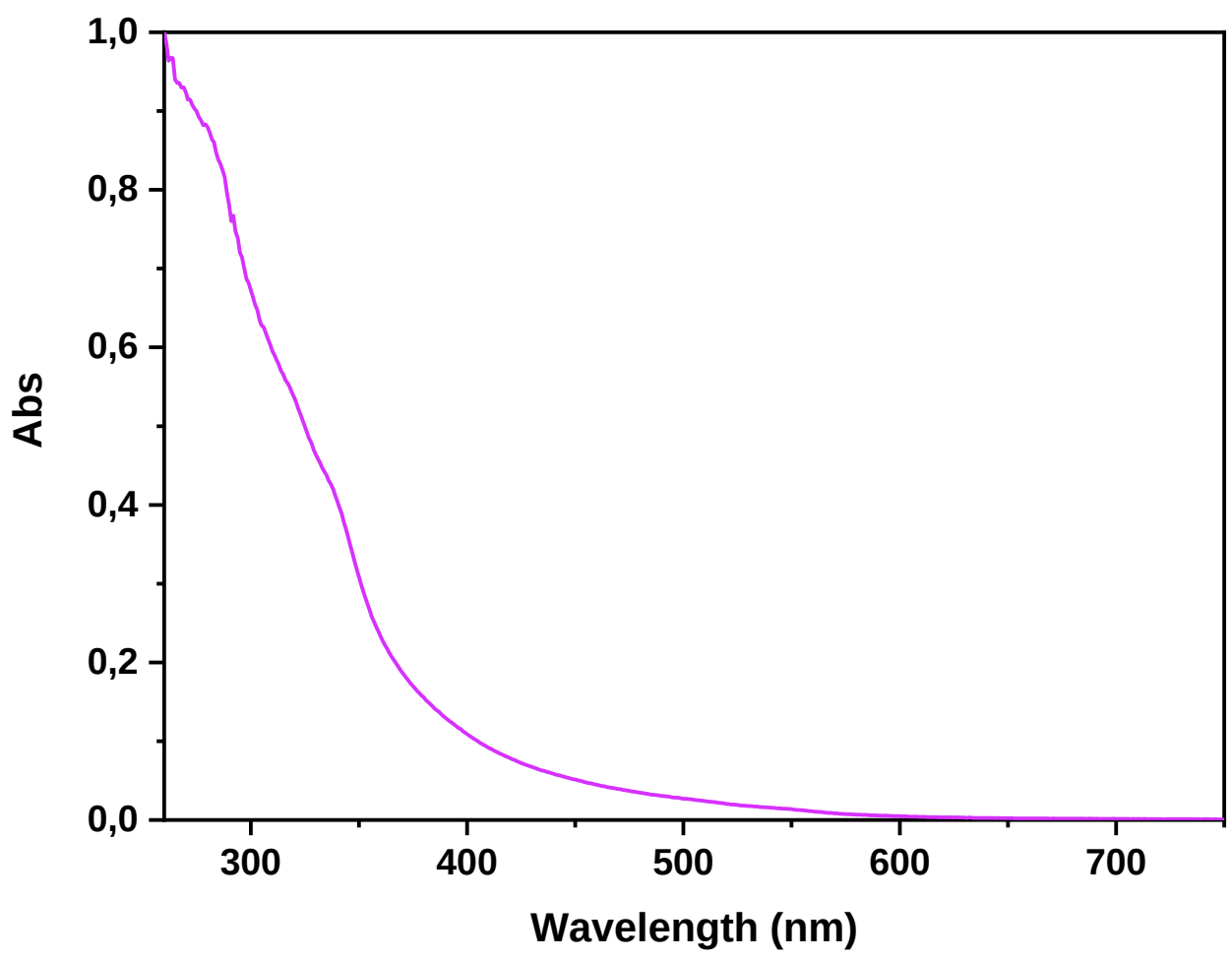

Figure S44. *UV-Vis spectrum of compound* ***19*** *(H_2O, 3.4x10^{-5} M).*

Compound 20

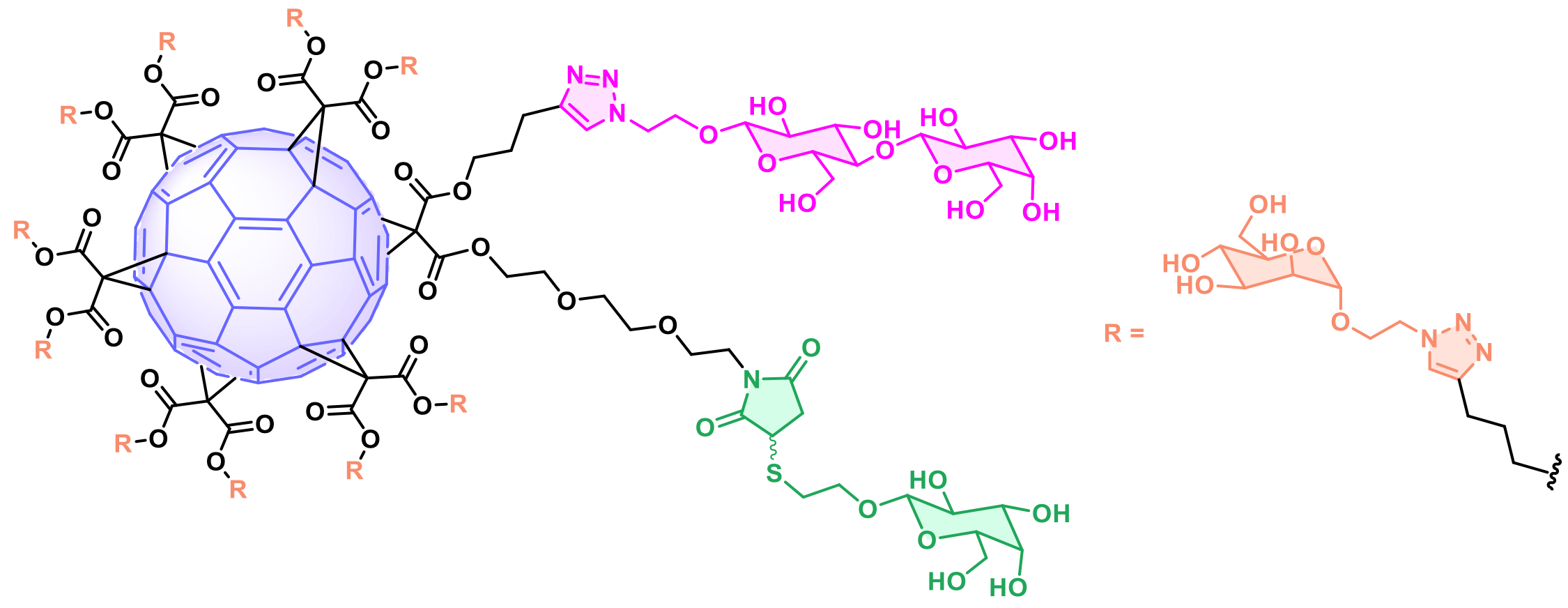

TBAF (1M in THF, 0.154 mL, 8 eq) was added to a solution of the glycofullerene **16** (98.7 mg, 0.019 mmol, 1 eq) in DMSO (1.5 mL). The solution was heated to 30 ºC and kept under stirring for 24 h. Then, lactose-N_3 **18** (9.7 mg, 0.024 mmol, 1.25 eq), CuBr·S(CH_3)$_2$ (3.9 mg, 0.019 mmol, 1 eq), sodium ascorbate (5.6 mg, 0.028 mmol, 1.5 eq) and a piece of copper metal wire in DMSO (1 mL) was deoxygenated and kept under argon atmosphere with vigorous stirring for 72 h. The crude was filtered through a QuadraSil® Mercaptopropyl column and purified by size-exclusion chromatography (Sephadex G-50, H_2O/MeOH 9:1), obtaining compound **20** (50 mg, 51%) as a brown solid.

^{1}H NMR (700 MHz, DMSO-*d*$_6$) δ 7.92 (s, 1H, $CH_{triazole,lac}$), 7.82 (s, 10H, $CH_{triazole,man}$), 4.79 – 4.69 (m, 26H, OH), 4.63 – 4.58 (m, 21H, 10x$CH_{1,man}$, 10x$OCH_2C\underline{H}_2N_{man}$, $OCH_2C\underline{H}_2N_{lac}$), 4.55 – 4.51 (m, 11H, 10x$OCH_2C\underline{H}_2N_{man}$, $OCH_2C\underline{H}_2N_{lac}$), 4.50 – 4.44 (m, 25H, OH), 4.40 – 4.21 (m, 25H, 12xCH_2OCO, $CH_{1,gal}$), 4.20 – 4.18 (m, 1H, CH_{lac}), 4.13 – 4.10 (m, 1H, CH_{lac}), 3.95 – 3.88 (m, 13H, 10x$OC\underline{H}_2CH_2N_{man}$, $OC\underline{H}_2CH_2S_{gal}$, $OC\underline{H}_2CH_2N_{lac}$, CH_{succ}), 3.78 – 3.72 (m, 12H, 10x$OC\underline{H}_2CH_2N_{man}$, $OC\underline{H}_2CH_2S_{gal}$, $OC\underline{H}_2CH_2N_{lac}$), 3.62 – 3.57 (m, 13H, 10x$C\underline{H}_2OH_{man}$, $C\underline{H}_2OH_{gal}$, 2x$C\underline{H}_2OH_{lac}$), 3.54 – 3.50 (m, 13H, 10xCH_{man}, CH_{gal}, 2xCH_{lac}), 3.47 – 3.40 (m, 41H, 2xCH_{gal}, 4xCH_{lac}, N_{succ}-$C\underline{H}_2$, 10x$C\underline{H}_2OH$, $C\underline{H}_2OH_{gal}$, 2x$C\underline{H}_2OH_{lac}$, 20xCH_{man}), 3.16 – 3.07 (m, 12H, 10xCH_{man}, $CH_{2,succ}$), 3.06 – 3.00 (m, 3H, 2xCH_{lac}, CH_{gal}), 2.75 – 2.56 (m, 24H, SCH_2, 11x$COOCH_2CH_2C\underline{H}_2$), 2.01 – 1.84 (m, 22H, 11x$COOCH_2C\underline{H}_2CH_2$). **^{13}C NMR** (176 MHz, DMSO-*d*$_6$) δ 162.9, 145.7, 145.0, 141.0, 122.4, 103.5,

102.5, 99.8, 80.7, 75.6, 74.9, 74.1, 73.3, 73.1, 70.9, 70.6, 70.1, 68.2, 67.5, 64.9, 61.2, 60.5, 49.3, 27.8, 21.4.

FTIR-ATR (υ, cm^{-1}): 3335 (OH), 2930 (C-H), 1740 (C=O), 1219 (C-O), 1007 (C-O-C).

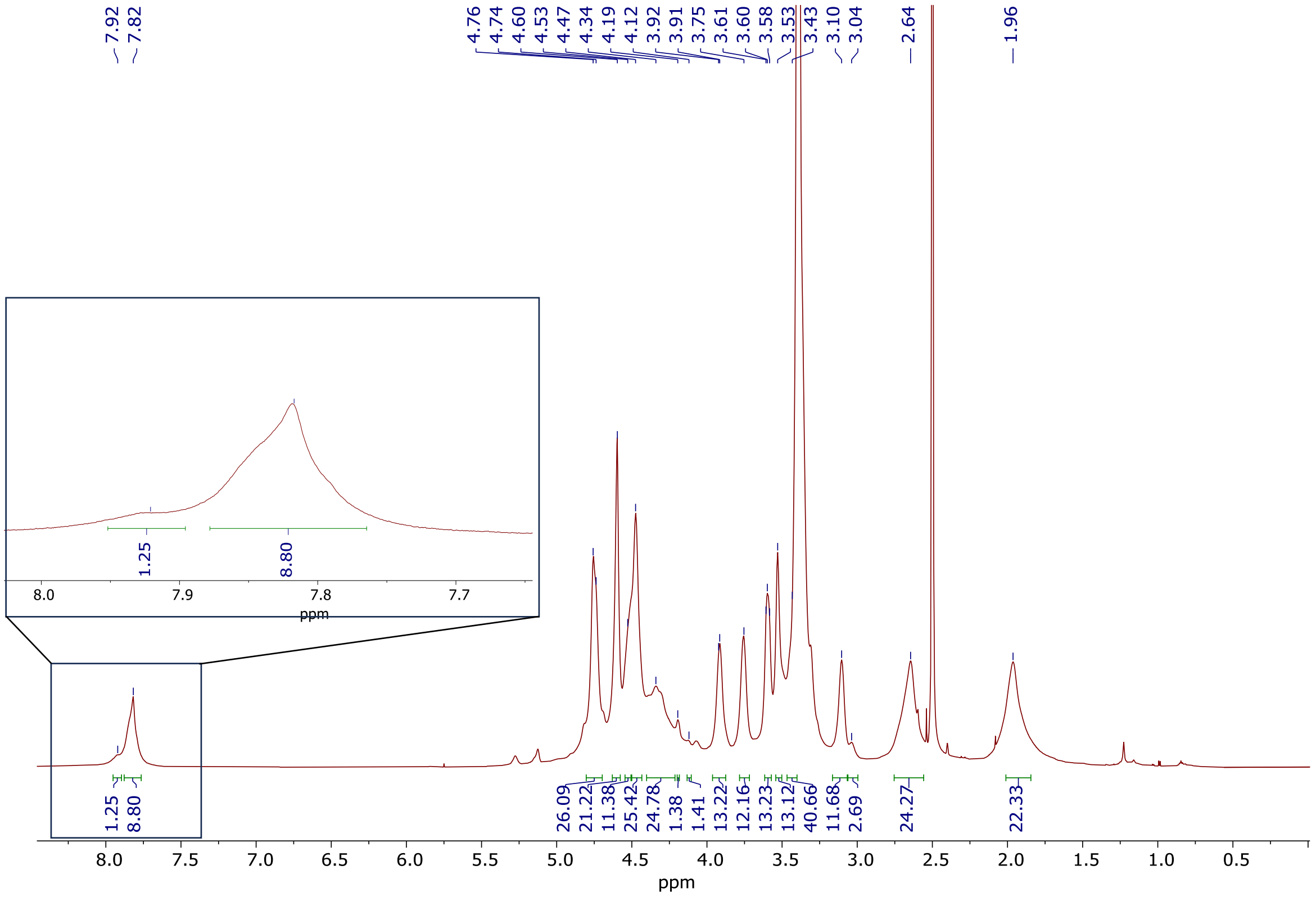

Figure S45. *^{1}H-NMR spectrum of compound **20** (DMSO-d$_6$, 700 MHz).*

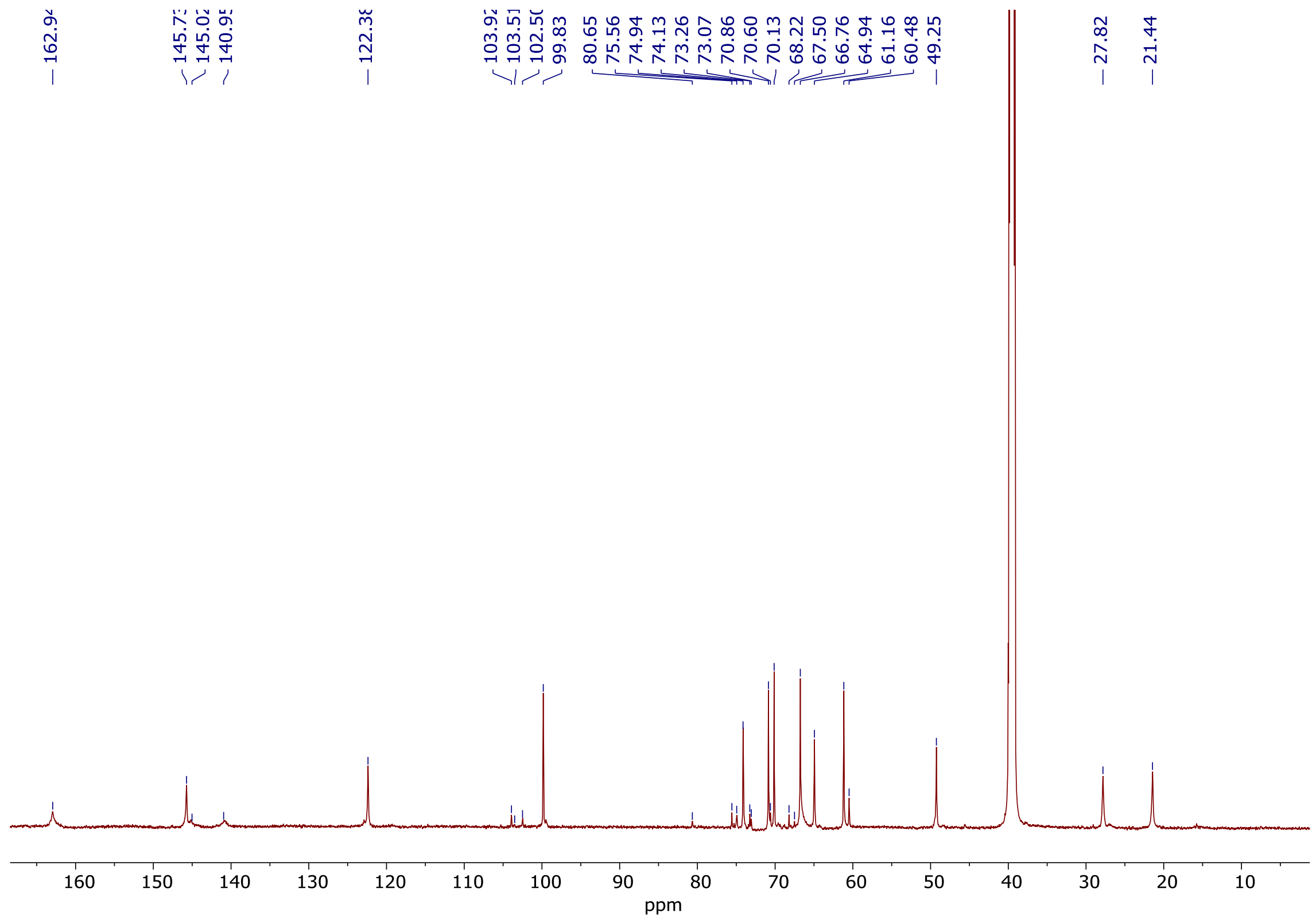

Figure S46. *^{13}C-NMR spectrum of compound **20** (DMSO-d$_6$, 176 MHz).*

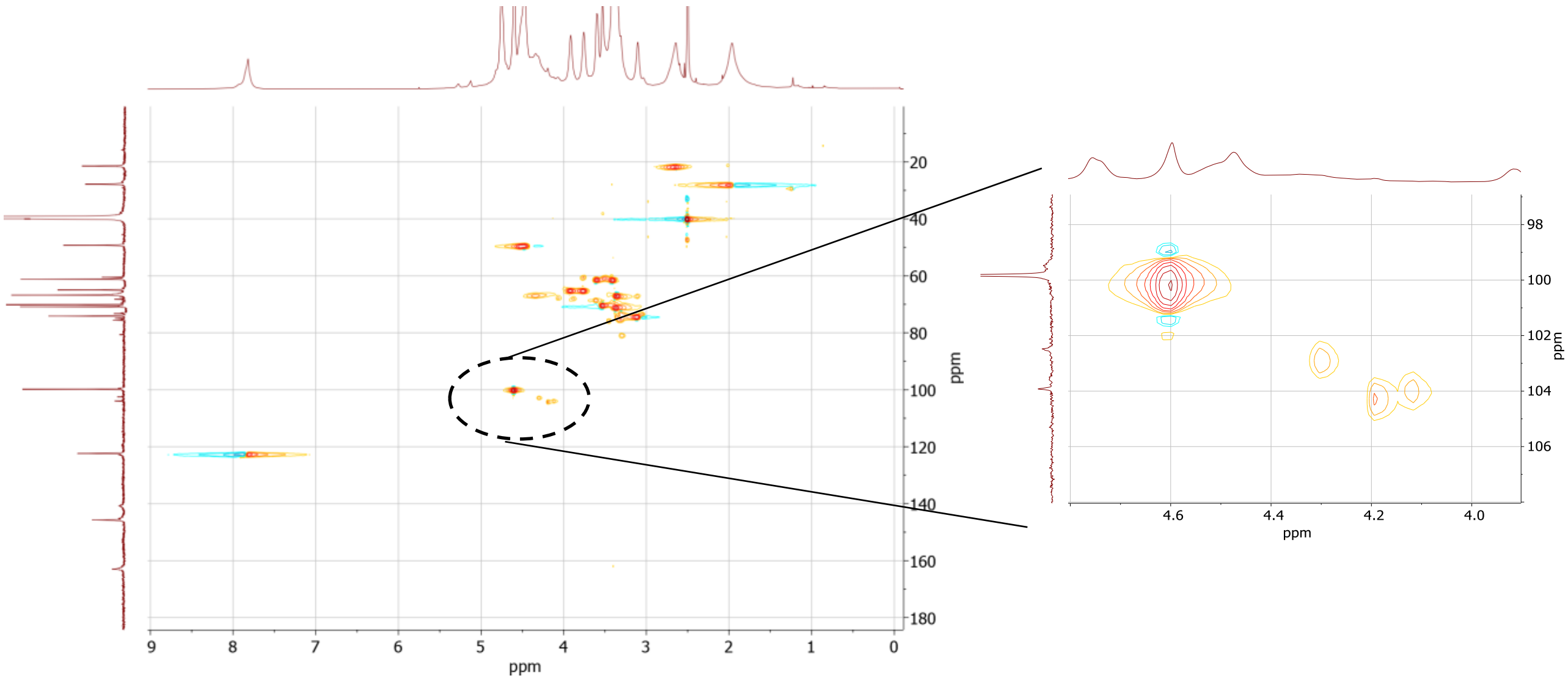

Figure S47. *^{1}H-^{13}C HSCQ NMR spectrum of compound **20** on the left (DMSO-d_6, 700 MHz) and zoom on the anomeric carbons region on the right. Red/orange and blue/cyan contours represent positive and negative contour levels of the same cross-peaks, respectively.*

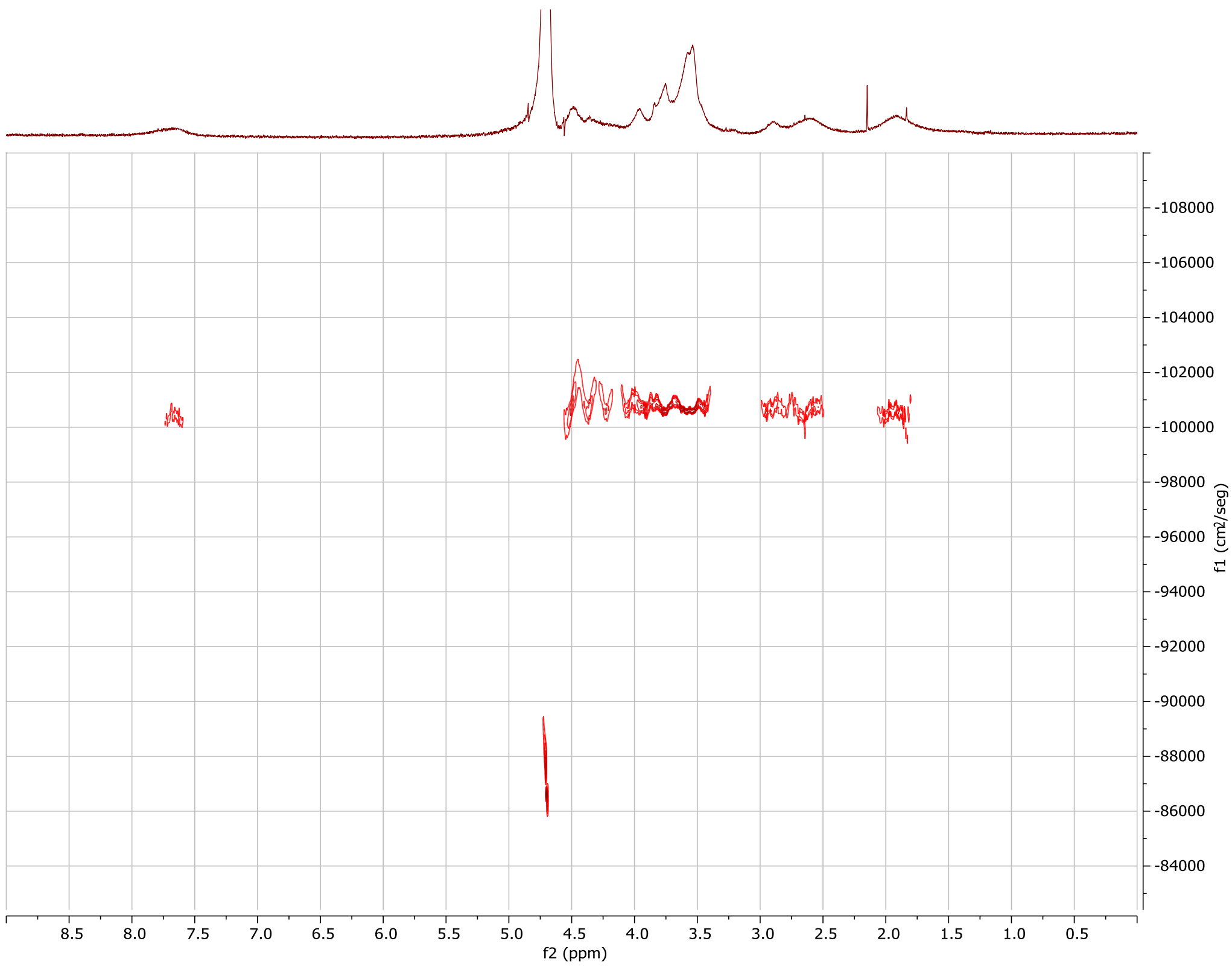

Figure S48. *DOSY-NMR spectrum of compound **20** (D_2O, 700 MHz).*

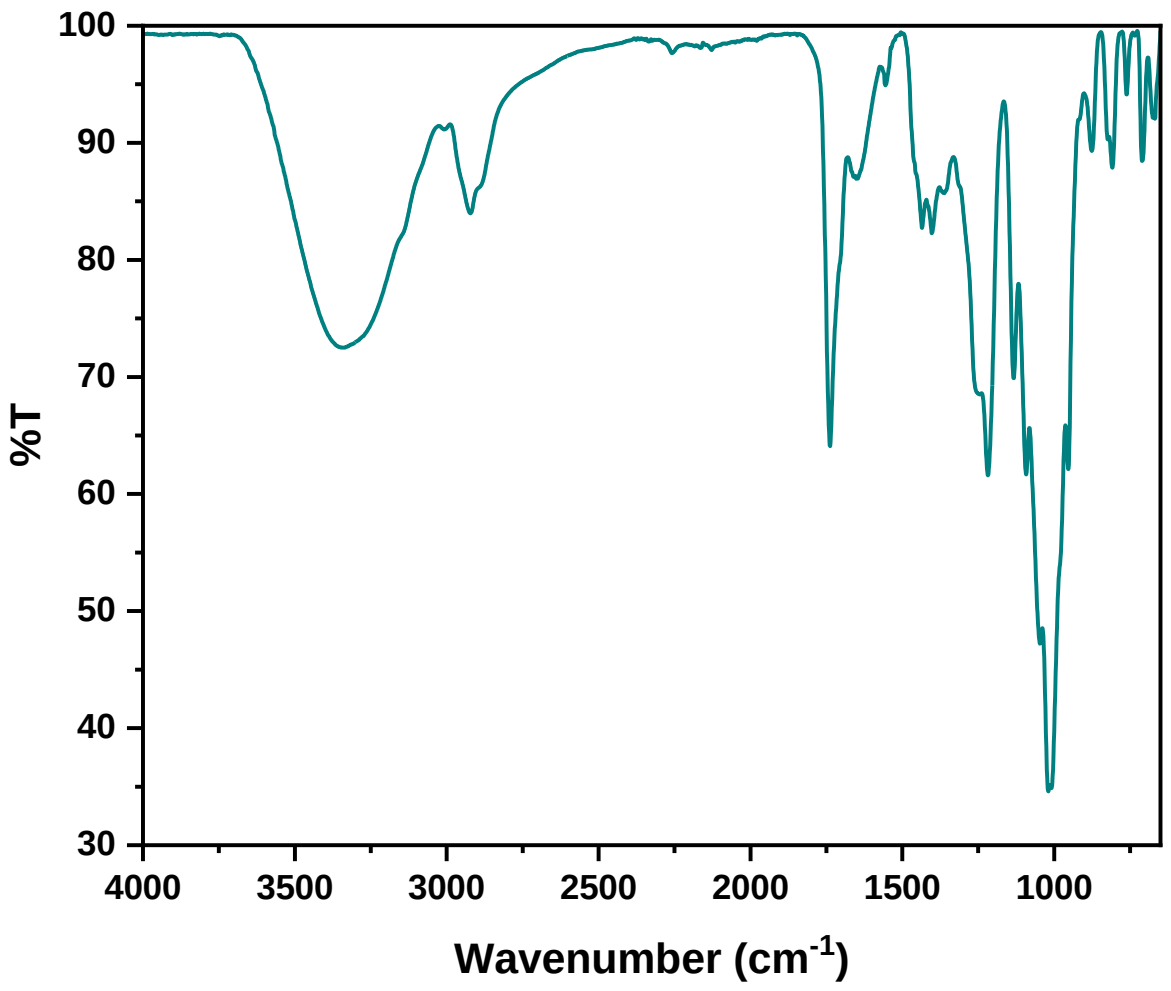

Figure S49. *FT-IR spectrum of compound **20**.*

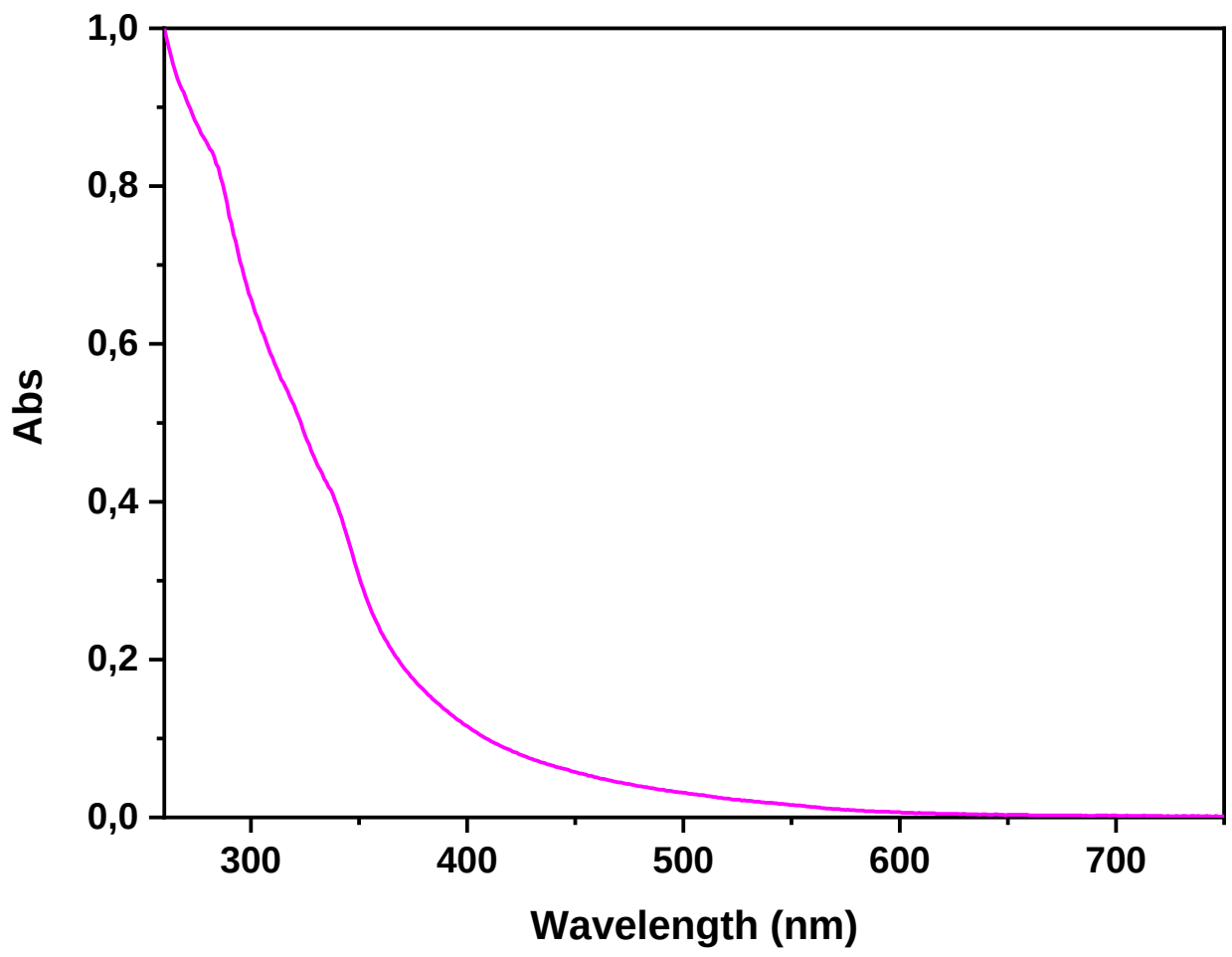

Figure S50. *UV-Vis spectrum of compound **20** (H_2O, 4.4x10^{-5} M).*

References

[1] Y. Min, F. Leng, B. F. Machado, P. Lecante, P. Roblin, H. Martínez, T. Theussl, A. Casu, A. Falqui, M. Barcenilla, S. Coco, B. M. Illescas, N. Martín, M. R. Axet, P. Serp, *Eur. J. Inorg. Chem.* **2020**, *2020*, 4069.

[2] N. Yamazaki, K. Ueno, H. Otuska, *Trans. Mater. Res. Soc. Japan.* **2008,** *33,* 747.

[3] a) E. Arce, P. M. Nieto, V. Díaz, R. G. Castro, A. Bernad, J. Rojo, *Bioconjug Chem.* **2003**, *14*, 817. b) T. K. Lindhorst, S. Kötter, U. Krallmann-Wenzel, S. Ehlers, *J. Chem. Soc. Perkin Trans. 1* **2001**, 823.

[4] K. Neumann, A. Conde-González, M. Owens, A. Venturato, Y. Zhang, J. Geng, M. Bradley, *Macromolecules* **2017**, *50*, 6026.

[5] V. Yarlagadda, M. M. Konai, G. B. Manjunath, C. Ghosh, J. Haldar, *J. Antibiot.* **2015**, *68*, 302.

[6] a) F. J. Muñoz, J. Pérez, A. Rumbero, J. Ignacio Santos, F. Javier Cañada, S. André, H. Gabius, J. Jiménez-Barbero, J. V. Sinisterra, M. J. Hernáiz, *Bioconjug. Chem.* **2009**, *20*, 673. b) J. Y. Hyun, S. Kim, C. Lee, H. S. Lee, I. Shin, *ACS Chem. Biol.* **2021**, *16*, 1930.
